# Plasma fractalkine contributes to systemic myeloid diversity and PD‐L1/PD‐1 blockade in lung cancer

**DOI:** 10.15252/embr.202255884

**Published:** 2023-06-27

**Authors:** Ana Bocanegra, Gonzalo Fernández‐Hinojal, Daniel Ajona, Ester Blanco, Miren Zuazo, Maider Garnica, Luisa Chocarro, Elvira Alfaro‐Arnedo, Sergio Piñeiro‐Hermida, Pilar Morente, Leticia Fernández, Ana Remirez, Miriam Echaide, Maite Martinez‐Aguillo, Idoia Morilla, Beatriz Tavira, Alejandra Roncero, Carolina Gotera, Alfonso Ventura, Nerea Recalde, José G Pichel, Juan José Lasarte, Luis Montuenga, Ruth Vera, Ruben Pio, David Escors, Grazyna Kochan

**Affiliations:** ^1^ Oncoimmunology Group Navarrabiomed, Hospital Universitario de Navarra, Universidad Publica de Navarra (UPNA), IdISNA Pamplona Spain; ^2^ Medical Oncology Department Clinica Universidad de Navarra Madrid Spain; ^3^ Program in Solid Tumors CIMA‐University of Navarre‐IdISNA Pamplona Spain; ^4^ CIBERONC Centro de Investigación Biomédica en Red de Cáncer Madrid Spain; ^5^ Department of Biochemistry and Genetics, School of Sciences University of Navarra‐IdISNA Pamplona Spain; ^6^ Program in Gene Therapy and Regulation of Gene Expression CIMA‐University of Navarra‐IdISNA Pamplona Spain; ^7^ Lung Cancer and Respiratory Diseases Unit, Center for Biomedical Research of La Rioja (CIBIR) Fundación Rioja Salud Logroño Spain; ^8^ Department of Oncology Hospital Universitario de Navarra‐IdISNA Pamplona Spain; ^9^ Cancer Center University of Navarra (CCUN) Pamplona Spain; ^10^ Department of Pathology, Anatomy and Physiology, School of Medicine University of Navarra‐IdISNA Pamplona Spain; ^11^ Pathological Anatomy Service, Hospital Universitario San Pedro Rioja Salud Logroño Spain; ^12^ Pneumology Service Rioja Salud Logroño Spain; ^13^ Fundación Jiménez Díaz Madrid Spain; ^14^ Centro de Salud Salazar‐Ezcároz Navarra Spain; ^15^ Spanish Biomedical Research Networking Centre CIBERES Madrid Spain; ^16^ Program in Immunology and Immunotherapy CIMA‐University of Navarra‐IdISNA Pamplona Spain

**Keywords:** adenocarcinoma, biomarker, monocytes, neutrophils, NK cells, Cancer, Immunology

## Abstract

Recent studies highlight the importance of baseline functional immunity for immune checkpoint blockade therapies. High‐dimensional systemic immune profiling is performed in a cohort of non‐small‐cell lung cancer patients undergoing PD‐L1/PD‐1 blockade immunotherapy. Responders show high baseline myeloid phenotypic diversity in peripheral blood. To quantify it, we define a diversity index as a potential biomarker of response. This parameter correlates with elevated activated monocytic cells and decreased granulocytic phenotypes. High‐throughput profiling of soluble factors in plasma identifies fractalkine (FKN), a chemokine involved in immune chemotaxis and adhesion, as a biomarker of response to immunotherapy that also correlates with myeloid cell diversity in human patients and murine models. Secreted FKN inhibits lung adenocarcinoma growth *in vivo* through a prominent contribution of systemic effector NK cells and increased tumor immune infiltration. FKN sensitizes murine lung cancer models refractory to anti‐PD‐1 treatment to immune checkpoint blockade immunotherapy. Importantly, recombinant FKN and tumor‐expressed FKN are efficacious in delaying tumor growth *in vivo* locally and systemically, indicating a potential therapeutic use of FKN in combination with immunotherapy.

## Graphical abstract

Plasma fractalkine level correlates with increased systemic myeloid diversity and clinical response to immunotherapy in lung cancer. NK cell‐mediated tumor growth inhibition *in vivo* indicates therapeutic potential.

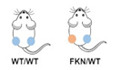

## Introduction

Non‐small‐cell lung cancer (NSCLC) remains a leading cause of cancer death. Immune checkpoint blockade (ICB) immunotherapies such as anti‐PD‐L1/PD‐1 therapies have yielded remarkable clinical results. Nevertheless, these treatments fail in most patients. The mechanisms of resistance to ICB therapies have traditionally been studied within the tumor microenvironment (TME). However, PD‐L1/PD‐1 blocking antibodies are administered systemically, acting over a variety of systemic immune populations that may contribute to clinical outcomes (Bocanegra *et al*, 2019; Rashidian *et al*, 2019; Peng *et al*, 2020). Accumulating evidence is showing that functional systemic immunity in cancer patients before starting immunotherapies is required for ICB success (Mathios *et al*, 2016; Spitzer *et al*, 2017; Rashidian *et al*, 2019; Zuazo *et al*, 2019, 2020; Kagamu *et al*, 2020; Ferrara *et al*, 2021; Horton *et al*, 2021). Indeed, we and others have recently demonstrated that functional systemic T‐cell immunity before ICB is a major requirement for its efficacy (Spitzer *et al*, 2017; Zuazo *et al*, 2019; Kagamu *et al*, 2020; Horton *et al*, 2021). For instance, NSCLC patients with dysfunctional T cells failed to respond to PD‐L1/PD‐1 monotherapies (Zuazo *et al*, 2019).

T‐cell activities are largely regulated through antigen presentation provided by different myeloid cell types. In turn, myeloid cell differentiation and activities are disturbed by cytokines and plasma factors in cancer patients (Robb, 2007). These factors can alter the myeloid cell compartment, and hence disturb T‐cell immunity. For example, tumors secreting different cytokines such as GM‐CSF, G‐CSF, M‐CSF, or IL6 cause systemic expansion of myeloid‐derived suppressor cells (MDSCs) that inhibit anti‐tumor T cells and NK cells (Liechtenstein *et al*, 2014; Gato‐Canas *et al*, 2015; Ortiz‐Espinosa *et al*, 2022). By contrast, elevated numbers of circulating HLA‐DR^+^ monocytes and decreased neutrophils are generally good prognostic factors for ICB therapies (Krieg *et al*, 2018). Therefore, specific cytokine profiles may strongly influence ICB outcomes by altering systemic immunity. Indeed, some of these cytokines have been associated with good prognosis such as IL2, IFNα, or IL12 and have even been used in immunotherapies (Propper & Balkwill, 2022).

A cytokine with ambivalent roles in cancer is fractalkine (FKN), a membrane‐bound chemokine of the CX3C family, which mediates cellular adhesion. This protein is mainly expressed by endothelial cells and signals through its receptor CX3CR1 and integrins (Fujita *et al*, 2012). A soluble form is released by proteolysis mediated by ADAM‐10 and ADAM‐17 (Garton *et al*, 2001; Hundhausen *et al*, 2003), which regulates immune chemotaxis. FKN has been reported to elicit anti‐tumor responses by facilitating immune cell infiltration into tumors (Lavergne *et al*, 2003; Ohta *et al*, 2005; Xin *et al*, 2005; Tang *et al*, 2007; Hyakudomi *et al*, 2008; Park *et al*, 2012; Kee *et al*, 2013; Yamauchi *et al*, 2020, 2021). On the other hand, FKN promotes tumor migration and invasion of circulating tumor cells towards tissues displaying high FKN expression (Korbecki *et al*, 2020) (Shulby *et al*, 2004; Marchesi *et al*, 2008; Erreni *et al*, 2010; Gaudin *et al*, 2011; Jamieson‐Gladney *et al*, 2011; Kim *et al*, 2012; Marchica *et al*, 2019). Thus, FKN plays context‐dependent roles in anti‐tumor immunity, and its role in ICB immunotherapies is still poorly understood.

Here, we performed an extensive profiling of systemic myeloid cell populations and plasma soluble factors in NSCLC patients undergoing PD‐L1/PD‐1 blockade therapies, before and during treatment. Responder patients showed an elevated diversity of myeloid cell types, enriched in activated monocytic cells, and decreased granulocytic populations. Plasma FKN concentration correlated with myeloid cell diversity and response to PD‐L1/PD‐1 blockade. The mechanisms underlying its implication in PD‐L1/PD‐1 blockade were explored both *in vitro* and *in vivo*, as well as its potential use as a therapeutic approach in NSCLC.

## Results

### Baseline plasma FKN is associated with myeloid diversity and objective clinical responses

An extensive characterization of circulating immune cell populations was performed by high‐dimensional flow cytometry (HDFC) (Qiu *et al*, 2011; Krieg *et al*, 2018; Kagamu *et al*, 2020) in a well‐characterized cohort of 112 NSCLC patients (Table EV1A–D) undergoing PD‐L1/PD‐1 blockade immunotherapy. A panel of 43 lineage, differentiation, and activation markers was used to label freshly isolated peripheral blood mononuclear cells (PBMCs) before the initiation of immunotherapies (baseline) and after the first cycle of treatment. Samples from age‐matched healthy donors were used as controls. Patient groups were classified into long‐term responders, stable disease, and progressors, with some patients classified as hyperprogressors in our previous studies (Zuazo *et al*, 2019; Arasanz *et al*, 2020).

**Figure 1:**
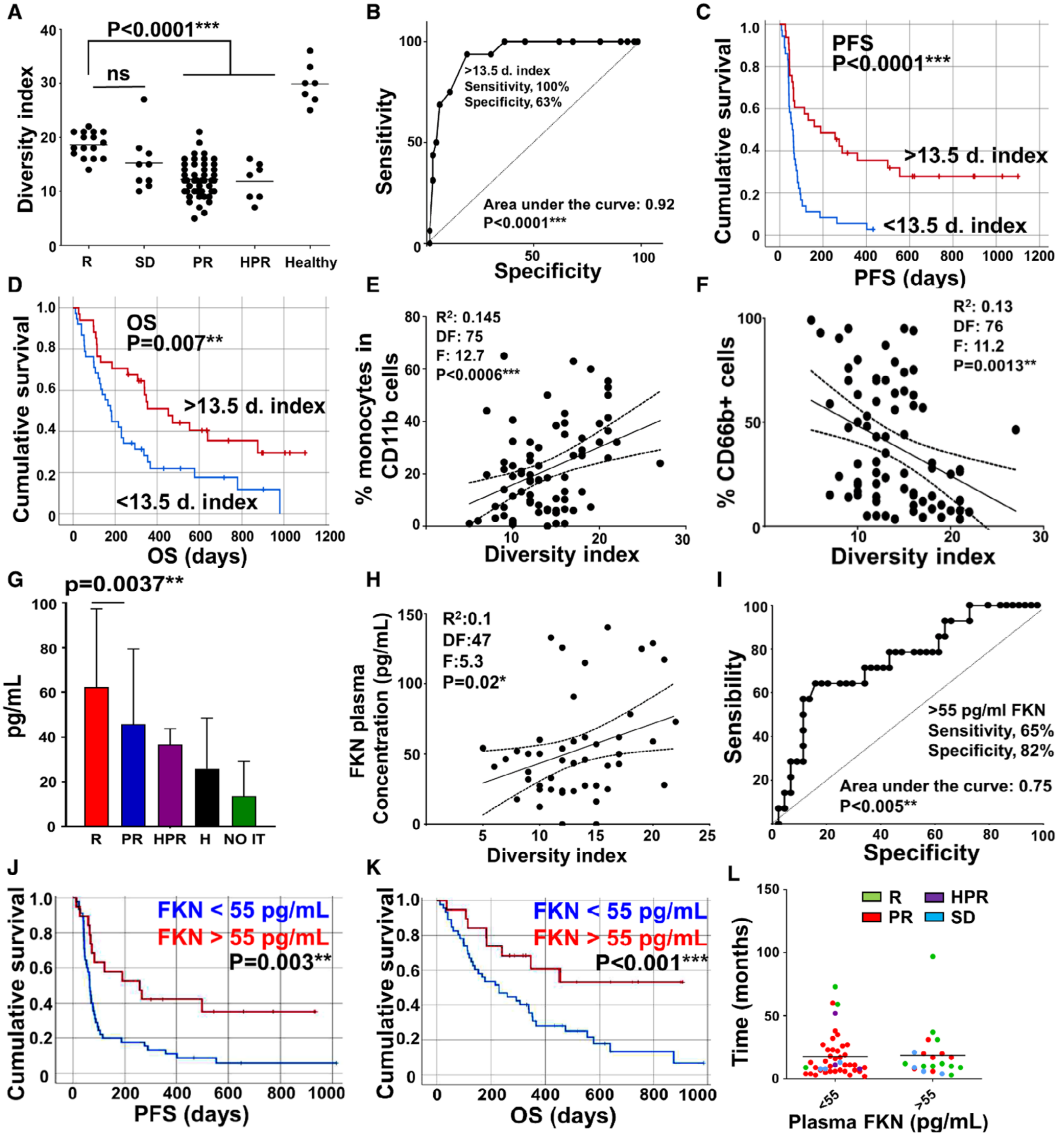
Baseline diversity of myeloid cells and plasma FKN concentrations correlate with clinical responses in NSCLC patients ADiversity indexes as a function of clinical responses. Each dot represents a biological replicate.BROC curve of the diversity index as a function of objective responses vs disease progression.CKaplan–Meier plot of PFS stratifying the patients according to high or low diversity index. *N* = 69 (33 above the cut‐off, 36 below).DAs in (C) but plotting OS. *N* = 72 (34 above the cut‐off, 38 below).ECorrelation between the percentage of monocytes and diversity index. Each dot represents a biological replicate.FCorrelation between the percentage of granulocytic myeloid cells and diversity index. Each dot represents a biological replicate.GBaseline plasma FKN concentrations in responders (R, *n* = 26), progressors (PR, *n* = 62), hyperprogressors (HPR, *n* = 3), age‐matched healthy donors (H, *n* = 32), and patients who were not eligible for immunotherapy (NO IT, *n* = 28). Sample sizes represent biological replicates. Error bars are shown (standard deviations, SD), and relevant statistical comparisons by Mann–Whitney *U* tests are shown in the graphs.HCorrelation between FKN plasma concentration and diversity index with the Spearman's test. Each dot represents a biological replicate.IROC analysis of FKN concentration as a predictor of objective clinical responses. The calculated cut‐off value for the statistics provided in the graph is shown.JKaplan–Meier plot of PFS stratifying the patients according to the FKN cut‐off value identified in the ROC curve (*n* = 42). The associated *P*‐value is shown.KAs in (J) but plotting OS. *n* = 46 (17 above the cut‐off, 29 below).LTime elapsed from disease diagnosis to the beginning of immunotherapy in second‐line treated patients. Each dot represents a biological replicate. Data information: Multicomparisons in dot plots were carried out by the Wilcoxon test. Pairwise comparisons were performed by the Mann–Whitney's *U* test. Survival differences were tested with the log‐rank test and correlation plots with the Spearman's test. *, **, ***, indicate significant (*P* < 0.05), very significant (*P* < 0.01) and highly significant (*P* < 0.001) differences. ns, nonsignificant differences.

**Figure EV1:**
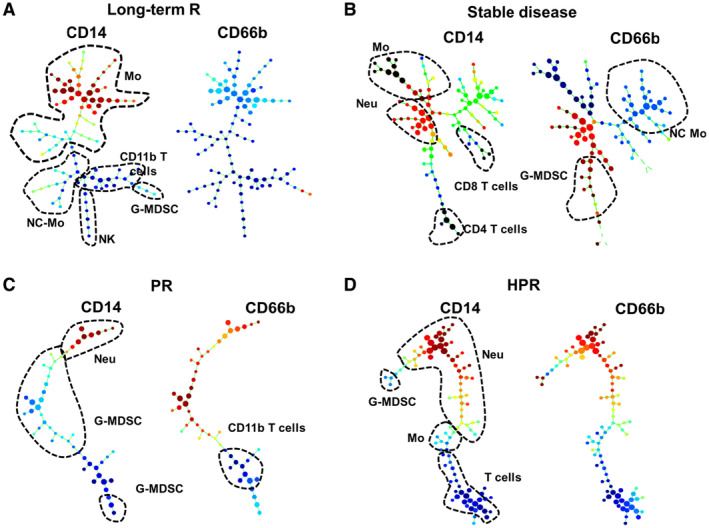
Hierarchical phenotype clustering from high‐dimensional flow cytometry data of baseline multiple cell lineage and activation markers within immune cell subsets in PBMCs from NSCLC patients undergoing anti‐PD‐1/PD‐L1 immunotherapy A–DRepresentative SPADE3 cluster profiles of myeloid cells integrating 43 markers are shown for (A) a long‐term responder, (B) a short‐term responder (stable disease), (C) a progressor (PR), and (D) a hyperprogressor (HPR). Distributions of CD14 (left dendrogram) and CD66b expression (right dendrogram) are shown. Main cell subsets are encircled and identified as Mo—monocytes; Neu—neutrophils; G‐MDSC—granulocytic myeloid‐derived suppressor cells; NC‐Mo—nonclassical monocytes; NK—natural killer cells. The relative expression of the selected marker as indicated above the graphs color‐coded, from dark red (maximum expression) to dark blue (minimum expression).

High‐dimensional hierarchical clustering was performed over the phenotypes of baseline CD11b^+^ myeloid immune populations. Each response group was characterized by distinct cluster profiles (Fig EV1). Responders showed high phenotypic diversity with a dominance of the monocytic lineage, in contrast to nonresponders who exhibited elevated percentages of neutrophils and granulocytic myeloid‐derived suppressor cells (G)‐MDSCs (Fig EV1A and C). A diversity index (DI) was calculated for each patient as the number of terminal phenotype clusters in HDFC profiles generated with 200,000 CD11b^+^ cells. Objective responders exhibited increased DI (*P* < 0.0001) compared with nonresponders before starting immunotherapy (DI = 18.63 ± 2.36 vs. 12.21 ± 3.31; Mean ± SD), although still inferior to that of healthy donors (DI = 29.86 ± 3.71; Mean ± SD) (Fig 1A). Elevated diversity indexes were significantly associated with objective clinical responses as evaluated by ROC analysis (area under the curve, AUC, 0.9216 [95% confidence interval = 0.8601 to 0.9832]; *P* < 0.0001; Fig 1B). A DI cut‐off value of 13.5 identified responders with 100% sensitivity and 63% specificity, which stratified patients with significantly (*P* < 0.0001) increased progression‐free survival (PFS) (Fig 1C) and overall survival (OS; *P* = 0.007; Fig 1D).

**Figure EV2:**
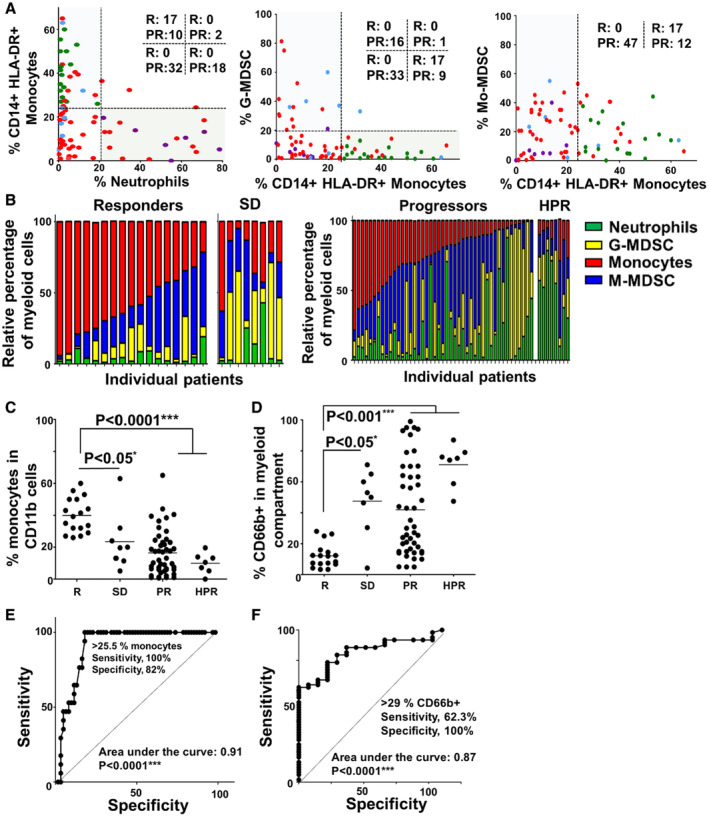
Baseline monocyte/neutrophil profiles in peripheral blood from NSCLC patients undergoing PD‐L1/PD‐1 blockade ALeft graph, baseline frequency of monocytes (CD14^+^ HLA‐DR^+^) vs neutrophils (CD14^+^ CD66b^+^) within CD11b^+^ cells in patients classified as responders (R, green), progressors (PR, red), stable disease (blue) and hyperprogressors (purple). The number of responders and progressors is indicated in each quadrant. Center graph, as in left but plotting the percentage of G‐MDSCs (CD14^−^ CD66b^+^) vs monocytes. Right graph, as in left but plotting the percentage of Mo‐MDSC (CD14^+^ HLA‐DR^−^) vs. monocytes.BLeft graph, relative percentages of the main myeloid populations restricted to this compartment are plotted for each patient under study as indicated as color codes, classified according to objective responders and stable disease (SD); Right graph, as in left but in progressors and hyperprogressors (HPR).CPercentage of circulating monocytes (CD11b^+^ CD14^+^ HLA‐DR^+^) within each response group as indicated.DPercentage of granulocytic cells (CD11b^+^ CD66b^+^) within each response group as indicated.EROC analysis of the percentage of monocytes as a predictor of objective responses.FROC analysis of the percentage of granulocytic myeloid cells as a predictor of no objective response. Data information: R—objective responders (*n* = 16); SD—stable disease (*n* = 9); PR—progressors (*n* = 47); HPR—hyperprogressors (*n* = 7); ns—nonstatistical differences. Relevant statistical comparisons are shown in the graphs. Multicomparisons in dot plots were carried out by the Wilcoxon test. Pairwise comparisons were performed by the Mann–Whitney *U* test. *, ***, ****, indicate significant (*P* < 0.05), highly significant (*P* < 0.001), and very highly significant (*P* < 0.0001) differences.

Patients classified according to clinical responses showed differential myeloid profiles (Fig EV2A and B). Objective responders presented a myeloid signature dominated by monocytic CD14^+^ HLA‐DR^+^ cells and a low abundance of granulocytic CD66b^+^ myeloid cells (neutrophils and G‐MDSCs; Fig EV2C and D). Relative percentages of these myeloid lineages were significant predictors of responses by ROC analyses (Fig EV2E and F). Then, we tested whether DI correlated with relative percentages of monocytes and granulocytes. Albeit data dispersion expected from the inherent heterogeneity of clinical samples, elevated DI was a positive correlator with increased percentages of CD14^+^ HLA‐DR^+^ monocytes within CD11b^+^ cells (Fig 1E), and a negative correlator with increased percentages of systemic CD66b^+^ granulocytic cells (Fig 1F).

**Figure EV3:**
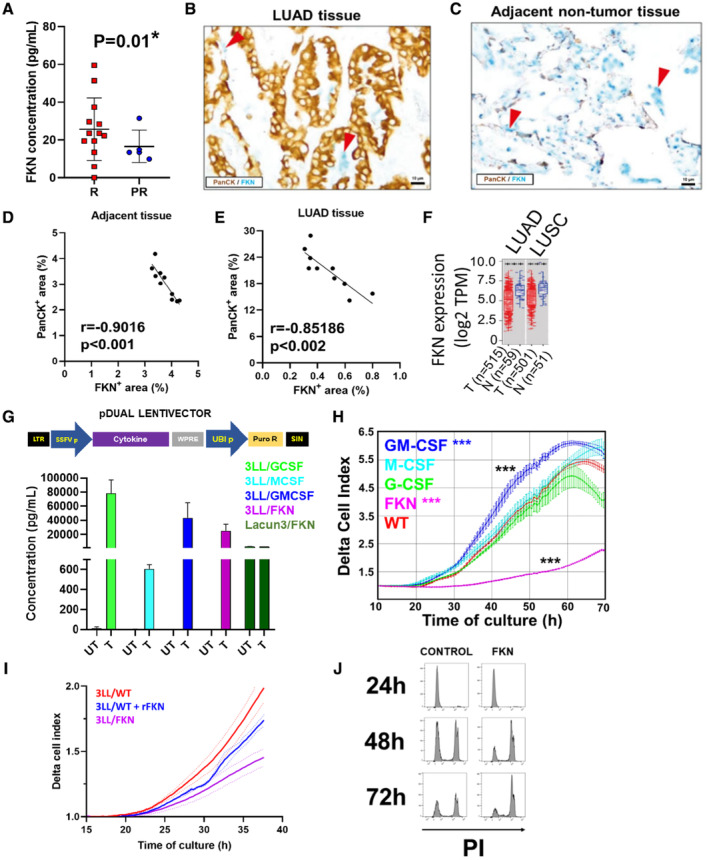
FKN expression in lung cancer patients and engineering of mouse lung cancer cell lines overexpressing myeloid‐regulating cytokines ABaseline plasma FKN concentrations in responders (R, *n* = 13) and progressors (PR, *n* = 5) in a validation cohort of NSCLC patients treated with anti‐PD‐1/PD‐L1 immunotherapy as a first‐line treatment. Error bars are shown (standard deviations, SD). Statistical significance was tested by the chi‐square test.B, C(B) Representative double lung immunostainings for Pan‐Cytokeratin (PanCK) (brown) and FKN (blue; red arrows indicate FKN positive cells) in lung tissue sections from LUAD patients and (C) adjacent healthy tissue.D, E(D) Pearson's correlation of PanCK with FKN positive areas (%) in histologies of adjacent nontumor and (E) LUAD tissues. Pearson's correlation coefficients are shown in the graphs. *n* = 10.FFKN mRNA expression level in tumor (T) and normal tissue (N) of clinical samples from lung adenocarcinoma (LUAD) and squamous lung carcinoma (LUSC) patients registered in the TCGA database. Distributions of gene expression levels are displayed using box plots. Box and whisker plots indicate median (central line), 25^th^ to 75^th^ percentiles (box), and minimum to maximum values (whiskers).GTop, lentivectors for the expression of cytokines of interest. SIN, self‐inactivating deleted LTR; LTR, long‐terminal repeat; SFFVp, spleen focus‐forming virus promoter; UBIp, human ubiquitin promoter; Puro R, puromycin resistance gene. Down, ELISA quantification of cytokine secretion by the indicated engineered lung cancer cell lines expressing the indicated cytokines (T). Endogenous secretion of each cytokine was also quantified in supernatants from cultures of parental unmodified controls (UT). Data are presented as mean ± SD (*n* = 3 independent biological replicates).HReal‐time cell growth (RTCA) of 3LL cell lines engineered to secrete the indicated cytokines. Relevant statistical comparisons of delta‐cell indexes after 50 h of culture were carried out by ANOVA. Data are presented as mean ± SD (*n* = 3 independent biological replicates).IReal‐time cell growth (RTCA) of unmodified and FKN‐producing 3LL cell lines cultured with 1 μg/ml of recombinant FKN (rFKN).JFlow cytometric assessment of propidium iodide (PI) uptake by apoptotic unmodified 3LL cells cultured in 3LL‐WT (control) or 3LL‐FKN (FKN) conditioned medium for 24, 48, or 72 h. Data information: The statistical significance computed by the Wilcoxon test is annotated by the number of stars. *, **, *** indicate significant (*P* < 0.05), very significant (*P* < 0.01) and highly significant (*P* < 0.001) differences.

In light of these results, we wondered whether plasma factors correlating with myeloid function and clinical responses could be identified in our cohort. Therefore, a panel of 65 cytokines, chemokines, and soluble immune checkpoints was evaluated. While most of the quantified analytes showed increased levels in patients who rendered no objective clinical responses to anti‐PD‐1/PD‐L1 immunotherapy (Table EV2), only FKN concentrations were significantly (*P* = 0.0037) elevated in a sample of responders before treatment (Fig 1G; Table EV2). A validation cohort independently confirmed our results in a limited sample of patients (Fig EV3A). FKN plasma concentrations in clinical samples positively correlated with myeloid diversity in our discovery cohort (Fig 1H). A cut‐off value for FKN concentration above 55 pg/ml was identified by ROC analysis (Fig 1I) for significant benefit in PFS (*P* = 0.003) and OS (*P* < 0.001; Fig 1J and K). To find out whether FKN had prognostic value in our cohort for treatments other than immunotherapies, we analyzed the time elapsed between diagnosis and progression in our discovery cohort during which patients were undergoing chemotherapy only. No significant differences were found, thus discarding that plasma FKN had significant prognostic value in our cohort (Fig 1L).

### Secreted FKN possesses potent anti‐oncogenic and immune‐modulating activities

The better therapeutic outcome of patients with increased plasma FKN concentrations suggested that FKN could possess potential anti‐oncogenic properties in lung cancer. Therefore, we decided to assess its potential use as a therapeutic agent.

**Figure 2:**
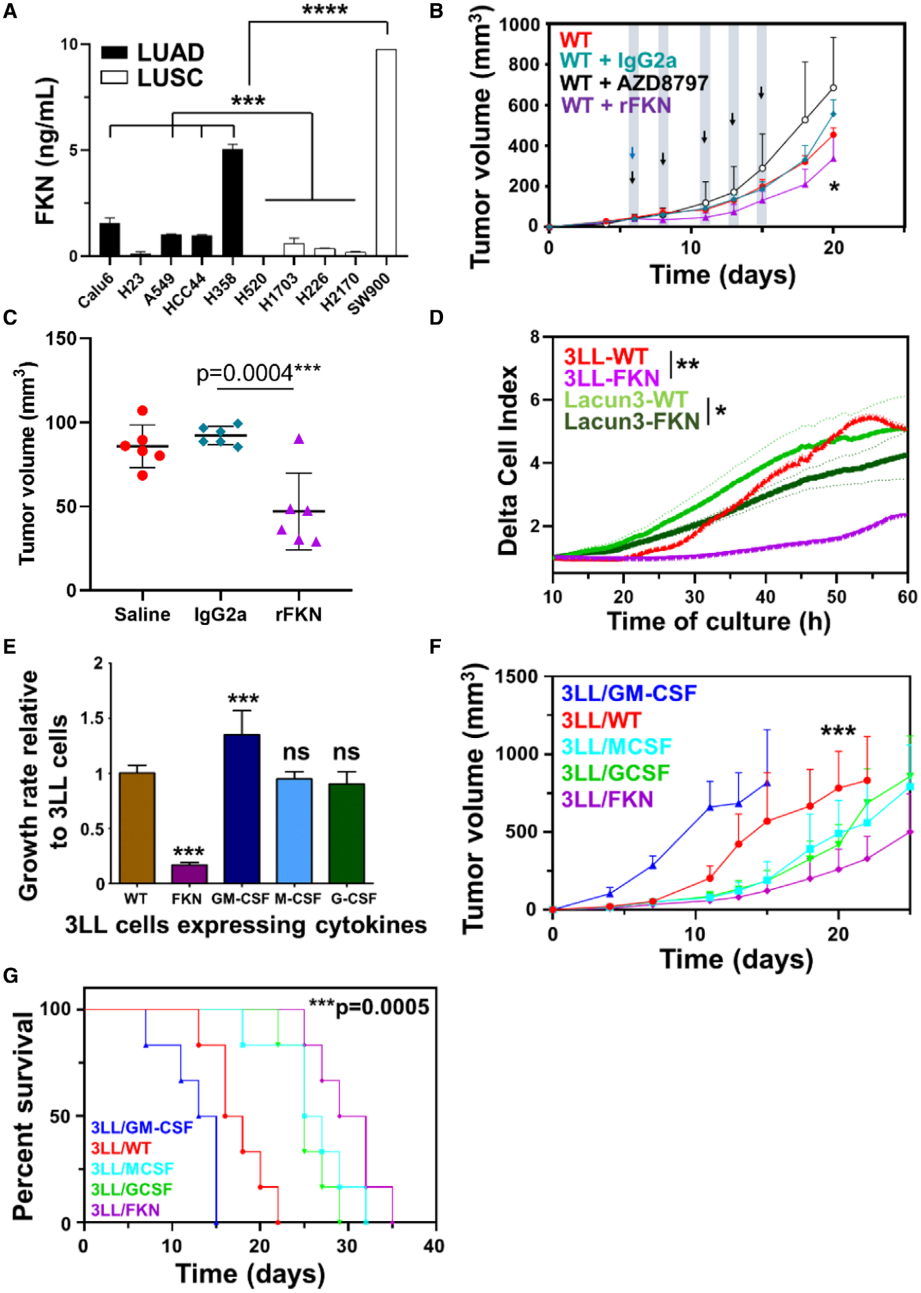
FKN inhibits lung adenocarcinoma cell growth ASecreted FKN in cell cultures of a collection of 10 LUAD and LUSC cancer cell lines quantified by ELISA. Results are presented as mean ± SD. Samples were assayed in duplicates from a pool of three independent replicates. Statistical differences among groups were analyzed by ANOVA followed by Tukey's tests.BTumor growth curves of 3LL tumor‐bearing mice treated with recombinant FKN (rFKN), the CX3CR1 inhibitor AZD8797, a nonrelevant protein (IgG2a) or saline buffer as vehicle control (WT). Arrows indicate the time of intraperitoneal injections of rFKN (blue) and AZD8797 (black). Data are expressed as mean ± SD (*n* = 6 mice per group). Statistical comparisons by ANOVA followed by Tukey's pairwise comparison tests are provided.CDot plot of 3LL tumor size in the indicated groups of mice at day 10 after cancer cell inoculation. Statistical comparisons by ANOVA and Tukey's pairwise comparison tests are indicated.DProliferation of two LUAD murine cell lines overexpressing FKN and their parental unmodified counterparts (WT), as indicated. Real‐time cell growth was monitored by RTCA. Delta‐cell indexes are expressed as mean ± SD from three independent replicates. Differences in delta‐cell index proliferation data were tested by ANOVA following Tukey's pairwise comparison tests after 40 h of culture.EBar graphs with cell growth rates for the indicated 3LL cell lines relative to the growth of unmodified 3LL cells. Error bars are shown (SD). Statistical comparisons among three independent biological replicates by ANOVA and Tukey's pairwise comparison tests are indicated.F
*In vivo* tumor growth of engrafted 3LL cell lines producing the indicated cytokines. Data are expressed as mean ± SD (*n* = 6 mice per group). Comparisons between groups were performed by ANOVA and Tukey's pairwise comparison tests.GKaplan–Meier survival plots. Differences between the control group (WT) and the FKN group were evaluated by a two‐sided log‐rank test. Data information: Statistical comparisons are shown in the graph. *, **, ***, ****, indicate significant (*P* < 0.05), very significant (*P* < 0.01), highly significant (*P* < 0.001), and very highly significant (*P* < 0.0001) differences; ns, nonsignificant differences.

Fractalkine is known to be produced by healthy tissue. To find out whether lung cancer cells could also be a source of FKN in lung cancer patients, we quantified *in vitro* FKN secretion by a collection of 10 human lung adenocarcinoma (LUAD) and lung squamous carcinoma (LUSC) cell lines. FKN production was very heterogeneous, and only two cell lines out of 10 clearly overexpressed FKN (Fig 2A). This was in agreement with our clinical observations since a minor percentage of NSCLC patients showed plasma FKN concentrations above the cut‐off value (Fig 1I). Due to the unavailability of tumor tissue biopsies from our discovery cohort, we evaluated FKN expression in a limited sample of biopsies from another cohort of LUAD patients. Double immunostainings were performed to detect the lung tumor marker Pan‐Cytokeratin (PanCK) together with FKN in tumor and adjacent healthy tissue (Fig EV3B and C). FKN expression was inversely correlated with PanCK expression within both adjacent nontumor and LUAD tissues (Pearson's correlation: *r* = −0.9016, *P* < 0.001 [adjacent nontumor tissues] and *r* = −0.8518; *P* < 0.002 [LUAD tissues; Fig EV3D and E]). *In vitro* results together with biopsy data were indicators that FKN was poorly expressed in tumor tissue in the majority of lung cancer patients. These data were confirmed by evaluating FKN transcriptomic expression in clinical samples from lung cancer patients registered in The Cancer Genome Atlas (TCGA) database (Fig EV3F).

To find out whether FKN could trigger anti‐tumor mechanisms in NSCLC patients, the FKN‐CX3CR1 signaling axis was systemically targeted in a mouse model of lung adenocarcinoma. In agreement with our hypothesis, the pharmacological inhibition of CX3CR1 by the administration of the allosteric noncompetitive FKN antagonist AZD8797 accelerated tumor progression (*P* < 0.05; Fig 2B). This result was supported by the systemic administration of recombinant FKN, which delayed tumor growth, compared with the null effect of a nonrelevant control protein (IgG) (Fig 2C). To overcome the challenging therapeutic schedule with recombinant FKN in mouse models, we overexpressed FKN directly from cancer cell lines, as observed for some human lung adenocarcinoma cell lines such as H358 and SW900 (Fig 2A). To that end, murine adenocarcinoma cell lines Lacun‐3 (Bleau *et al*, 2014) and 3LL were engineered to express FKN (Fig EV3G). Since a tendency for increased concentrations of hematopoietic growth factors such as GM‐CSF and G‐CSF were identified in the plasma of responder patients (Table EV2), and a correlation between FKN levels and myeloid diversity was also found (Fig 1H), we included cancer cell lines overexpressing key hematopoietic growth factors as controls (Fig EV3G). The two FKN‐expressing LUAD cell lines showed impaired cell growth *in vitro* (Figs 2D and EV3H). Interestingly, GM‐CSF expression from cancer cells significantly enhanced 3LL proliferation (Figs 2E and EV3H). By contrast, FKN was a strong inhibitor of tumor cell growth rate and an inductor of apoptosis of cancer cells (Figs 2E and EV3H–J).

To evaluate the growth dynamics of tumors expressing FKN and control molecules, 3LL cell lines expressing FKN, GM‐CSF, G‐CSF, and M‐CSF were subcutaneously transplanted in groups of mice. FKN secretion strongly impaired (*P* = 0.0002) 3LL tumor growth (Fig 2F) and increased median survival from 17 days in the control group to 30.5 days (*P* = 0.0005; Fig 2G). G‐CSF and M‐CSF expression also delayed tumor growth and increased survival but to a lesser extent. In agreement with *in vitro* data, GM‐CSF expression by 3LL cells significantly accelerated tumor progression. These results demonstrated that FKN exerted anti‐tumor activities directly as a recombinant protein and when expressed by tumor cells.

**Figure 3:**
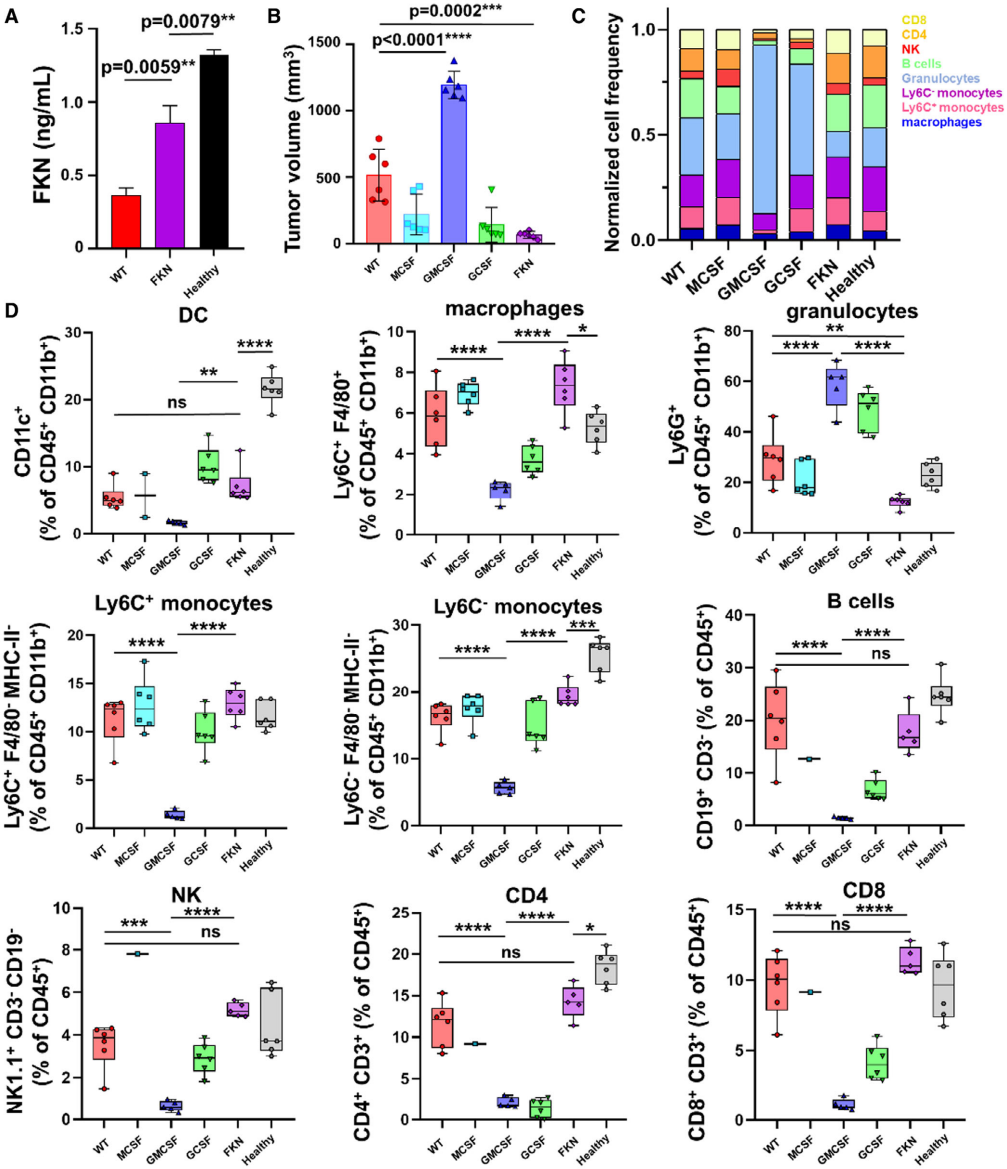
Alteration of immune composition of peripheral blood by tumors expressing cytokines AFKN plasma concentration at day 15 in the indicated groups of tumor‐bearing mice. Data are expressed as mean ± SD from a pool of 6 mice/group. Statistical comparisons were performed by ANOVA and Tukey's pairwise comparison tests.BTumor volumes 15 days after tumor inoculation. Data are expressed as mean ± SD (*n* = 6 mice per group), and comparisons between groups were performed by ANOVA and Tukey's pairwise comparison tests.CRelative myeloid and lymphoid composition of peripheral blood at day 15 in mice transplanted with 3LL‐expressing the indicated cytokines. WT, unmodified 3LL cells; Healthy, mice without tumors.DPercentage of the indicated peripheral immune cell populations at day 15 after injection of groups of mice (*n* = 6 mice per group) with 3LL cells overexpressing the indicated myeloid‐regulating cytokines. The relevant immune populations were quantified as percentages of total leukocytes (CD45^+^ cells), specifying DCs (CD11c), macrophages (F4/80), Ly6C^+^ monocytes, and Ly6C^−^ monocytes, granulocytes (Ly6G), B cells (CD19), NKs (NK1.1), CD4 T cells (CD3 CD4) and CD8 T cells (CD3 CD8). Relevant statistical comparisons were performed by the Wilcoxon's test followed by pairwise comparisons of relevance by the Mann–Whitney *U* test. Box and whisker plots indicate median (central line), 25^th^ to 75^th^ percentiles (box), and minimum to maximum values (whiskers). Data information: *, **, ***, ****, indicate significant (*P* < 0.05), very significant (*P* < 0.01), highly significant (*P* < 0.001), and very highly significant (*P* < 0.0001) differences. In this last case, no exact *P*‐value is provided in the figure; ns, nonsignificant differences.

We then studied whether FKN production by tumors altered the composition of circulating immune cells. First, we confirmed that mice carrying FKN‐producing 3LL tumors had elevated FKN concentrations in plasma compared with mice bearing unmodified 3LL tumors (0.85 ng/ml ± 0.11 vs. 0.36 ng/ml ± 0.05; Means ± SD). Even so, mice with tumors showed a reduction in circulating FKN compared with control mice (1.32 ng/ml ± 0.03) at least at early time points with relatively small tumors (67.47 ± 25.39 mm^3^ in FKN‐producing tumor‐bearing mice vs 516.3 ± 194.1 mm^3^ in wild‐type control group; Fig 3A).

Profiling of circulating immune cells was performed 15 days after tumor engraftment, once significant differences in tumor volume were observed (Fig 3B). In our murine experimental models, the composition of myeloid and lymphoid compartments in tumor‐bearing mice was apparently similar to that of healthy control mice with the exception of GM‐CSF‐ and G‐CSF‐producing tumors (Fig 3C). When looked at in detail and in agreement with our clinical data, mice with 3LL‐FKN tumors had elevated circulating DC, macrophages, and monocytes, accompanied by a significant decrease (*P* < 0.01) in the percentage of Ly6G^+^ granulocytes (12.25% ± 2.36) compared with control mice with 3LL tumors (29.2% ± 9.9; Fig 3D). By contrast, mice bearing 3LL/GM‐CSF tumors showed profiles comparable to that of human hyperprogressors, characterized by an exponential acceleration of tumor growth (*P* < 0.0001) tumor growth, shortened survival (median survival of 14 days vs. 17 days of wild‐type control, *P* = 0.009) and overwhelming numbers of MDSCs (58.52% ± 9.17 of Ly6G^+^ cells).

### FKN sensitizes lung adenocarcinoma tumors intrinsically resistant to anti‐PD‐1 immunotherapies

**Figure 4:**
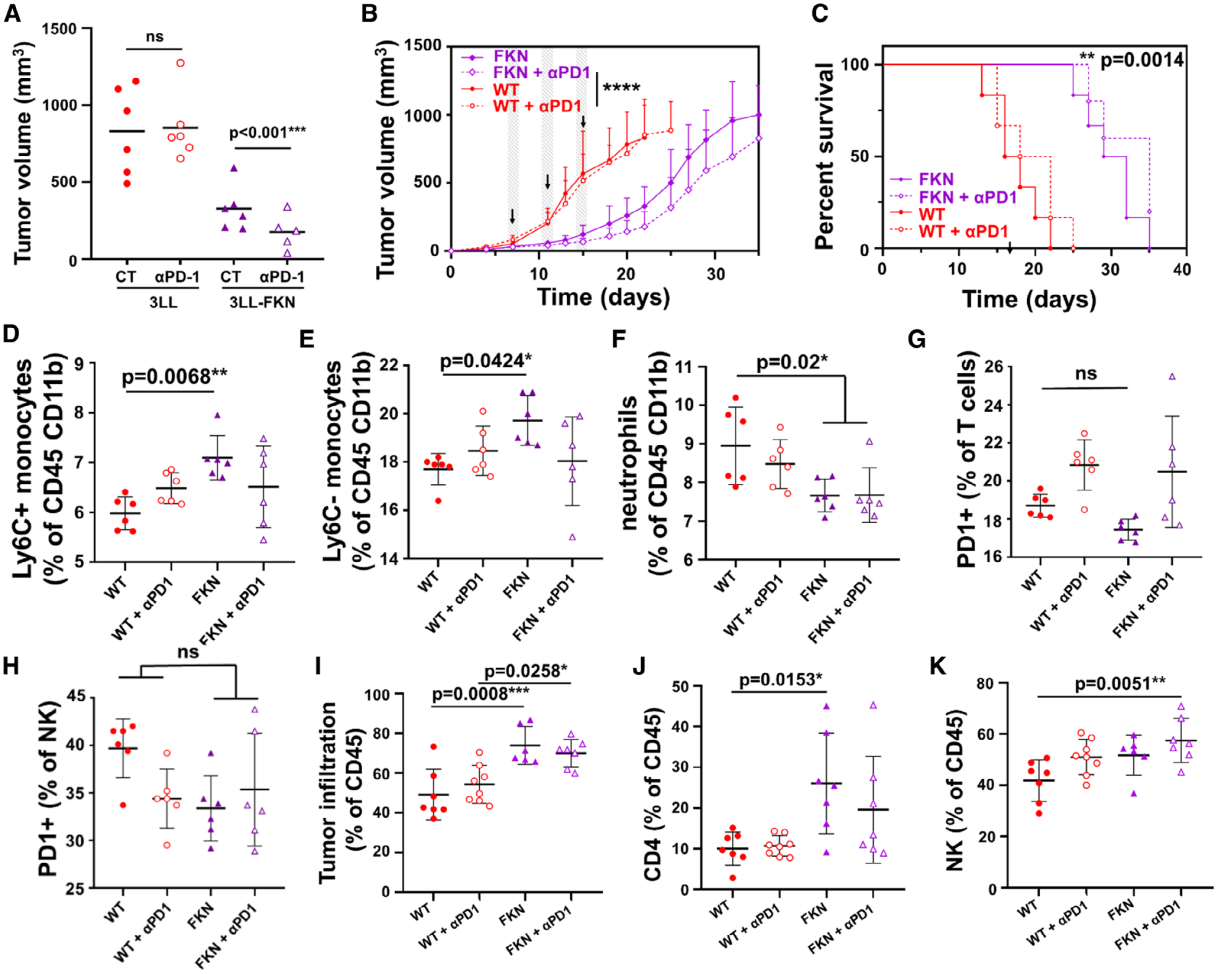
*In vivo* anti‐tumor mechanisms of FKN in combination with PD‐1 blockade in a murine model of lung adenocarcinoma ADot plot of tumor volume in mice 22 days after engraftment of the indicated cancer cell lines and subjected to either a control treatment or anti‐PD‐1 therapy. Relevant statistical comparisons are shown in the graph by the *U* of Mann–Whitney test.BUnmodified and FKN‐expressing 3LL cells were subcutaneously inoculated in mice and tumors were allowed to grow for 7 days. Tumor‐engrafted mice were treated intraperitoneally with anti‐PD‐1 antibody or vehicle (days 7, 11, and 15, as indicated by arrows) following randomization into two groups. Tumor growth was monitored. Data are presented as mean ± SD (*n* = 6 mice per group).CKaplan–Meier survival plots of the indicated groups of mice. Survival differences between the control group (WT + αPD‐1) and FKN + αPD‐1 were evaluated by a two‐sided log‐rank test.D–H(D) Percentage of splenic Ly6C+ monocytes, (E) Ly6G^−^ monocytes, (F) Ly6G^+^ neutrophils, (G) PD‐1^+^ T cells (CD3^+^), and (H) PD‐1^+^ NK cells (NK1.1^+^). Data are presented as mean ± SD (*n* = 6 mice per group).I–K(I) Percentage of tumor infiltration with total leukocytes (CD45^+^), (J) CD4 T cells, and (K) NK cells (NK1.1^+^). Infiltration data are shown as mean ± SD (*n* = 8 mice per group). Data information: Relevant statistical comparisons are shown in the graphs with ANOVA and Tukey's pairwise comparisons. *, **, ***, ****, indicate significant (*P* < 0.05), very significant (*P* < 0.01), highly significant (*P* < 0.001), and very highly significant (*P* < 0.0001) differences; In this last case, no specific *P*‐value is given in the figure; ns, nonsignificant differences.

Our clinical data associated elevated plasma FKN concentrations with objective clinical responses to PD‐L1/PD‐1 blockade. Therefore, we investigated whether FKN contributed to ICB efficacy in the murine 3LL lung adenocarcinoma model, which is intrinsically refractory to these immunotherapies. Indeed, while unmodified 3LL tumors were fully resistant to PD‐1 blockade *in vivo*, FKN expression sensitized tumors to PD‐1 blockade. This sensitization led to a highly significant (*P* < 0.001) reduction in tumor size (Fig 4A) although with limited impact on survival (Fig 4B and C). It has nevertheless to be noted that in our mouse models, FKN expression is a major driving force of anti‐tumor activities, causing a very highly significant delay (*P* < 0.0001) in tumor growth (Fig 4B) associated with a very significant (*P* = 0.001) increase in median survival from 20 to 35 days (Fig 4C).

**Figure EV4:**
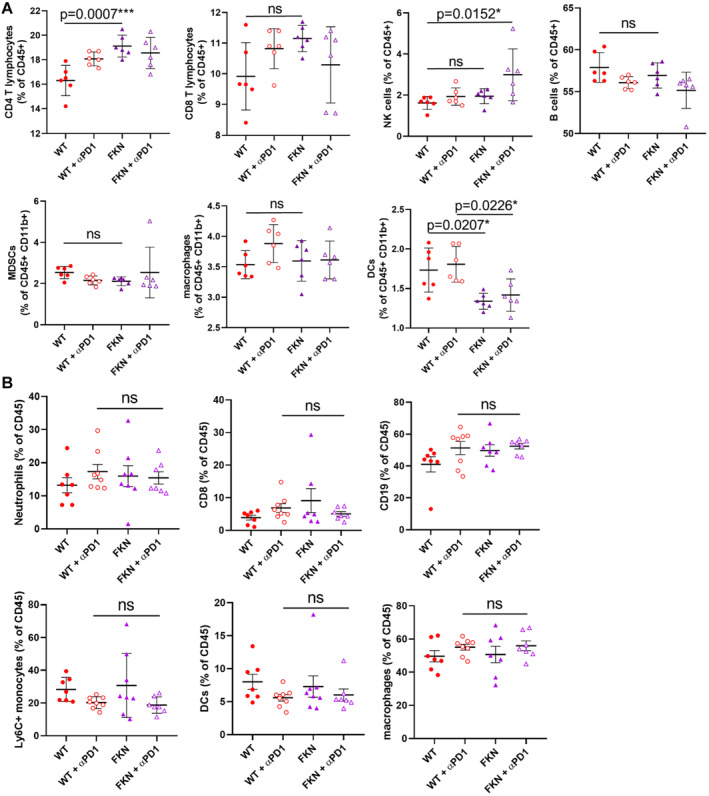
Immune profiling in mice transplanted with 3LL cells expressing FKN and its combination with PD‐1 blockade AThe graphs represent percentages of the indicated infiltrating immune cell types as quantified by flow cytometry, in spleens obtained from mice inoculated with the indicated cell lines (parental cell line, WT; 3LL cells expressing FKN, FKN) with or without PD‐1 blockade treatment. CD4 and CD8 T lymphocytes, NK cells (NK1.1), B cells (CD19), MDSCs (Ly6G^+^ CD115^+^), macrophages (F4/80), and DCs (CD11c) were quantified at day 14 after tumor inoculation. Data are shown as the mean of the percentage within total leukocytes (CD45^+^) ± SD (*n* = 6 mice). Relevant statistical comparisons are shown in the graphs, evaluated by ANOVA and Tukey's pairwise comparisons. *, ***, indicate significant (*P* < 0.05) and highly significant (*P* < 0.001) differences. ns, nonsignificant differences.BGraphs represent percentages of the indicated infiltrating immune cell types as quantified by flow cytometry, in tumors excised from mice inoculated with the indicated cell lines (parental cell line, WT; 3LL cells expressing FKN, FKN) with or without PD‐1 blockade. Neutrophils (Ly6G), CD8, B cells (CD19), Ly6C^+^ monocytes, DCs (CD11c), and macrophages (F4/80) were quantified at day 14 after tumor inoculation. Data are shown as the mean of the percentage within total leukocytes (CD45^+^) ± SD (*n* = 8 mice). Relevant statistical comparisons are shown in the graphs, evaluated by ANOVA and Tukey's pairwise comparisons. ns, nonsignificant differences.

**Figure EV5:**
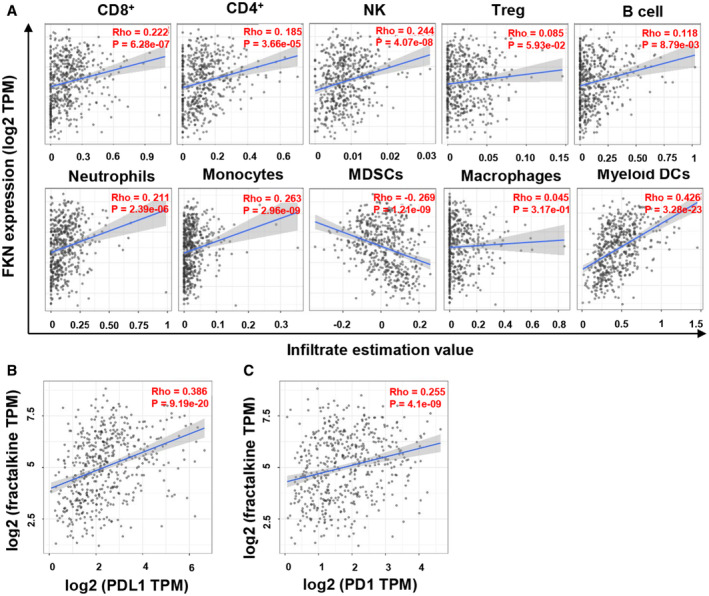
FKN transcriptomic expression in human lung adenocarcinoma samples and correlation with tumor immune infiltration, survival, and PD‐L1 tumor expression AEvaluation of tumor infiltration with the indicated selected immune cell populations, and correlation analyses of FKN transcriptional expression and immune infiltrates from the TCGA database. Analyses were restricted to lung adenocarcinoma samples (*n* = 515). Spearman correlation with different immune populations was identified by several algorithms (CIBERSORT, quanTIseq, xCell, TIDE) and an adjustment based on tumor purity was employed to minimize the potential interaction of low tumor cell quantities. Each dot represents a single tumor sample. Spearman's rho value and *P*‐values are provided within the graphs.BCorrelation between tumor FKN and PD‐L1 transcriptional expression by the Spearman's test. Relevant statistical results are presented within the graphs.CAs in (B) but plotting PD‐1 transcriptional expression levels.

Then, we studied whether the combination of FKN expression and PD‐1 blockade had a further impact on the composition of systemic immune cells. These studies were carried out at a time point with significant differences in tumor volumes (15 days after tumor engraftment). We characterized the relative composition of major immune cell types within CD45^+^ cells in spleens, to detect systemic changes in our mouse models. No major impact of the anti‐PD‐1 immunotherapy was observed on top of FKN at least within myeloid cell types (Fig EV4A). In agreement with our clinical data and previous experiments, FKN expression increased the relative percentage of classical and nonclassical monocytes (Fig 4D and E), and a reduction in neutrophils (Fig 4F). Anti‐PD‐1, however, increased the percentage of PD‐1^+^ T cells (Fig 4G) but not of NK cells (Fig 4H). Then, we evaluated whether these changes had a reflection on tumor infiltration. FKN expression significantly enhanced immune infiltration of the tumor (Fig 4I), mainly with CD4 T and NK cells (Fig 4J and K). No significant differences were observed for the rest of the main cell types (Fig EV4B). As we lacked biopsy data from our cohort of patients, we complemented the results in mouse models with analyses of transcriptional data from lung adenocarcinoma samples in the TCGA database. The Tumor Immune Estimation Resource tool (TIMER2.0) was used to estimate immune infiltration and perform correlations with FKN transcription (Li *et al*, 2020). Positive correlations of FKN expression were found with infiltration by CD8 (Rho = 0.222, *P* = 6.28e‐07), CD4 (Rho = 0.185, *P* = 3.66e‐05), NK (Rho = 0.244, *P* = 4.07e‐08) cells, neutrophils (Rho = 0.211, *P* = 2.39e‐06) and monocytes (Rho = 0.263, *P* = 2.96e‐09) (Fig EV5A). By contrast, a negative correlation with MDSC infiltration was observed (Rho = −0.269, *P* = 1.21e‐09). The correlation of FKN with PD‐L1 tumor expression was also confirmed, as PD‐L1 is a clinically‐approved biomarker of response to PD‐L1/PD‐1 blockade (Doroshow *et al*, 2021) (Fig EV5B). Increased FKN transcription also correlated with PD‐1 expression (Fig EV5C).

**Figure 5:**
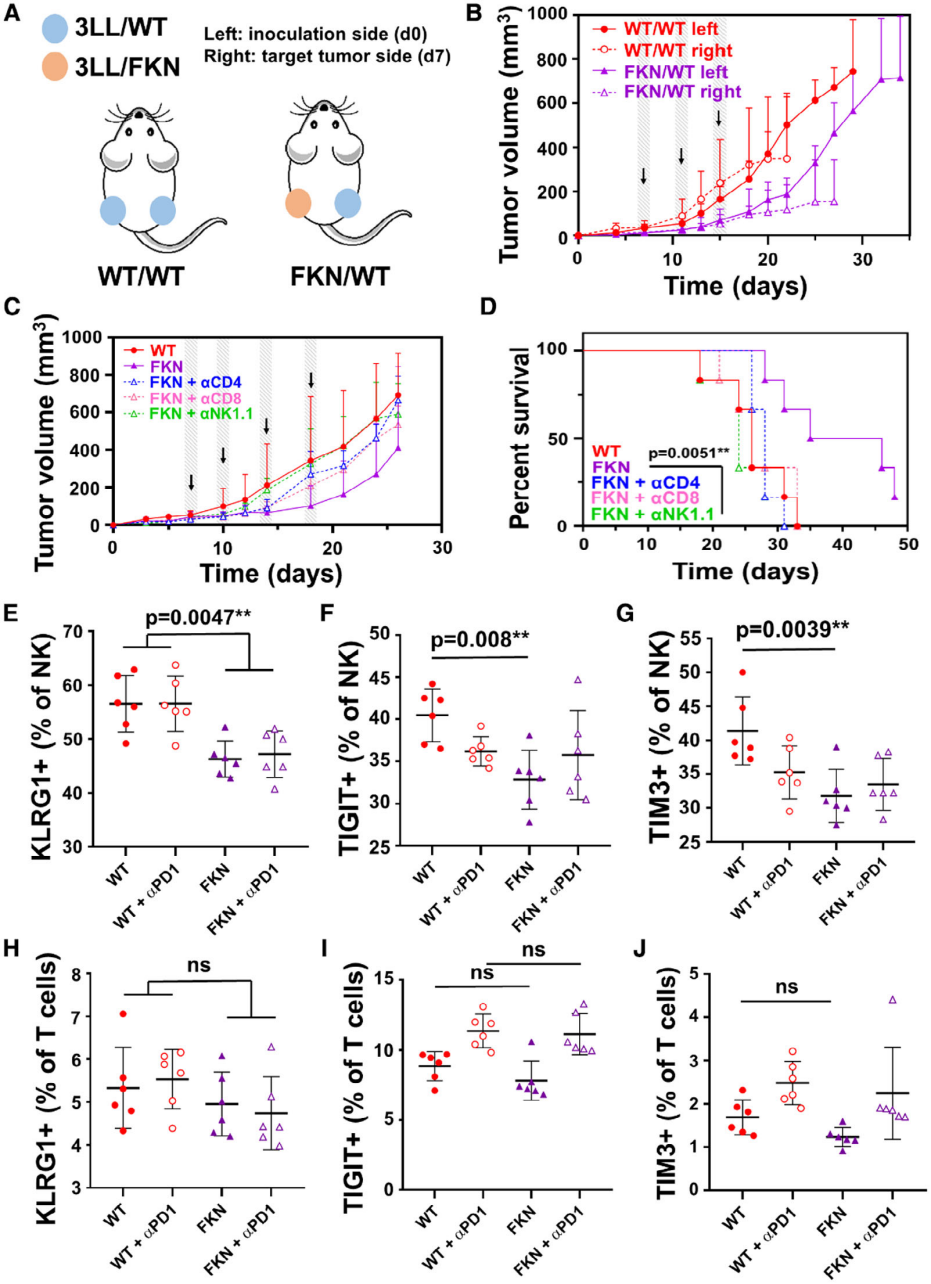
Systemic anti‐tumor mechanisms of FKN in a murine model of lung adenocarcinoma AExperimental schedule for testing systemic FKN anti‐tumor activities. 3LL‐parental (WT) or 3LL‐FKN (FKN) cells were subcutaneously injected into the left flank of mice (inoculation side). Seven days later, 3LL‐parental cells were engrafted on the right flank (target tumor side). Mice were intraperitoneally treated with anti‐PD‐1 antibody at days 7, 11, and 15 after the last tumor inoculation.BTumor growth of right and left flank engrafted tumors in the experimental schedule shown in (A). Data are presented as mean ± SD (*n* = 6 mice per group).CTumor growth in mice intraperitoneally treated with anti‐CD4, anti‐CD8, and anti‐NK1.1 depleting antibodies at days 6, 10, 14, and 18 after tumor inoculation, as indicated by arrows. Data are presented as mean ± SD (*n* = 6 mice per group).DKaplan–Meier survival plot of the indicated treatment groups. Survival differences between the control group (WT) and FKN were evaluated by a two‐sided log‐rank test.E–J(E) Percentage of splenic NKs (NK1.1^+^) expressing KLRG1, (F) TIGIT, and (G) TIM3. (H) Percentage of splenic T cells (CD3^+^) expressing KLRG1, (I) TIGIT, and (J) TIM3. Data are presented as mean ± SD (*n* = 6 mice per group). Comparisons among groups were performed by ANOVA and Tukey's pairwise comparison tests. Data information: ** indicate very significant (*P* < 0.01) differences; ns, nonsignificant differences.

Most of the experiments in mouse models were carried out by expressing FKN from cancer cells. It could be argued that anti‐tumor effects were restricted to the FKN‐expressing tumors, while the clinical data (Fig 1G) and treatments with recombinant FKN (Fig 2B and C) strongly suggested systemic anti‐tumor activities by circulating FKN. Therefore, to demonstrate systemic activities for secreted FKN, 3LL‐FKN cells were subcutaneously implanted in mice on the left flank (inoculation side), followed by injection of unmodified 3LL cells on the right flank (target tumor side) 1 week later (Fig 5A). Mice were then treated with anti‐PD‐1 immunotherapy and tumor growth was monitored at both flanks. Interestingly, the growth of 3LL tumors was delayed and comparable to 3LL‐FKN tumors in the same mice (Fig 5B). These results demonstrated that secreted FKN associated with anti‐PD‐1 blockade could delay the growth of an immunotherapy‐resistant distal tumor that did not express FKN. Therefore, FKN secreted from modified tumor cells showed efficacious systemic therapeutic activities.

To identify the anti‐tumor effector cells in our experimental systems, CD4, CD8, and NK cells depletions were carried out following standard procedures in our group (Ortiz‐Espinosa *et al*, 2022), and the growth of 3LL‐FKN tumors was monitored. Interestingly, NK cell abrogation restored the growth of 3LL‐FKN tumors to rates comparable to unmodified 3LL tumors (Fig 5C). Limited effects were observed by CD4 and CD8 T‐cell depletion. These results suggested that the main effector immune cell types were NK cells with a minor contribution by T cells. NK depletion shortened median mice survival down to 24 days, compared with 40.5 days of median survival in the FKN control group (*P* = 0.0051; Fig 5D). We hypothesized that FKN and anti‐PD‐1 could be systemically reducing the percentage of NK cells expressing inhibitory checkpoints and exhaustion markers other than PD‐1. Indeed, a significant decrease in KLRG1^+^, TIGIT^+^, and TIM3^+^ NK cells was observed (Fig 5E–G). No significant differences were observed in T cells, in agreement with our depletion data (Fig 5H–J).

## Discussion

Increasing evidence indicates that functional systemic immunity is required for the success of immunotherapies (Mathios *et al*, 2016; Spitzer *et al*, 2017; Bocanegra *et al*, 2019; Rashidian *et al*, 2019; Zuazo *et al*, 2019; Arasanz *et al*, 2020; Kagamu *et al*, 2020; Ferrara *et al*, 2021; Horton *et al*, 2021). For example, dysfunctional peripheral blood CD4 and CD8 T cells prior to immunotherapies can be used as biomarkers of clinical failure in PD‐L1/PD‐1 monotherapies, as shown by us and others (Spitzer *et al*, 2017; Zuazo *et al*, 2019; Kagamu *et al*, 2020; Ferrara *et al*, 2021). Likewise, systemic expansion of myeloid suppressor cells and neutrophils constitute poor prognostic markers not restricted to immunotherapies (Kargl *et al*, 2019; Koh *et al*, 2020; Veglia *et al*, 2021). MDSCs and granulocytes are strong T‐cell suppressors in cancer through several mechanisms, as extensively reviewed elsewhere (Veglia *et al*, 2018; Grover *et al*, 2021). On the other hand, elevated percentages of HLA‐DR^+^ monocytes correlate with efficacious PD‐1 blockade immunotherapy (Krieg *et al*, 2018). Hence, profiling of immune cell composition and functionality in peripheral blood could be used for the selection of potential responder patients in PD‐L1/PD‐1 blockade immunotherapies. Following this reasoning, we performed detailed analyses of myeloid cell subsets and their differentiation stages in a discovery cohort of NSCLC patients before starting immunotherapies by HDFC. Here, we confirmed that an elevated diversity of circulating myeloid cell types was characteristic of objective responders to PD‐L1/PD‐1 blockade. It could be argued that a diverse composition of peripheral immune cell phenotypes could reflect functional myelopoiesis (Schultze *et al*, 2019) in patients before starting ICB. Indeed, the elevated diversity of phenotypes was represented by an expansion of activated classical monocytes with a concomitant decrease in granulocytic myeloid cells. Our data confirmed that in addition to their classical prognostic value, these myeloid cell types show significant predictive power in PD‐L1/PD‐1 blockade immunotherapies by ROC analyses, in agreement with previous studies with high‐dimensional analytical techniques (Gubin *et al*, 2018; Krieg *et al*, 2018; Arasanz *et al*, 2022). Our high‐dimensional profiles included (but were not restricted to) DC, monocytes, macrophages, neutrophils, and MDSCs at various differentiation and activation stages, and demonstrated that the balance between these immune cell types is critical for ICB outcome before starting immunotherapies.

On the lookout for plasma factors associated with (i) high systemic myeloid cell diversity, (ii) elevated monocyte populations, and (iii) increased survival, we found that only FKN fulfilled these three conditions. Hence, in our discovery cohort FKN plasma concentration showed good predictive value, which was confirmed in a validation cohort. In agreement with the clinical data, mice engrafted with FKN‐producing tumors showed increased OS with elevated percentages of monocytes and reduced Ly6G^+^ granulocytes in peripheral blood and spleen.

Fractalkine was demonstrated to have anti‐cancer properties both *in vitro* and in *in vivo* models for murine lung adenocarcinoma. Therapeutic activities were observed for injected recombinant FKN or produced by 3LL cells. Indeed, systemic pharmacological disruption of the FKN‐receptor signaling axis accelerated the growth of adenocarcinoma tumors. Our results showed that local FKN expression by the tumor boosted stronger anti‐tumor immune responses that resulted in faster tumor rejection.

It is important to note that FKN possessed systemic anti‐tumor activities without the need to be expressed within the tumor microenvironment. For example, FKN expressed by a tumor can exert inhibitory activities over a distal tumor that does not express FKN. This is important because it was previously thought that FKN expressed by tumors could exert anti‐tumor effects through the recruitment of T cells, DCs, and NK cells to the tumor microenvironment (Lavergne *et al*, 2003; Ohta *et al*, 2005; Xin *et al*, 2005; Tang *et al*, 2007; Hyakudomi *et al*, 2008; Park *et al*, 2012; Kee *et al*, 2013; Yamauchi *et al*, 2020, 2021). Nevertheless, we found that recombinant FKN was very unstable *in vivo*. The systemic administration of recombinant FKN as a therapeutic approach might require experimental strategies to effectively deliver cytokines to the TME in a targeted way while improving their stability.

Anti‐tumor systemic activities were mediated mainly by circulating NK cells, with a minor contribution by CD4 and CD8 T cells. It has been shown in some tumor types that the membrane‐bound FKN can promote metastasis of CX3CR1^+^ circulating tumor cells towards tissues displaying a high CX3CL1 expression, such as bones, lungs, and nervous tissues (Shulby *et al*, 2004; Marchesi *et al*, 2008; Erreni *et al*, 2010; Gaudin *et al*, 2011; Jamieson‐Gladney *et al*, 2011; Kim *et al*, 2012; Marchica *et al*, 2019; Korbecki *et al*, 2020). Our data clearly showed that at least in LUAD cancer models this was not the case. This dual behavior of FKN may probably be a consequence of the two different bioactive forms of the protein (Vitale *et al*, 2007; D'Haese *et al*, 2010; Winter *et al*, 2020). The membrane‐bound form mediates cell adhesion processes and migration while the soluble version participates in the chemoattraction of effector cells to the tumor, but also in the modulation of systemic NK cells, which show less exhausted phenotypes.

Importantly, we demonstrated that FKN expression in adenocarcinoma cells refractory to ICB therapy (Ajona *et al*, 2020) could sensitize tumors to PD‐1 blockade. This sensitization further retarded the growth of tumors, although in our mouse models, this did not translate into a benefit in survival. Nevertheless, it has to be mentioned that FKN exerted very potent anti‐tumor activities, and improvements in survival would likely require blockers even stronger than classical anti‐PD‐1 antibodies.

Concluding, plasma FKN is a biomarker of systemic myeloid cell diversity, characterized by an elevation in circulating monocytic myeloid cells and a strong decrease in granulocytes. FKN plasma concentrations also correlate with increased survival in human lung adenocarcinoma patients treated with PD‐L1/PD‐1 blockade monotherapies. Our results demonstrate that the therapeutic activities of FKN are not restricted to the tumor microenvironment. These results open an avenue towards either utilizing FKN concentration as a biomarker or its development as a therapeutic agent.

## Materials and Methods

### Clinical samples and study design

One hundred and twelve patients diagnosed with locally advanced or metastatic NSCLC treated with the immune checkpoint inhibitors (ICI) nivolumab, pembrolizumab, and atezolizumab, or with the combination of chemo‐immunotherapy (pembrolizumab + platinum‐based chemotherapy) were recruited between December 2017 and October 2020 at the Oncology Department of the University Hospital of Navarra (Pamplona, Spain) (Table EV1). Hyperprogressive disease was defined by radiological criteria and immunological profiles as described in Arasanz *et al* (2020). The exclusion criteria consisted of concomitant administration of chemotherapy or previous immunotherapy treatment.

The current prospective observational study was approved by the Ethics Committee of Clinical Investigations at the University Hospital of Navarre (reference number: PI_2020/115). REMARK reporting guidelines were followed for the study. Informed consent was obtained from all subjects and all experiments conformed to the principles set out in the WMA Declaration of Helsinki and the Department of Health and Human Services Belmont Report. Samples were collected through the Blood and Tissue Bank of Navarre, Health Department of Navarre, Spain. Eligible patients were 18 years of age or older who received immunotherapy targeting PD‐1/PD‐L1 as their current standard of care. Tumor PD‐L1 expression was quantified in 103 of these patients. Thirty‐two age‐matched healthy donors were recruited from the Valle de Salazar Nursing Home (Navarra, Spain) from whom written informed consents were obtained. The total number of donors was calculated *a priori* to ensure a power of 0.95 for F tests taking into consideration a large effect size (f = 0.4). Power calculations were carried out with Gpower 3.1.9.7 (Faul *et al*, 2009). Serum samples from 28 LUAD patients not eligible for immunotherapy treatment were obtained from the Hospital Fundación Jimenez Díaz (Madrid, Spain). All patients provided written informed consent for participation in the study, approved by the Fundación Jiménez Díaz (CEImJGD, ref. ER_EO180‐19_FJD‐HGV). As a validation cohort, 18 patients diagnosed with locally advanced or metastatic NSCLC treated with the combination of chemo‐immunotherapy (pembrolizumab + platinum‐based chemotherapy) as first‐line treatment were recruited between October 2020 and October 2022 at the Oncology Department of the University Hospital of Navarra (Pamplona, Spain), from whom written informed consents were obtained. Samples were processed by M.G., and plasma FKN concentrations were determined by Luminex assays by B.T.

Eight milliliters of peripheral blood samples were obtained prior to and during immunotherapy before administration of each cycle. PBMCs were isolated as described (Escors *et al*, 2008; Bocanegra *et al*, 2019; Zuazo *et al*, 2019), and myeloid cells were analyzed by flow cytometry. The participation of each patient concluded when a radiological test confirmed response or progression, with the withdrawal of consent or after death of the patient. Tumor responses were evaluated according to RECIST 1.1 (Eisenhauer *et al*, 2009) and Immune‐Related Response Criteria (Wolchok *et al*, 2009). Objective responses were confirmed by at least one sequential tumor assessment.

### Cell lines and *in vitro* cell‐based assays

Work with all cell lines was carried out under biosafety 2 conditions. 3LL mouse adenocarcinoma cells were obtained from the American Type Culture Collection (CRL‐1642™). Cells were grown in DMEM (Gibco) supplemented with 10% FBS and 1% penicillin/streptomycin following standard procedures. The murine lung adenocarcinoma cell line Lacun‐3 was transferred from Prof. Luis Montuenga's group. Five human lung adenocarcinoma cell lines (Calu6‐ HTB‐56, H23‐CRL‐5800, A549‐ CRM‐CCL‐185, HCC44‐CRL‐5896, H358‐ CRL‐5807) and 5 human lung squamous carcinoma cell lines (H520‐ HTB‐182, H1703‐ CRL‐5889, H226‐CRL‐5826, H2170‐ CRL‐5928, SW900‐ HTB‐59) were obtained from the American Type Culture Collection and grown following standard procedures.

When indicated, cell lines were engineered to constitutively overexpress soluble forms of GM‐CSF, G‐CSF, M‐CSF, and FKN. Their coding sequences were synthesized (GeneArt Thermo Fisher) and cloned into pDUAL‐Puromycin lentivectors (Gato‐Canas *et al*, 2017) under the transcriptional control of the SSFV promoter. Lentivector production, titration, cell transduction, and selection with puromycin were carried out as described elsewhere (Gato‐Canas *et al*, 2017). Cytokine production was confirmed by ELISA. Cell growth and survival were monitored in real time using xCELLigence real‐time cell analysis (RTCA ACEA Biosciences) as described before (Gato‐Canas *et al*, 2017).

### Flow cytometry

PBMC isolation, staining, and flow cytometry were performed as described (Gato‐Canas *et al*, 2017; Zuazo *et al*, 2019). The following fluorochrome‐conjugated anti‐human antibodies were used at 1:50 dilutions unless otherwise stated: CD206 (15‐2), CD124 (G077F6), LAG3 (11C3C65), CD38 (HB‐7), CD69 (FN50), CD115 (9‐4D2‐1E4), PD‐L1 (29E.2A3), C3AR (hC3Ar28, 1:200 diluted), CD64 (10.1, 1:100 diluted), CD32 (FUN‐2, 1:500 diluted), CCR7 (G043H7), CD36 (5‐271, 1:500 diluted), CD27 (M‐T271), CD28 (CD28.2), CD8 (SK1) (Biolegend), CD163 (GHI/61.1), CD39 (REA739), TIM3 (F38‐2E2), CD33 (AC104.3E3), VEGFR1 (REA569), CD16 (REA423), PD‐1 (PD‐1.3.1.3), CD62L (145/15), CD10 (REA877), C5AR (S5/1), CD66B (REA306), CCR2 (REA624), CD56 (AF12‐7H3), CD116 (REA211), CD4 (REA623), CD3 (REA613), CXCR1 (REA958), CXCR2 (REA2D8), CXCR4 (12G5), CX3CR1 (REA385) (Miltenyi), CD11b (M1/70, 1:300 diluted), CD14 (61D3, 1:20 diluted), CD86 (IT2.2), HLA‐DR (L243, 1:25 diluted), CD54 (15.2), CD19 (SJ25C1), CD11c (3.9) (Tonbo), VISTA (B7H5DS8) (Invitrogen).

For immunophenotyping of circulating cell populations and tumor infiltrates in murine models, blood was retrieved from mice (50 μL) in EDTA‐coated microtubes. Erythrocytes were lysed with BD Pharm Lyse solution for 1 min and the resulting cell suspensions were stained and analyzed by flow cytometry. Tumors and spleens were harvested and mechanically disaggregated. Erythrocytes were lysed and the single‐cell suspensions were stained and analyzed by flow cytometry. The following fluorochrome‐conjugated anti‐mouse antibodies were used: Ly6C (REA796), F4/80 (REA126), TIGIT (REA536) (Miltenyi), Ly6G (1A8), CD115 (AFS98), MHC‐II (M5/114.15.2), CX3CR1 (SA011F11), CCR2 (SA203G11), NK1.1 (PK136), CD4 (GK1.5), CD8 (53‐6.7), KLRG1 (2F1/KLRG1), TIM3 (RMT3‐23), PD‐1 (29F.1A12) (Biolegend), CD45 (30‐F11, 1:250 diluted), CD11c (HL3) (BD Pharmigen), CD11b (M1/70), CD3 (145‐2C11) (Tonbo), CD45 (104, 1:250 diluted), CD19 (eBio1D3) (eBioscience), CD25 (7D4) (Southern Biotech).

All samples were acquired in a FACS Canto II flow cytometer (Becton Dickinson). Flow cytometry data were exported as FCS3.0 files and analyzed using FlowJo or SPADE software.

The clustering algorithm SPADE (spanning tree progression analysis of density‐normalized events, Stanford University) was used to integrate and analyze multiple flow cytometry panels using internal FSC‐SSC patterns and common CD11b and CD14 as overlapping markers to reconstruct SPADE trees (Qiu *et al*, 2011). Diversity indexes were defined as the total number of terminal branches within each hierarchical cluster tree.

### Cytokine quantification

Human plasma samples were obtained from 8 ml‐EDTA blood tubes from each patient. Murine blood samples were collected by facial vein puncture in EDTA‐coated microtubes and plasma obtained by standard procedures. Quantification of plasma soluble cytokines, chemokines, and soluble immune checkpoint concentrations in plasma samples was carried out by Luminex xMAP technology following the manufacturer's instructions. A Human Cytokine/Chemokine magnetic bead panel (HCYTOMAG‐60K, Millipore) was used to measure the concentrations of CD40L, CCL11, IFNα, IFNγ, IL2, IL4, IL6, IL8, IL12, IL17, TNFα, VEGFA, FGF2, FKN, G‐CSF, GM‐CSF, IL1β, IL1Ra, IL3, IL5, IL7, IL9, IL10, IL15, CXCL10, MCP1, CCL7, CCL3, and CCL4. A human Immuno‐oncology checkpoint protein magnetic bead panel (HCKPMAG‐11K, Millipore) was used to quantify concentrations of soluble BTLA, CD27, CD28, TIM3, HVEM, CD40, GITR, LAG3, TLR2, GITRL, PD‐1, CTLA4, CD80, CD86, PD‐L1, and ICOS. A Human Immuno‐oncology checkpoint protein magnetic bead panel 2 (HCKP2‐11K, Millipore) was used to quantify concentrations of soluble arginase‐1, ICOSL, CD276, CD73, VTCN1, APRIL, VISTA, B7‐H6, granzyme B, E‐cadherin, galectin‐1, galectin‐3, granulysin, IDO1, MIC‐A, MIC‐B, BAFF, OX40, CD155, and perforin. Final detection and data analyses were performed on a MAGPIX (EMD Millipore) with xPONENT software.

Quantification of plasma soluble FKN in murine plasma samples was carried out by ELISA following the manufacturer's instructions (R&D DuoSet DY472 ELISA kit). Quantification of secreted M‐CSF, G‐CSF, FKN, and GM‐CSF from cell cultures was carried out by ELISA (R&D DuoSet ELISA kits; DY415‐05 for GM‐CSF; DY414‐05 for G‐CSF; DY416‐05 for M‐CSF; MCX310 for FKN). Secreted FKN concentration was quantified in supernatants of human cancer cell lines by ELISA (DY365 R&D DuoSet ELISA kit).

### Mouse lung cancer models and therapies

Approval for animal studies was obtained from the Animal Ethics Committee of the University of Navarra (Pamplona, Navarra, Spain. Reference 077‐19 and 064‐22) and from the Government of Navarra. All animals were housed at CIMA's animal house facilities (conventional biosafety 2 housing conditions with environmental enrichment, ES31 2010000132, University of Navarre). When males were used for experiments, they were housed in individual cages if dominant behaviors and barbering were observed. Randomization was used to allocate mice into cages. ARRIVE guidelines were followed for animal experimentation. The indicated 3LL cell lines (1.5 × 10^6^ cells/mouse; *n* = 6 mice/group) were subcutaneously injected in the flanks of 10‐week‐old C57BL/6 female and male mice (Envigo). 3LL engraftments were allowed to grow for 7 days, then mice were intraperitoneally treated with 100 μg of anti‐PD‐1 (RPMI‐14, BioXCell) or saline buffer as control vehicle at days 7, 11, and 15 after tumor inoculation. Tumor size was measured three times per week with a digital caliper until humane endpoint was reached (tumor large diameter superior to 14 mm). Tumor volumes (V) were calculated as V (mm^3^) = [(short diameter)^2^ × (long diameter)]/2. Analyses of circulating immune populations were carried out on day 15 after tumor engraftment. For immunophenotyping of tumor immune infiltrates and spleens, tumors and spleens were harvested and mechanically disaggregated 14 days after engraftment. To assess the abscopal effects of systemic FKN, 3LL or 3LL‐FKN cells were subcutaneously injected (1.5 × 10^6^ cells/mouse; *n* = 6 mice/group) on the left flank of mice. Seven days after tumor engraftment, 3LL cells were injected on the right flank. 3LL engraftments were allowed to grow for 7 days, then mice were intraperitoneally treated with 100 μg of anti‐PD‐1 (RPMI‐14, BioXCell) or saline buffer as control vehicle at days 7, 11, and 15 after tumor inoculation. *In vivo* NK, CD4, and CD8 T‐cell depletions were carried out by intraperitoneal administration of 100 μg of anti‐mouse CD8a (clone 2.43; BioXCell), CD4 (clone GK1.5; BioXCell) or NK1.1 (clone PK136; BioXCell) antibodies, respectively, at days 6, 10, 14 and 18 after 3LL tumor inoculation.

In some experiments, intraperitoneal administration of 100 μg/kg of recombinant FKN was performed (Biotechne) 6 days after tumor inoculation (1.5 × 10^6^ cells/mouse; *n* = 6 mice/group) in 10‐week‐old C57BL/6 male mice (Envigo). When indicated, intraperitoneal injection of the CX3CR1 chemical inhibitor AZD8797 (MedChemExpress) was carried out with 0.8 mg/kg at days 6, 8, 11, 13, and 15 after tumor engraftment. Anti‐trinitrophenol IgG2a isotype control (clone 2A3, BioXCell) was administered as a nonrelevant protein control.

### Immunostaining analysis

Formalin‐fixed paraffin‐embedded tissues from 10 LUAD patients were obtained from the Hospital San Pedro (Logroño, Spain). All patients provided written informed consent for participation in the study, approved by the Ethics Committee of Clinical Research of La Rioja (CEICLAR, ref. PI‐205). Paraffin‐embedded lung tissues from LUAD patients were cut into 3 μm sections. Immunostaining analysis to evaluate PanCK and FKN positive areas (%) was performed using Pan‐Cytokeratin (Clone AE1/AE3 1:330, Santa Cruz Biotech. Inc., Dallas, TX) and fractalkine (1:230, Abcam, Cambridge, UK) primary antibodies and the Novolik Polymer Detection System (Leica Biosystems, Nussloch, Germany). Fiji open‐source image processing software package v1.48r (http://fiji.sc) was used to quantify PanCK and FKN positive areas using the color deconvolution tool. Statistical analyses were performed using SPSS Statistics Software v21 for Windows (IBM, Armonk, NY, USA). According to the sample distribution (Shapiro–Wilk normality test), either a Mann–Whitney or unpaired *t*‐tests were used to compare differences between 2 independent groups. Pearson's correlation coefficients between PanCK and FKN positive areas were calculated using GraphPad Prism v8.3 (GraphPad Software Inc., San Diego, CA, USA). For all analyses, a *P*‐value < 0.05 was considered statistically significant.

### Transcriptomic data analyses

To assess the association of FKN mRNA expression with infiltration of different immune populations in human lung adenocarcinomas, we used Tumor Immune Estimation Resource or TIMER2.0 (http://timer.cistrome.org/; Li *et al*, 2020). This resource contains transcriptional data from samples included in the TCGA database, including the abundance of immune cells across multiple tumor types. It generates a graphical representation of user‐selected gene expression and Spearman correlations with immune populations of interest as identified by CIBERSORT, quanTIseq, xCell, and TIDE algorithms. We restricted our analysis to lung adenocarcinomas and performed systematic correlation studies with CD8 lymphocytes, CD4 lymphocytes, regulatory T lymphocytes, B cells, NK cells, neutrophils, monocytes, macrophages, dendritic cells, and MDSCs. A partial purity‐adjusted Spearman's correlation was used. Correlation between FKN, PD‐1, and PD‐L1 expression was performed by Spearman's correlation tests within GEPIA2 (Tang *et al*, 2019).

### Statistical analysis and study design

No data were considered an outlayer, or removed from the analyses. For mouse experiments, sample sizes were calculated to achieve a minimum power of 0.8 for F‐based tests taking into consideration a large effect size (f = 04). Power calculations were carried out with Gpower 3.1.9.7. Blinding was used for data analysis, and double blinding for the validation cohort in FKN plasma quantification and correlation with survival. All variables were tested for normality using the Kolgomorov–Smirnov test. Homogeneity was assessed with Spearman's coefficient of variations. Samples were considered homogeneous with CV < 25%. Homogeneity of variances was tested by the F‐Fisher test. Homogeneous groups with comparable variances and fulfilling normality were compared using parametric tests. For multicomparisons, one‐way ANOVAs were used followed by pairwise comparisons with *a posteriori* Tukey's tests. Variables fulfilling these criteria were (1) diversity indexes as calculated below; (2) cell growth rates and cell index data from ACEA Real‐Time Cell monitoring (RTCA) data; (3) Tumor volumes as measured at the indicated time points; (4) FKN plasma concentrations in tumor‐bearing mice, and (5) secreted FKN concentrations from cultures of cancer cell lines.

The rest of the variables were tested with nonparametric tests. For multicomparisons, one‐way Kruskal–Wallis tests were used followed by *a posteriori* pairwise comparisons with the Dunn's test. Variables that were normally distributed but did not fulfill homogeneity of variances were also tested with nonparametric tests. These included (1) percentages of monocytes and neutrophils quantified by high‐dimensional flow cytometry; (2) human serum cytokine concentrations, and (3) percentages of circulating and tumor‐infiltrating immune cell types quantified by flow cytometry, in mouse tumor models.

DI in circulating CD11b^+^ cells was calculated as the number of clusters with terminal phenotypes from SPADE 3 hierarchical cluster analyses using high‐dimensional flow cytometry data. Correlations between DI and other variables (which included percentages of monocytes, neutrophils, and serum concentrations of relevant factors) were evaluated by Spearman's tests. The predictive capacities of the selected indicated variables were assessed by ROC analyses as described before (Zuazo *et al*, 2019). PFS and OS in human patients and mouse tumor models were compared with log‐rank tests. Tumor experiments were independently replicated between 2 and 7 times (sensitization of PD‐1 refractory tumors by FKN). *In vitro* experiments were replicated at three independent times at least.

## Author contributions

**Ana Bocanegra:** Resources; data curation; software; formal analysis; validation; investigation; visualization; methodology; writing – original draft; writing – review and editing. **Gonzalo Fernández‐Hinojal:** Resources; data curation; software; formal analysis; investigation; methodology. **Daniel Ajona:** Resources; investigation; methodology. **Ester Blanco:** Resources; investigation; methodology. **Miren Zuazo:** Resources; investigation; methodology. **Maider Garnica:** Resources; investigation; methodology. **Luisa Chocarro:** Resources; validation; investigation; methodology. **Elvira Alfaro‐Arnedo:** Resources; software; validation; investigation; visualization; methodology. **Sergio Piñeiro‐Hermida:** Resources; data curation; software; validation; investigation; visualization; methodology. **Pilar Morente:** Investigation; methodology. **Leticia Fernández:** Resources; investigation; methodology. **Ana Remirez:** Resources; software; validation; investigation; methodology. **Miriam Echaide:** Resources; investigation; methodology. **Maite Martinez‐Aguillo:** Resources; investigation; methodology. **Idoia Morilla:** Resources; investigation; methodology. **Beatriz Tavira:** Resources; software; validation; investigation; visualization; methodology. **Alejandra Roncero:** Resources; software; validation; investigation; methodology. **Carolina Gotera:** Resources; validation; investigation; methodology. **Alfonso Ventura:** Resources; validation; investigation. **Nerea Recalde:** Resources; validation; investigation. **José G Pichel:** Resources; validation; investigation; methodology. **Juan José Lasarte:** Resources; investigation. **Luis Montuenga:** Resources; investigation; methodology. **Ruth Vera:** Resources; investigation; methodology. **Ruben Pio:** Resources; validation; investigation; methodology. **David Escors:** Conceptualization; resources; data curation; software; formal analysis; supervision; funding acquisition; validation; investigation; visualization; methodology; writing – original draft; project administration; writing – review and editing. **Grazyna Kochan:** Conceptualization; resources; data curation; software; formal analysis; supervision; funding acquisition; validation; investigation; visualization; methodology; writing – original draft; project administration; writing – review and editing.

## Disclosure and competing interests statement

The authors declare that they have no conflict of interest.

## Supporting information



Expanded View Figures PDF

Table EV1

Table EV2

PDF+

## Data Availability

This study includes no data deposited in external repositories.
