# Supplementary material for: Plasma fractalkine contributes to systemic myeloid diversity and PD‐L1/PD‐1 blockade in lung cancer
Source: EMBO Rep. 2023 Jun 27;24(8):e55884. doi: 10.15252/embr.202255884 (PMC10398648; doi:10.15252/embr.202255884)
Supplement: Supplementary file 1 — Expanded View Figures PDF [file EMBR-24-e55884-s001.pdf]

Expanded View Figures

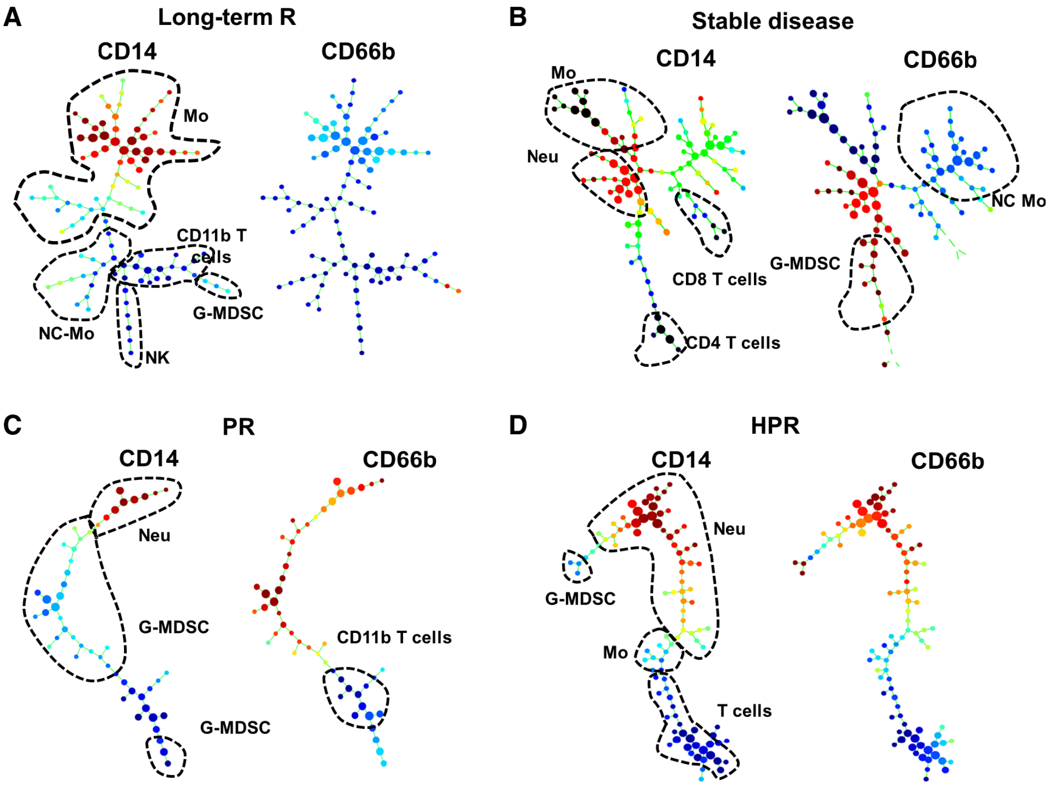

Figure EV1. Hierarchical phenotype clustering from high-dimensional flow cytometry data of baseline multiple cell lineage and activation markers within immune cell subsets in PBMCs from NSCLC patients undergoing anti-PD-1/PD-L1 immunotherapy.

A–D Representative SPADE3 cluster profiles of myeloid cells integrating 43 markers are shown for (A) a long-term responder, (B) a short-term responder (stable disease), (C) a progressor (PR), and (D) a hyperprogressor (HPR). Distributions of CD14 (left dendrogram) and CD66b expression (right dendrogram) are shown. Main cell subsets are encircled and identified as Mo—monocytes; Neu—neutrophils; G-MDSC—granulocytic myeloid-derived suppressor cells; NC-Mo—nonclassical monocytes; NK—natural killer cells. The relative expression of the selected marker as indicated above the graphs color-coded, from dark red (maximum expression) to dark blue (minimum expression).

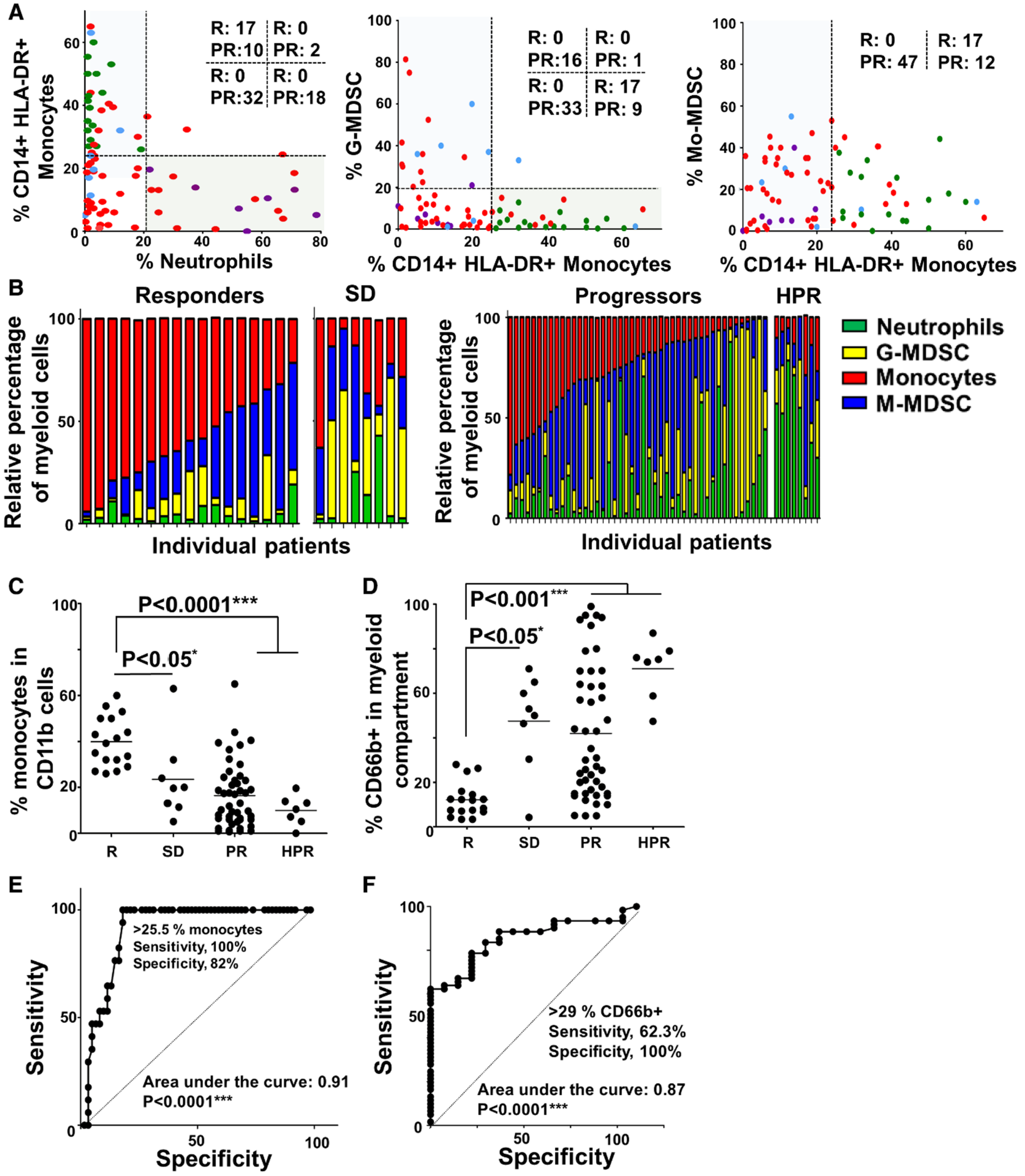

Figure EV2.

 © 2023 The Authors

Figure EV2. Baseline monocyte/neutrophil profiles in peripheral blood from NSCLC patients undergoing PD-L1/PD-1 blockade.

A Left graph, baseline frequency of monocytes ($CD14^+$ $HLA\text{-}DR^+$) vs neutrophils ($CD14^+$ $CD66b^+$) within $CD11b^+$ cells in patients classified as responders (R, green), progressors (PR, red), stable disease (blue) and hyperprogressors (purple). The number of responders and progressors is indicated in each quadrant. Center graph, as in left but plotting the percentage of G-MDSCs ($CD14^-$ $CD66b^+$) vs monocytes. Right graph, as in left but plotting the percentage of Mo-MDSC ($CD14^+$ $HLA\text{-}DR^-$) vs. monocytes.

B Left graph, relative percentages of the main myeloid populations restricted to this compartment are plotted for each patient under study as indicated as color codes, classified according to objective responders and stable disease (SD); Right graph, as in left but in progressors and hyperprogressors (HPR).

C Percentage of circulating monocytes ($CD11b^+$ $CD14^+$ $HLA\text{-}DR^+$) within each response group as indicated.

D Percentage of granulocytic cells ($CD11b^+$ $CD66b^+$) within each response group as indicated.

E ROC analysis of the percentage of monocytes as a predictor of objective responses.

F ROC analysis of the percentage of granulocytic myeloid cells as a predictor of no objective response.

Data information: R—objective responders (n = 16); SD—stable disease (n = 9); PR—progressors (n = 47); HPR—hyperprogressors (n = 7); ns—nonstatistical differences. Relevant statistical comparisons are shown in the graphs. Multicomparisons in dot plots were carried out by the Wilcoxon test. Pairwise comparisons were performed by the Mann–Whitney *U* test. *, ***, ****, indicate significant ($P < 0.05$), highly significant ($P < 0.001$), and very highly significant ($P < 0.0001$) differences.

© 2023 The Authors

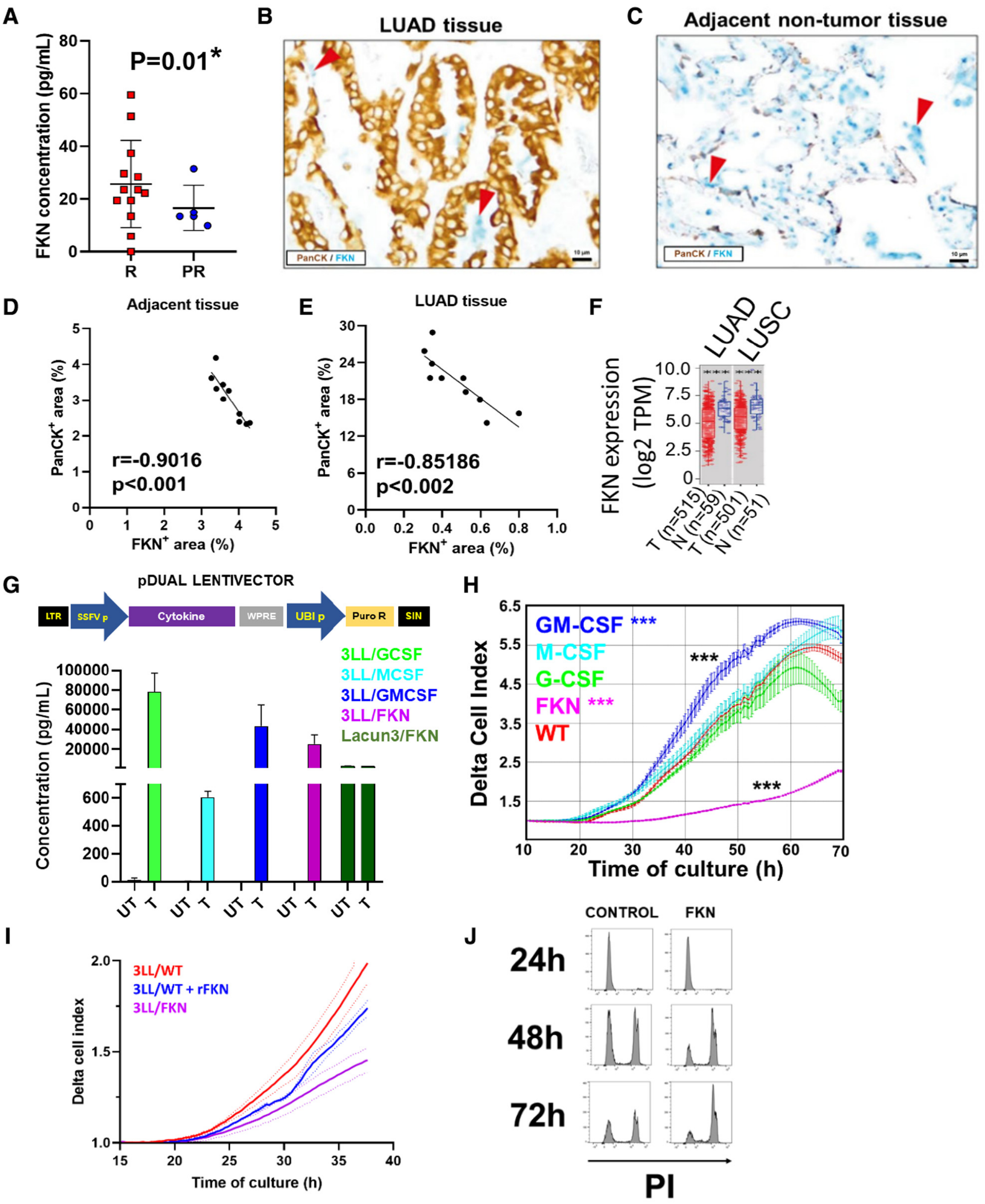

Figure EV3.

 © 2023 The Authors

Figure EV3. FKN expression in lung cancer patients and engineering of mouse lung cancer cell lines overexpressing myeloid-regulating cytokines.

A Baseline plasma FKN concentrations in responders (R, $n = 13$) and progressors (PR, $n = 5$) in a validation cohort of NSCLC patients treated with anti-PD-1/PD-L1 immunotherapy as a first-line treatment. Error bars are shown (standard deviations, SD). Statistical significance was tested by the chi-square test.

B, C (B) Representative double lung immunostainings for Pan-Cytokeratin (PanCK) (brown) and FKN (blue; red arrows indicate FKN positive cells) in lung tissue sections from LUAD patients and (C) adjacent healthy tissue.

D, E (D) Pearson's correlation of PanCK with FKN positive areas (%) in histologies of adjacent nontumor and (E) LUAD tissues. Pearson's correlation coefficients are shown in the graphs. $n = 10$.

F FKN mRNA expression level in tumor (T) and normal tissue (N) of clinical samples from lung adenocarcinoma (LUAD) and squamous lung carcinoma (LUSC) patients registered in the TCGA database. Distributions of gene expression levels are displayed using box plots. Box and whisker plots indicate median (central line), 25th to 75th percentiles (box), and minimum to maximum values (whiskers).

G Top, lentivectors for the expression of cytokines of interest. SIN, self-inactivating deleted LTR; LTR, long-terminal repeat; SFFVp, spleen focus-forming virus promoter; UBIp, human ubiquitin promoter; Puro R, puromycin resistance gene. Down, ELISA quantification of cytokine secretion by the indicated engineered lung cancer cell lines expressing the indicated cytokines (T). Endogenous secretion of each cytokine was also quantified in supernatants from cultures of parental unmodified controls (UT). Data are presented as mean ± SD ($n = 3$ independent biological replicates).

H Real-time cell growth (RTCA) of 3LL cell lines engineered to secrete the indicated cytokines. Relevant statistical comparisons of delta-cell indexes after 50 h of culture were carried out by ANOVA. Data are presented as mean ± SD ($n = 3$ independent biological replicates).

I Real-time cell growth (RTCA) of unmodified and FKN-producing 3LL cell lines cultured with 1 μg/ml of recombinant FKN (rFKN).

J Flow cytometric assessment of propidium iodide (PI) uptake by apoptotic unmodified 3LL cells cultured in 3LL-WT (control) or 3LL-FKN (FKN) conditioned medium for 24, 48, or 72 h.

Data information: The statistical significance computed by the Wilcoxon test is annotated by the number of stars. *, **, *** indicate significant ($P < 0.05$), very significant ($P < 0.01$) and highly significant ($P < 0.001$) differences.

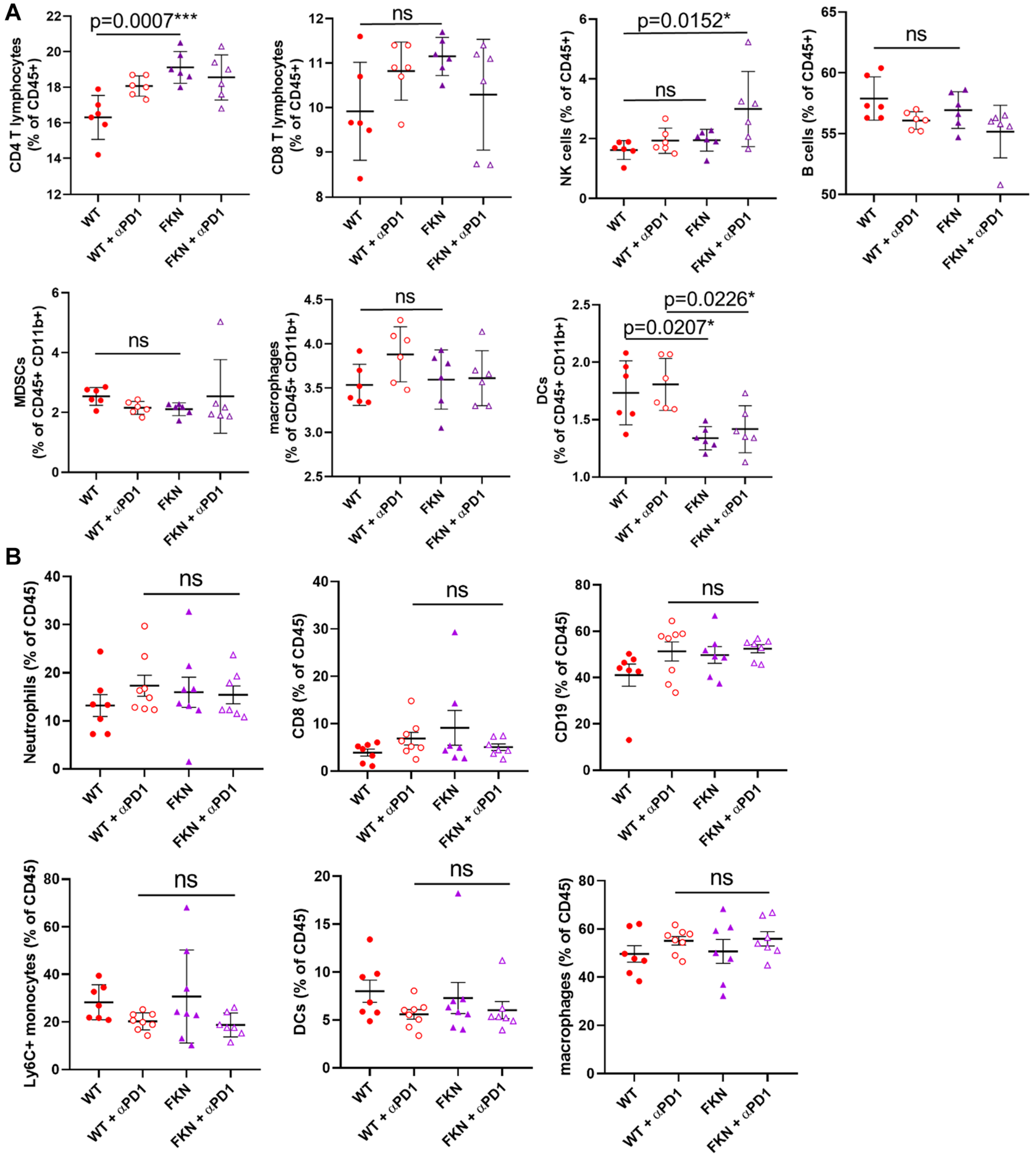

Figure EV4. Immune profiling in mice transplanted with 3LL cells expressing FKN and its combination with PD-1 blockade.

A The graphs represent percentages of the indicated infiltrating immune cell types as quantified by flow cytometry, in spleens obtained from mice inoculated with the indicated cell lines (parental cell line, WT; 3LL cells expressing FKN, FKN) with or without PD-1 blockade treatment. CD4 and CD8 T lymphocytes, NK cells (NK1.1), B cells (CD19), MDSCs (Ly6G^{+} CD115^{+}), macrophages (F4/80), and DCs (CD11c) were quantified at day 14 after tumor inoculation. Data are shown as the mean of the percentage within total leukocytes (CD45^{+}) ± SD (n = 6 mice). Relevant statistical comparisons are shown in the graphs, evaluated by ANOVA and Tukey's pairwise comparisons. *, ***, indicate significant ($P < 0.05$) and highly significant ($P < 0.001$) differences. ns, nonsignificant differences.

B Graphs represent percentages of the indicated infiltrating immune cell types as quantified by flow cytometry, in tumors excised from mice inoculated with the indicated cell lines (parental cell line, WT; 3LL cells expressing FKN, FKN) with or without PD-1 blockade. Neutrophils (Ly6G), CD8, B cells (CD19), Ly6C^{+} monocytes, DCs (CD11c), and macrophages (F4/80) were quantified at day 14 after tumor inoculation. Data are shown as the mean of the percentage within total leukocytes (CD45^{+}) ± SD (n = 8 mice). Relevant statistical comparisons are shown in the graphs, evaluated by ANOVA and Tukey's pairwise comparisons. ns, nonsignificant differences.

 © 2023 The Authors

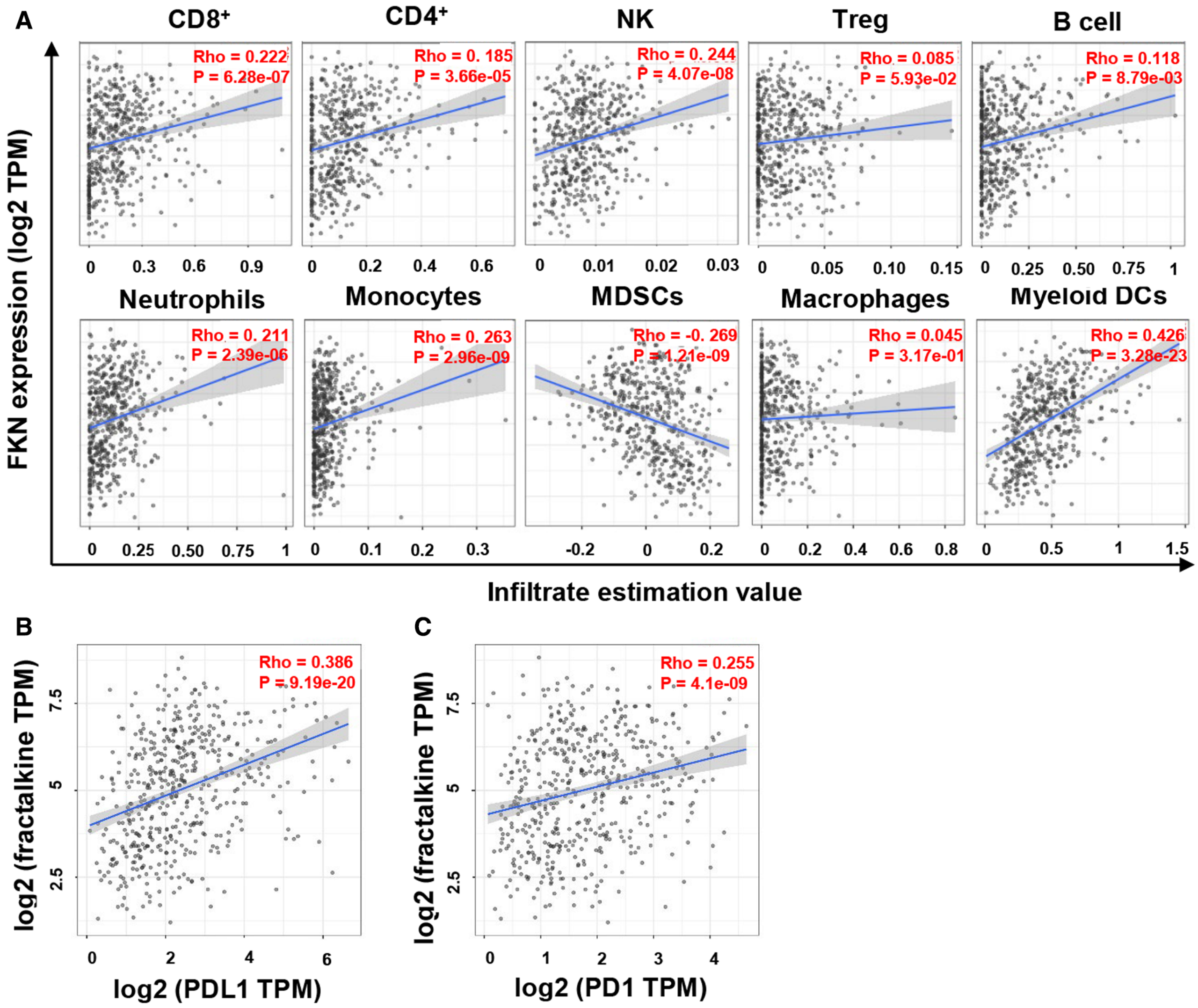

Figure EV5. FKN transcriptomic expression in human lung adenocarcinoma samples and correlation with tumor immune infiltration, survival, and PD-L1 tumor expression.

A Evaluation of tumor infiltration with the indicated selected immune cell populations, and correlation analyses of FKN transcriptional expression and immune infiltrates from the TCGA database. Analyses were restricted to lung adenocarcinoma samples (n = 515). Spearman correlation with different immune populations was identified by several algorithms (CIBERSORT, quanTIseq, xCell, TIDE) and an adjustment based on tumor purity was employed to minimize the potential interaction of low tumor cell quantities. Each dot represents a single tumor sample. Spearman's rho value and P-values are provided within the graphs.

B Correlation between tumor FKN and PD-L1 transcriptional expression by the Spearman's test. Relevant statistical results are presented within the graphs.

C As in (B) but plotting PD-1 transcriptional expression levels.

© 2023 The Authors
