# Supplementary material for: Plasma fractalkine contributes to systemic myeloid diversity and PD‐L1/PD‐1 blockade in lung cancer
Source: EMBO Rep. 2023 Jun 27;24(8):e55884. doi: 10.15252/embr.202255884 (PMC10398648; doi:10.15252/embr.202255884)
Supplement: Supplementary file 2 — Table EV1 [file EMBR-24-e55884-s002.docx]

***Table EV1. Patient cohort characteristics.***

| **Variable** | **All patients (n=112)** |
| --- | --- |
| **Sex** |  |
| Female | 28 (25.0 %) |
| Male | 84 (75.0 %) |
| **Age** |  |
| <60 | 31 (27.7 %) |
| ≥60 | 81 (72.3 %) |
| **Histology** |  |
| Squamous | 28 (25.0 %) |
| Non- Squamous | 84 (75.0 %) |
| **Immunotherapy treatment** |  |
| Pembrolizumab (αPD-1) | 32 (28.6 %) |
| Nivolumab (αPD-1) | 34 (30.3 %) |
| Atezolizumab (αPD-L1) | 33 (29.5 %) |
| Pembrolizumab + platinum-based chemotherapy | 13 (11.6 %) |
| **Tumor PD-L1 status** |  |
| 0% | 38 (34.0 %) |
| 1-4% | 12 (10.7 %) |
| 5-49% | 20 (17.8 %) |
| ≥ 50% | 33 (29.5 %) |
| Undetermined | 9 (8.0 %) |
| **Mutation status** |  |
| Not evaluated | 108 (96.4 %) |
| EGFR | 1 (0.9 %) |
| ROS1 | 1 (0.9 %) |
| KRAS | 1 (0.9 %) |
| METamp | 1 (0.9 %) |
| **Smoking status** |  |
| Smoker | 106 (94.6 %) |
| Non-smoker | 6 (5.4 %) |
| **Treatment line** |  |
| 1st | 38 (33.9 %) |
| 2nd | 61 (54.5 %) |
| 3th | 10 (8.9 %) |
| 4th or higher | 3 (2.7 %) |
| **Previous systemic therapies (previous 3 months)** |  |
| Platinum-based therapy | 28 (25.1 %) |
| Non-platinum based therapy | 36 (32.1 %) |
| No | 48 (42.8 %) |
| **Liver metastases** |  |
| No | 83 (74.1 %) |
| Yes | 29 (25.9 %) |
| **Number of metastatic sites** |  |
| ≤2 | 33 (29.5 %) |
| ≥3 | 79 (70.5 %) |
| **Responses** |  |
| Partial response | 29 (25.9 %) |
| Progression disease | 65 (58.0 %) |
| Hyperprogression | 5 (4.5 %) |
| Stable disease | 13 (11.6 %) |
