# Supplementary material for: Plasma fractalkine contributes to systemic myeloid diversity and PD‐L1/PD‐1 blockade in lung cancer
Source: EMBO Rep. 2023 Jun 27;24(8):e55884. doi: 10.15252/embr.202255884 (PMC10398648; doi:10.15252/embr.202255884)
Supplement: Supplementary file 3 — Table EV2 [file EMBR-24-e55884-s003.docx]

***Table EV2. Concentration of plasma soluble-proteins quantified by luminex prior to immunotherapy (Baseline) and after the first cycle of treatment (1C).*** *Data are expressed as mean (pg/mL) ± SD. R: responders; PR: non-responders; SD: stable disease; HPR: hyperprogressors; H: age-matched healthy donors.*

|  | **Baseline** | | | | |
| --- | --- | --- | --- | --- | --- |
| **Analyte** | **R** | **PR** | **SD** | **HPR** | **H** |
| **FGF-2** | 49.3 ± 32.8 | 44.1 ± 35.5 | 65.4 ± 41.6 | 29.9 ± 19.4 | 20.4 ± 14.2 |
| **Eotaxin** | 104.4 ± 64.9 | 97.6 ± 55.7 | 87.8 ± 43.5 | 76.8 ± 38.7 | 58.9 ± 21.9 |
| **G-CSF** | 68.8 ± 130.2 | 33.7 ± 28.3 | 39.3 ± 55.2 | 13.7 ± 5.8 | 30.3 ± 18.7 |
| **GM-CSF** | 11.2 ± 12.2 | 8.1 ± 9.3 | 8.9 ± 11.1 | 7.8 ± 4.6 | 0.3 ± 0.5 |
| **FKN** | **62.3 ± 35.0** | **45.7 ± 33.6** | **97.3 ± 123.3** | **36.6 ± 7.1** | **31.1 ± 27.6** |
| **IFNα** | 27.6 ± 28.7 | 23.2 ± 24.2 | 63.4 ± 65.1 | 16.5 ± 14.4 | 16.6 ± 11.2 |
| **IFNγ** | 7.1 ± 7.0 | 5.4 ± 4.5 | 21.5 ± 47.7 | 2.6 ± 2.2 | 3.5 ± 2.5 |
| **IL-10** | 12.4 ± 23.4 | 16.7 ± 38.2 | 10.4 ± 15.4 | 3.6 ± 5.8 | 0.0 ± 0.0 |
| **IL12p70** | 7.2 ± 17.2 | 4.7 ± 14.6 | 2.5 ± 1.6 | 1.2 ± 1.1 | 0.9 ± 1.1 |
| **IL-15** | 0.5 ± 1.3 | 1.0 ± 1.5 | 1.4 ± 2.1 | 0.8 ± 1.4 | 0.0 ± 0.0 |
| **sCD40L** | 85.7 ± 89.6 | 291.7 ± 982.5 | 88.2 ± 124.8 | 48.2 ± 29.6 | 69.4 ± 24.8 |
| **IL-17** | 2.6 ± 2.8 | 2.5 ± 3.0 | 5.8 ± 8.3 | 0.6 ± 0.6 | 4.3 ± 0.5 |
| **IL1RA** | 17.9 ± 11.2 | 29.3 ± 33.4 | 30.8 ± 43.0 | 22.0 ± 3.1 | 30.0 ± 3.5 |
| **IL-1β** | 0.6 ± 0.8 | 0.9 ± 1.8 | 6.3 ± 13.0 | 0.4 ± 0.3 | 0.9 ± 1.3 |
| **IL-2** | 1.2 ± 1.3 | 0.9 ± 0.7 | 0.8 ± 0.6 | 0.4 ± 0.4 | 1.3 ± 0.5 |
| **IL-3** | 0.6 ± 0.7 | 0.9 ± 0.7 | 0.9 ± 0.7 | 0.5 ± 0.5 | 1.6 ± 0.1 |
| **IL-5** | 0.8 ± 0.6 | 1.6 ± 4.6 | 0.5 ± 0.7 | 0.5 ± 0.1 | 0.1 ± 0.1 |
| **IL-6** | 4.3 ± 7.6 | 6.7 ± 17.3 | 0.0 ± 0.0 | 0.1 ± 0.3 | 0.0 ± 0.0 |
| **IL-7** | 2.3 ± 2.8 | 3.1 ± 6.8 | 1.3 ± 1.6 | 1.3 ± 1.3 | 0.0 ± 0.0 |
| **IL-8** | 14.4 ± 17.4 | 24.6 ± 57.2 | 6.7 ± 3.3 | 3.8 ± 2.5 | 2.5 ± 2.2 |
| **IP-10** | 601.8 ± 361.2 | 685.5 ± 652.8 | 1019.6 ± 1331.8 | 543.9 ± 83.7 | 316.6 ± 124.7 |
| **MCP-1** | 261.2 ± 132.1 | 404.9 ± 369.5 | 226.4 ± 150.3 | 427.3 ± 175.7 | 491.4 ± 164.9 |
| **MIP-1a** | 2.4 ± 2.6 | 2.0 ± 2.4 | 2.4 ± 3.1 | 2.6 ± 1.3 | 0.0 ± 0.0 |
| **MIP-1b** | 23.9 ± 17.8 | 24.7 ± 12.5 | 19.1 ± 9.3 | 26.2 ± 11.4 | 20.8 ± 2.7 |
| **TNFα** | 10.9 ± 4.8 | 13.1 ± 6.3 | 12.4 ± 11.4 | 16.0 ± 4.8 | 12.2 ± 1.6 |
| **VEGF** | 36.1 ± 29.0 | 54.1 ± 76.8 | 48.3 ± 100.2 | 27.9 ± 10.8 | 16.6 ± 19.3 |
| **BTLA** | 1515.0 ± 1066.9 | 1772.1 ± 1416.2 | 1095.6 ± 685.2 | 1984.6 ± 1466.9 | 548.1 ± 270.4 |
| **CD27** | 1685.3 ± 1462.8 | 1784.8 ± 1155.3 | 1283.8 ± 983.3 | 2387.5 ± 976.6 | 1314.5 ± 552.6 |
| **CD28** | 3855.6 ± 2898.9 | 5238.6 ± 4815.2 | 9069.7 ± 20678.0 | 5640.1 ± 3279.7 | 2614.9 ± 1477.9 |
| **TIM3** | 2177.8 ± 1092.2 | 3076.4 ± 1368.1 | 1908.8 ± 751.1 | 3247.4 ± 787.6 | 1793.5 ± 252.7 |
| **HVEM** | 1165.8 ± 454.2 | 1650.7 ± 897.7 | 1293.9 ± 1006.2 | 1485.0 ± 115.4 | 1069.0 ± 70.5 |
| **CD40** | 485.0 ± 143.9 | 688.3 ± 370.5 | 588.1 ± 447.5 | 488.8 ± 116.1 | 332.3 ± 16.4 |
| **GITR** | 281.5 ± 310.8 | 412.7 ± 408.7 | 417.7 ± 509.5 | 346.1 ± 184.0 | 52.2 ± 60.6 |
| **LAG3** | 44634.8 ± 24196.0 | 51257.0 ± 24837.0 | 55804.7 ± 23278.2 | 31734.9 ± 6971.1 | 31978.0 ± 10606.9 |
| **TLR2** | 3186.3 ± 1282.4 | 3826.4 ± 2607.1 | 4951.4 ± 7797.8 | 3358.5 ± 1880.4 | 1661.1 ± 577.2 |
| **GITRL** | 1035.0 ± 511.2 | 1166.1 ± 696.5 | 895.9 ± 561.5 | 1166.5 ± 602.7 | 554.3 ± 155.5 |
| **PD-1** | 1598.2 ± 777.8 | 1821.1 ± 1229.9 | 2978.6 ± 5506.0 | 1864.1 ± 724.7 | 587.8 ± 208.3 |
| **CTLA-4** | 86.1 ± 90.2 | 103.9 ± 95.1 | 72.3 ± 65.4 | 137.6 ± 63.6 | 30.4 ± 7.0 |
| **CD80** | 293.7 ± 165.5 | 350.4 ± 209.7 | 254.4 ± 131.0 | 317.5 ± 183.5 | 224.9 ± 92.5 |
| **CD86** | 2003.3 ± 1220.9 | 2190.9 ± 1817.6 | 1591.0 ± 869.8 | 2219.3 ± 1300.0 | 662.8 ± 236.7 |
| **PD-L1** | 319.3 ± 236.8 | 357.2 ± 234.8 | 291.2 ± 162.0 | 336.5 ± 181.3 | 124.2 ± 45.9 |
| **ICOS** | 4177.8 ± 2269.8 | 4482.4 ± 2686.6 | 3694.9 ± 2178.2 | 3199.1 ± 1083.1 | 1539.0 ± 585.7 |
| **Arginase1** | 438.6 ± 755.0 | 443.9 ± 602.6 | 399.5 ± 637.2 | 529.1 ± 393.2 | 399.6 ± 178.4 |
| **ICOSL** | 3780.9 ± 3056.9 | 3792.2 ± 2377.7 | 3272.5 ± 1437.6 | 6388.8 ± 2967.1 | 5185.4 ± 1074.3 |
| **CD276** | 923.8 ± 531.0 | 1150.4 ± 564.5 | 834.5 ± 426.4 | 1144.3 ± 480.4 | 1420.0 ± 448.1 |
| **CD73** | 925.4 ± 970.3 | 1618.6 ± 2006.5 | 1462.5 ± 664.8 | 889.2 ± 870.7 | 550.9 ± 480.2 |
| **VTCN** | 636.3 ± 500.6 | 804.2 ± 1344.3 | 639.3 ± 376.3 | 826.9 ± 666.2 | 934.2 ± 316.8 |
| **APRIL** | 375.6 ± 238.8 | 593.1 ± 687.1 | 312.0 ± 279.6 | 516.1 ± 201.7 | 307.8 ± 326.2 |
| **VISTA** | 45.7 ± 111.4 | 40.1 ± 52.1 | 75.6 ± 92.8 | 44.2 ± 31.4 | 34.5 ± 7.0 |
| **B7-H6** | 71.9 ± 42.2 | 117.8 ± 81.2 | 94.9 ± 95.9 | 76.5 ± 62.9 | 90.0 ± 23.1 |
| **Granzyme B** | 8.5 ± 12.5 | 21.2 ± 78.8 | 16.3 ± 24.7 | 18.1 ± 29.3 | 15.0 ± 9.2 |
| **E-cadherin** | 84510.3 ± 107437.5 | 76994.1 ± 119834.5 | 66625.7 ± 49591.6 | 71242.5 ± 36336.6 | 37225.6 ± 27713.1 |
| **Gal-1** | 10600.3 ± 5209.7 | 13106.9 ± 4293.6 | 9771.1 ± 2966.0 | 12342.3 ± 3163.5 | 15252.1 ± 4659.9 |
| **Gal-3** | 4866.3 ± 2031.9 | 6014.1 ± 3126.8 | 5737.1 ± 3650.0 | 5652.8 ± 1760.6 | 5679.5 ± 1701.5 |
| **Granulysin** | 842.0 ± 445.1 | 1028.5 ± 626.0 | 653.8 ± 331.8 | 979.2 ± 492.1 | 642.4 ± 174.3 |
| **IDO-1** | 161.6 ± 159.1 | 177.8 ± 188.5 | 388.2 ± 583.2 | 113.3 ± 44.5 | 65.4 ± 31.6 |
| **MIC-A** | 19.3 ± 13.2 | 23.5 ± 22.3 | 17.2 ± 10.9 | 28.9 ± 7.2 | 20.7 ± 5.0 |
| **MIC-B** | 350.3 ± 167.3 | 439.1 ± 447.6 | 375.7 ± 367.5 | 313.6 ± 237.5 | 425.6 ± 229.1 |
| **BAFF** | 1804.1 ± 1676.5 | 2161.8 ± 2414.3 | 916.9 ± 716.4 | 1777.5 ± 1848.3 | 703.4 ± 95.6 |
| **OX-40** | 118.1 ± 64.2 | 152.7 ± 117.9 | 195.5 ± 278.7 | 204.5 ± 67.2 | 109.5 ± 36.5 |
| **PVR** | 64263.7 ± 42220.4 | 89382.4 ± 45399.3 | 79491.1 ± 43022.6 | 108353.6 ± 56969.0 | 74224.4 ± 38519.0 |
| **Perforin** | 4715.3 ± 2648.0 | 5120.1 ± 3033.6 | 4863.6 ± 2235.7 | 6052.6 ± 4226.8 | 10889.7 ± 3741.8 |
|  | **1C** | | | |  |
| **Analyte** | **R** | **PR** | **SD** | **HPR** |  |
| **FGF-2** | 46.3 ± 37.2 | 43.6 ± 33.8 | 54.1 ± 32.0 | 34.4 ± 11.2 |  |
| **Eotaxin** | 107.6 ± 78.1 | 95.4 ± 58.1 | 104.1 ± 41.7 | 56.8 ± 22.8 |  |
| **G-CSF** | 96.4 ± 208.7 | 44.6 ± 44.9 | 30.0 ± 15.2 | 11.1 ± 9.8 |  |
| **GM-CSF** | 7.1 ± 8.6 | 7.5 ± 7.8 | 8.5 ± 8.9 | 8.3 ± 7.9 |  |
| **FKN** | **53.6 ± 26.8** | **51.2 ± 39.3** | **79.6 ± 74.2** | **45.7 ± 17.4** |  |
| **IFNα** | 25.6 ± 19.3 | 31.2 ± 53.4 | 22.0 ± 15.4 | 19.2 ± 3.1 |  |
| **IFNγ** | 7.2 ± 6.7 | 5.8 ± 3.2 | 23.9 ± 54.4 | 3.6 ± 3.4 |  |
| **IL-10** | 13.6 ± 27.2 | 23.9 ± 58.1 | 17.0 ± 24.3 | 10.0 ± 11.3 |  |
| **IL12p70** | 7.1 ± 14.3 | 4.4 ± 11.3 | 3.2 ± 1.7 | 1.6 ± 1.5 |  |
| **IL-15** | 0.6 ± 1.4 | 0.9 ± 1.3 | 1.2 ± 1.7 | 0.7 ± 1.2 |  |
| **sCD40L** | 630.8 ± 2651.4 | 406.1 ± 1089.8 | 210.3 ± 298.2 | 62.3 ± 26.5 |  |
| **IL-17** | 2.1 ± 2.4 | 2.4 ± 2.8 | 6.4 ± 12.5 | 0.8 ± 0.8 |  |
| **IL1RA** | 16.6 ± 12.3 | 43.0 ± 76.6 | 20.3 ± 17.0 | 34.7 ± 20.3 |  |
| **IL-1β** | 0.7 ± 1.4 | 0.9 ± 1.2 | 3.1 ± 5.8 | 0.3 ± 0.3 |  |
| **IL-2** | 1.4 ± 2.2 | 1.2 ± 1.7 | 0.7 ± 0.6 | 0.6 ± 0.3 |  |
| **IL-3** | 0.6 ± 0.7 | 0.9 ± 0.7 | 0.9 ± 0.7 | 0.5 ± 0.4 |  |
| **IL-5** | 0.9 ± 0.4 | 0.9 ± 1.1 | 0.5 ± 0.2 | 0.5 ± 0.1 |  |
| **IL-6** | 5.8 ± 10.1 | 6.4 ± 11.6 | 1.8 ± 4.0 | 1.6 ± 1.5 |  |
| **IL-7** | 3.2 ± 2.8 | 3.1 ± 6.5 | 1.5 ± 1.7 | 1.2 ± 1.6 |  |
| **IL-8** | 14.5 ± 18.5 | 28.5 ± 52.0 | 16.4 ± 14.0 | 4.8 ± 2.6 |  |
| **IP-10** | 951.3 ± 695.9 | 1318.2 ± 1322.4 | 1205.7 ± 1424.6 | 1295.6 ± 601.4 |  |
| **MCP-1** | 277.7 ± 164.6 | 444.7 ± 353.3 | 301.5 ± 301.6 | 439.8 ± 132.8 |  |
| **MIP-1a** | 2.6 ± 2.5 | 2.5 ± 2.6 | 4.1 ± 3.6 | 4.6 ± 5.8 |  |
| **MIP-1b** | 29.2 ± 24.6 | 31.5 ± 23.0 | 28.0 ± 16.3 | 34.7 ± 15.6 |  |
| **TNFα** | 13.6 ± 6.6 | 18.5 ± 14.2 | 19.8 ± 20.8 | 31.6 ± 21.3 |  |
| **VEGF** | 61.4 ± 102.9 | 52.8 ± 94.2 | 64.6 ± 121.2 | 36.4 ± 9.7 |  |
| **BTLA** | 1512.5 ± 1311.6 | 1887.3 ± 1406.5 | 1727.2 ± 872.0 | 1106.2 ± 444.5 |  |
| **CD27** | 1952.8 ± 1640.7 | 2074.5 ± 1291.8 | 1364.8 ± 1150.5 | 2680.3 ± 934.4 |  |
| **CD28** | 4502.8 ± 4786.3 | 5540.8 ± 5638.6 | 9454.0 ± 16205.5 | 2932.1 ± 2203.5 |  |
| **TIM3** | 2239.3 ± 1597.2 | 3269.4 ± 1535.6 | 2296.9 ± 886.9 | 3342.3 ± 1117.9 |  |
| **HVEM** | 1367.0 ± 1358.0 | 1759.0 ± 824.7 | 1473.8 ± 1387.6 | 1628.4 ± 297.2 |  |
| **CD40** | 516.2 ± 224.0 | 713.6 ± 287.2 | 683.3 ± 325.7 | 571.9 ± 290.2 |  |
| **GITR** | 335.0 ± 484.5 | 446.9 ± 435.6 | 583.6 ± 420.3 | 223.6 ± 216.9 |  |
| **LAG3** | 42439.5 ± 28754.3 | 53144.6 ± 26778.9 | 70465.7 ± 23905.2 | 32345.2 ± 16039.0 |  |
| **TLR2** | 3464.8 ± 3093.0 | 4152.5 ± 2841.1 | 5835.0 ± 6389.2 | 2468.2 ± 983.2 |  |
| **GITRL** | 1105.4 ± 799.2 | 1257.2 ± 790.9 | 1203.9 ± 617.3 | 799.1 ± 333.3 |  |
| **PD-1** | 3511.8 ± 2420.6 | 2910.8 ± 1648.4 | 5376.9 ± 6193.8 | 2382.4 ± 468.6 |  |
| **CTLA-4** | 99.4 ± 118.2 | 116.2 ± 108.5 | 114.1 ± 93.7 | 104.8 ± 59.8 |  |
| **CD80** | 330.8 ± 317.3 | 390.5 ± 282.3 | 377.7 ± 136.9 | 226.3 ± 116.8 |  |
| **CD86** | 1942.2 ± 1707.9 | 2269.2 ± 1461.9 | 2591.6 ± 1263.1 | 1294.2 ± 827.9 |  |
| **PD-L1** | 315.6 ± 319.1 | 388.0 ± 231.3 | 406.8 ± 176.5 | 216.4 ± 112.1 |  |
| **ICOS** | 4055.5 ± 3047.5 | 4917.4 ± 3371.1 | 5312.4 ± 2475.4 | 2615.8 ± 1157.9 |  |
| **Arginase1** | 666.2 ± 1457.7 | 455.1 ± 724.9 | 1359.2 ± 2865.8 | 165.6 ± 223.3 |  |
| **ICOSL** | 3468.4 ± 2419.7 | 3671.3 ± 2167.1 | 3369.9 ± 1241.2 | 4479.9 ± 1407.5 |  |
| **CD276** | 994.9 ± 571.2 | 1215.5 ± 568.8 | 886.5 ± 462.3 | 983.6 ± 542.5 |  |
| **CD73** | 820.7 ± 920.3 | 6576.9 ± 33384.5 | 2082.8 ± 2427.7 | 548.3 ± 775.5 |  |
| **VTCN** | 630.4 ± 483.0 | 887.3 ± 1541.2 | 638.5 ± 396.0 | 681.2 ± 424.9 |  |
| **APRIL** | 623.6 ± 914.0 | 632.4 ± 616.3 | 446.5 ± 272.8 | 435.9 ± 208.6 |  |
| **VISTA** | 46.0 ± 81.6 | 64.8 ± 130.9 | 79.7 ± 104.0 | 43.8 ± 34.6 |  |
| **B7-H6** | 72.2 ± 55.4 | 128.6 ± 91.8 | 114.5 ± 125.4 | 83.0 ± 67.9 |  |
| **Granzyme B** | 15.4 ± 29.3 | 33.7 ± 59.6 | 21.8 ± 41.3 | 9.7 ± 10.5 |  |
| **E-cadherin** | 79841.8 ± 102820.9 | 73015.9 ± 89655.7 | 69720.7 ± 45131.2 | 67288.1 ± 47311.8 |  |
| **Gal-1** | 10904.1 ± 6870.5 | 13362.2 ± 4296.6 | 10511.1 ± 3456.1 | 11630.4 ± 3088.4 |  |
| **Gal-3** | 5417.5 ± 2732.0 | 6175.3 ± 2685.1 | 6828.8 ± 4745.8 | 5152.1 ± 1429.9 |  |
| **Granulysin** | 1055.6 ± 983.2 | 1350.7 ± 904.6 | 908.7 ± 584.5 | 944.6 ± 324.4 |  |
| **IDO-1** | 322.8 ± 584.5 | 214.2 ± 243.7 | 494.7 ± 772.7 | 142.2 ± 65.6 |  |
| **MIC-A** | 20.4 ± 14.3 | 29.0 ± 38.7 | 18.7 ± 11.7 | 31.9 ± 15.9 |  |
| **MIC-B** | 330.5 ± 123.6 | 544.3 ± 825.6 | 379.2 ± 323.4 | 346.8 ± 294.0 |  |
| **BAFF** | 2319.9 ± 3341.6 | 3046.8 ± 2749.2 | 2074.1 ± 2398.8 | 2157.1 ± 1061.0 |  |
| **OX-40** | 180.1 ± 151.4 | 186.5 ± 83.0 | 272.9 ± 432.5 | 313.5 ± 159.1 |  |
| **PVR** | 66329.9 ± 39760.1 | 93820.7 ± 54476.9 | 81003.1 ± 47797.7 | 95843.9 ± 37313.2 |  |
| **Perforin** | 5521.4 ± 3508.9 | 6926.9 ± 4620.1 | 5881.6 ± 4255.8 | 6732.8 ± 5220.3 |  |
