# Supplementary material for: Plasma fractalkine contributes to systemic myeloid diversity and PD‐L1/PD‐1 blockade in lung cancer
Source: EMBO Rep. 2023 Jun 27;24(8):e55884. doi: 10.15252/embr.202255884 (PMC10398648; doi:10.15252/embr.202255884)
Supplement: Supplementary file 5 [file EMBR-24-e55884-s004.pdf]

Plasma fractalkine contributes to systemic myeloid diversity and PD-L1/PD-1 blockade in lung cancer

Ana Bocanegra, Gonzalo Fernandez-Hinojal, Daniel Ajona, Ester Blanco, Miren Zuazo, Maider Garnica, Luisa Chocarro, Elvira Alfaro-Arnedo, Sergio Piñeiro, Pilar Morente, Leticia Fernandez, Ana Remirez, Miriam Echaide, Maite Martinez, Idoia Morilla, Beatriz Tavira, Alejandra Roncero, Carolina Gotera, Alfonso Ventura, Nerea Recalde, Jose G Pichel, Juan Lasarte, Luis Montuenga, Ruth Vera, Ruben Pio, David Escors, and Grazyna Kochan
DOI: 10.15252/embr.202255884

Corresponding author(s): David Escors (descorsm@navarra.es) , David Escors (descorsm@navarra.es), Ana Bocanegra (ai.bocanegra.gondan@navarra.es), Grazyna Kochan (grazyna.kochan@navarra.es)

Review Timeline:

Submission Date:	1st Aug 22
Editorial Decision:	10th Aug 22
Revision Received:	17th Feb 23
Editorial Decision:	12th May 23
Revision Received:	17th May 23
Accepted:	2nd Jun 23

Editor: Martina Rembold

Transaction Report: This manuscript was transferred to EMBO reports following peer review at EMBO Molecular Medicine.

(Note: With the exception of the correction of typographical or spelling errors that could be a source of ambiguity, letters and reports are not edited. Depending on transfer agreements, referee reports obtained elsewhere may or may not be included in this compilation. Referee reports are anonymous unless the Referee chooses to sign their reports.)

Referee #1 (Remarks for Author):

Kochan et al, in their study entitled "*Potent clinical predictive and systemic adjuvant therapeutic value of plasma*
*fractalkine in PD-L1/PD-1 blockade immunotherapy for lung cancer" *tried to build a hypothesis upon the impact of FKN on control of NSCLC at various platform.
The concept is justified as there is dire need of improving the outcome of cancer immunotherapy in general. However considering their various levels of the analysis and model which is mislinked, it is still hard to believe that FKN is the candidate host factor which can help host in controlling tumors. Although their animal experiments provide some hint of that but that is also not black and white in readouts. I have following concerns with the experiments and analysis which need attention by the authors before considering this paper further for publication
*1. *In figure 1, author have analyzed only monocytic cell population in the patients but failed to correlate the frequencies of CD163/CCL18+ TAM which are main population of macrophages involved in the tumor development. They analyzed cd66b+ populations in various cohorts.
*2. *Author should use R for responder; it is not clear how they have selected hyper responsive patients in their analysis. What was the pathological score of staging of these patients?
*3. *Is there any explanation of sharp increase in the cd66+ population in PR, and is this correlated stage wise ?
*4. *In their supplementary table 2- there are many cytokines and growth factors which show similar patterns like FKN , why author did not follow on that. It could be the FKN might be working in presence of these Th1 effectors for observed phenotype. This need clarification by the author
*5. *In Fig 2I , did author also check the metabolic activity of 3LL cells also which may contribute to their growth
*6. *In fig 3 , did author tried to see the cross talk of FKN with MCSF for immune reconstitution on various cells. In my view it would be more advantageous to see the impact on FKN on phenotypical polarization of macrophages. Also their CD4/8 ratio also does not fit as per hematological criteria
*7. *In their cell depletion strategies, they have not shown the depletion efficacy which is important. Why author did not deplete

macrophages population in the animals while they are mentioning / clarifying the impact of macrophages on tumor development.
*8. *In figure 5 , they have not analyzed CD14+/CCL18+/CD163+ populations because in lung these cells are decisive for tumor development
*9. *And more importantly their study could not build a clear hypothesis on the impact on FKN on the tumor development.
On the basis of their unclear and big jargon analysis, I do not recommend / support this study for publication as it stands as of now

Referee #2 (Remarks for Author):

The authors candidate fractalkine (FKN) as a prognostic/predictive indicator in lung cancer. A main claim of the article is "synergy between FKN and PD-L1/PD-1 blockade immunotherapy". The main findings of the article are presented in Fig. 2D,E, and show impact of circulating FKN on progression-free and overall survival of lung cancer patients under PD-L1/PD-1 blockade immunotherapy. This was determined by a ROC analysis (Fig.2C). This is inherently prone to overfitting and an independent validation cohort of PD-L1/PD-1 blockade-treated lung cancer patients is mandatory. The TCGA database data, besides regarding mRNA and not protein levels, originate from untreated patients. Hence, besides being very weak, they are irrelevant for the purpose of the article.
An additional point is that while FKN effects on experimental models are convincing, they are not novel in the literature.
Data on synergy between FKN and PD-L1/PD-1 blockade immunotherapy are simply not there (see the detailed comments below). An analysis of FKN impact on control patients not treated with PD-L1/PD-1 blockade is entirely missing.
Some confusion on circulating vs cancer-synthesized FKN, and between direct growth inhibition Vs immune mediated anti-cancer responses add to the lack of focus of the experimental rationale(s) followed. See detailed comments below.

FINDINGS
Abstract
High-dimensional systemic immune profiling was performed in a discovery cohort of 112 non-small cell lung cancer patients undergoing PD-L1/PD-1 blockade immunotherapy. High-throughput profiling of soluble factors in plasma identified fractalkine (FKN), a chemokine involved in immune chemotaxis and adhesion, as a biomarker of myeloid cell diversity in human patients and in murine models, which was found significantly increased in objective responders.
Secreted FKN inhibited adenocarcinoma and squamous cell carcinoma growth in vivo through a prominent contribution of systemic effector NK cells, enhanced tumor infiltration with immunostimulatory immune cells and inhibition of MDSCs within tumors.
synergy between FKN and PD-L1/PD-1 blockade immunotherapy was found in murine lung cancer models refractory to anti-PD-L1/anti-PD-1 treatment.
recombinant FKN and tumor expressed-FKN were efficacious in delaying tumor growth in vivo

Introduction
tumors that overexpress GM-CSF, G-CSF, M-CSF and IL-6 ultimately lead to systemic expansion of immunosuppressive MDSCs that counteract anti-tumor T cells and NK cells.

patients with increased HLA-DR+ monocytes and decreased neutrophils in peripheral blood show good response rates to ICB therapies In contrast, patients with increased HLA-DR+ monocytes and decreased neutrophils in peripheral blood show good response rates to ICB therapies

high-throughput characterization of plasma factor signatures was carried out to associate specific profiles with systemic immune cell diversity and objective responses. Plasma fractalkine (FKN, CX3CL1) concentration was the best candidate for a biomarker of response, which correlated with systemic myeloid diversity and response to PD-L1/PD-1 blockade.

FKN is a chemokine that belongs to the CX3C family. This protein is mainly expressed by endothelial cells and signals through its canonical receptor CX3CR1, and αvβ3 and α4β1 integrins (19). It is produced as a membrane-bound protein that mediates cellular adhesion. A soluble form released by metalloproteases such as ADAM-10 and ADAM-17 (20, 21) regulates immune chemotaxis. FKN has been reported to elicit anti-tumor responses by recruiting T cells, dendritic cells (DCs) and natural killer (NK) cells to the tumor microenvironment ...pro-oncogenic role of FKN is attributed to its ability to promote tumor migration and invasion

Major comments on experimental findings
- In a 2007 review "Experiments to assess its role on NK cell biology showed a strong correlation between CX3CL1 and anti-tumour responses. Hence, when CX3CL1-expressing lymphoma cell lines were injected into mice, tumour growth was suppressed due to the recruitment of NK cells that controlled the tumour via production of IFN□ and perforin (Lavergne et al., 2003). Similar experiments utilizing a lung carcinoma cell line also concluded that CX3CL1 is required for NK cell recruitment at the site of tumour growth, leading thus to its eradication (Guo et al., 2003). ..Furthermore, mice deficient for CX3CR1 were substantially repressed in their ability to recruit NK cells in the lung both in the presence of cancer and under homeostatic conditions.
Therefore, CX3CL1 and its receptor CX3CR1 regulate the migration of NK cells to tumour sites as well as their effector function.

Results
"First, we evaluated classical variables based on standard clinical quantification of immune cell populations in peripheral blood of patients undergoing PD-L1/PD-1 blockade immunotherapy. These analyses were performed on a well-characterized cohort of 112 NSCLC patients (TableS1) and described in previous studies elsewhere (2, 4, 11)."
"No significant differences were found between responders and non-responders after having analyzed routine clinical parameters, such as NLR, monocyte/lymphocyte ratio and platelet/lymphocyte ratio, or absolute monocyte and neutrophil counts (Fig. S1), thus indicating their limited ability to predict response."
- Fig. 1. may be misleading. Panels G and J show the correlation between Diversity index and %monocytes in CD11b cells or CD66b cells. The correlation coefficients are very small, consistent with the broad dot-plot distributions. Highly significant P values indicate the likelyhood that THAT THE SMALL COEFFICIENT IS TRUE, not that the correlation between the two parameters is high.

"Objective responders had significantly ($p<0.0001$) elevated baseline percentages of monocytes within CD11b+ cells (39.86% {plus minus} 10.85; Mean ; Mean {plus minus} SD) compared to patients with stable disease, progressors (16.41% {plus minus} 14.14; ; Mean {plus minus} SD) and hyperprogressors. In agreement with other studies (17, 40), it had predictive value in our discovery cohort.

...These data confirmed that objective responders showed a systemic myeloid signature dominated by monocytic CD14+ HLA-DR+ cells accompanied with low abundance of granulocytic CD66b+ myeloid cells (neutrophils and G-MDSCs), while patients with stable disease, progressors and hyperprogressors showed differential myeloid profiles (Fig. S3)."

- These findings are a confirmation of data the authors recognize to be published in the literature. The real issue thus is how novel the presented findings are, rather than simply incremental versus the published literature.

While most of the quantified plasma soluble analytes showed increased levels in patients who rendered no objective clinical responses to anti PD-1/PD-L1 immunotherapy, some of them, such as GM-CSF, G-CSF and IL12p70, were elevated in a sample of responders before treatment, but only FKN concentrations reached statistical significance (p=0.0037)

- Fig. S4: some of the P values indicated are unfitting with real data distributions, see Galectin I and III as examples. Most of the comparisons presented are very weak.

"murine adenocarcinoma 3LL cells were engineered to constitutively overexpress GM-CSF, G-CSF, M-CSF or FKN (Fig. S5). We found that only GM-CSF and FKN expression significantly but inversely altered the in vitro growth rate of 3LL cells (Fig. 2H). While FKN was a very strong inhibitor of cell growth, GM-CSF was a significant enhancer of 3LL proliferation"

- Although a useful control, proliferation of cancer cells expressing FKN or GM-CSF does not fit the model proposed in the article, whereby modulation of the immune system/immune responses affect cancer growh. The same applies to transcriptomic analysis of lung cancer samples, whereby endogenous FKN production is assessed. Administration of recombinant FKN to xenotransplanted mice follows the same, alternative rationale.

- The origin of FKN production, whether by cancer cells or by normal cells of the host/patient should be determined. Are the lung tumors of the tested case series FKN positive? Do FKN cancer cell levels correlate with ICI responses?

"FKN synergizes with anti-PD1 immunotherapy
Our clinical data associated plasma FKN concentrations superior than 55 pg/mL with objective responses to PD-L1/PD-1 blockade..."

- There is no synergy of FKN with anti-PD1. The effect seen on tumor volume is caused by FKN.

"Mice were then treated with anti-PD-1
immunotherapy and tumor growth was monitored at both flanks. Interestingly, the growth of 3LL tumors was delayed, and comparable to 3LL-FKN tumors in the same mice (Fig. 4G). These results demonstrated abscopal effects of FKN associated with anti-PD-1 blockade. Hence, FKN acts in synergy with PD-1 blockade systemically, as well as promoting immune infiltration of tumors with NK cells."

- FKN inhibits 3LL tumor growth. Circulating FKN is likely to inhibit the non-expressing 3LL. This is not an abscopal effect, but direct inhibition by FKN.

"Figure 4 C-E"
- There is no difference between immune cell infiltrates in FKN alone vs FKN plus anti-PD1 antibodies

Referee #3 (Remarks for Author):

Bocanegra et al investigate the biology of soluble fractakine (FKN, aka CX3CL1) in NSCLC. The overall message of the paper is that the level of systemic FKN might be a useful marker to stratify NSCLC patients for treatment with anti-PD-1 and that soluble FKN skews the peripheral/systemic immune landscape to one that favors immune-mediated clearance of NSCLC. The authors identity FKN as a target for investigation by in depth analysis of PBMCs and plasma cytokines of 112 NSCLC patients treated with anti-PD-1. Responding patients could be distinguished in this patient cohort by use of a circulating myeloid cell diversity index, by elevated monocytes and by lower levels of CD11b+, CD66b+ cells. This clinical data is interesting and the authors provide ROC curves that support the sensitivity and specificity of these clinical measures. To extend the studies the authors examine plasma from the patient cohort for 65 cytokines or other relevant soluble factors. FKN is identified as a factor that is associated with high myeloid cell diversity, elevated monocytes and increased survival. To study FKN 3LL cells are engineered to stably express FKN, which results in slower growth in vitro and in vivo and is associated with reduced peripheral granulocyte numbers compared to PBMCs from mice bearing 3LL-WT tumors. The authors show that anti-PD-1 therapy is more effective in mice bearing 3LL-FKN tumors than 3LL-WT tumors and they provide some evidence that this effect is mediated via systemic effects of FKN, potentially via NK cells. It is an interesting manuscript with many strengths. However, the comments below should be addressed to improve the impact of the main message.
Comments:
1. The authors indicate that in the human samples FKN expression is associated with increased myeloid cell diversity. However, in Figure 3F in the 3LL tumors expressing various cytokines there is almost no difference in myeloid diversity between 3LL-WT and 3LL-FKN tumors? The comparison to expression of other cytokines (MCSF, GM-CSF, G-CSF) is appreciated and interesting in its own right but overall this graph does not support the initial claim. Can the authors comment on this? Flow from splenocytes from the 3LL-WT and 3LL-FKN at minimum should be shown.

2. Identification of FKN as the soluble factor to focus on is explained in the text well. However, the data shown in Figure 2A on FKN plasma levels in the different patient cohorts is underwhelming. This challenge is exacerbated by Fig 2F. In 2F LUAD data from the TCGA is shown with high FKN expression associated with slightly better outcome than low FKN expression. These concerns should be discussed.

3. There is no histology shown of any NSCLC, human or murine. It would be very helpful to show which cells in a non-engineered tumor express FKN. This could also be performed by

scRNA-seq, although IHC or ISH would be preferred.

4. Figure 3A is interesting. Inhibition of Cx3CR1, the receptor for FKN, increases 3LL growth in vivo, this is supportive of the concept that the authors are pitching. However injection of 100 µg/kg of rFKN has a modest negative effect on tumor growth. A concern with the rFKN experiment is that there is no control (e.g., a distinct non-relevant protein) and it is only tested at 1 dose, which is not shown to yield statistically significant results. I realize this is potentially an expensive experiment given that the rFKN was purchased from abcam, but showing an effect at higher doses of rFKN or doing repeated doses along with more robust controls would greatly strengthen the conclusions.

5. Are Fig 3B and 3C separate experiments? What is the metric for survival? Does FKN in the 3LL-FKN tumors have a direct effect on tumor cells, meaning is tumor cell proliferation decreased, is apoptosis of tumor cells elevated? Further if IHC is performed are T cells or NK cells associated with tumor cell nests more in 3LL-FKN tumors compared to the others?

6. Are Fig 4A and 4B the same experiment or distinct studies? Figure 4 shows Flow from the tumor with FKN + anti-PD-1 or FKN alone resulting in elevated CD45, NK or CD4. However, Figure S8 shows no difference between the groups in neutrophils or other subsets. This seems to be inconsistent with the data showing changes in granulocytes in the periphery (Fig. 3G). Are neutrophils in Fig S8 and granulocytes in Fig. 3G defined differently? If so tumor associated granulocytes should be evaluated.

7. Figure 3F/G is a very interesting setup. The data suggest that priming the mouse with a tumor (in this case on the left side) increases the efficacy of anti-PD-1 on the second tumor implanted (on the right side). The right side tumor in the WT/WT mice appears to be responding robustly to anti-PD-1, please comment. Treatment of existing 3LL-WT tumors with rFKN and anti-PD-1 is a potential strategy to enhance the impact of this figure.

8. Figure 6 adds minimally to the overall impact of the study.

Minor:
Annotation for the different colors/groups is needed for Figure S4.

1st Editorial Decision 10th Aug 2022

Dear Dr. Escors

Thank you for the transfer of your research manuscript to our journal. We have now carefully read your study and the transferred referee reports.

We appreciate that you provide evidence that FKN/CXCL3 is a potential biomarker to predict the therapeutic success of anti-PD-1 therapy in NSCLC patients. Moreover, you propose that FKN synergizes with anti-PD-1 therapy to reduce tumor growth.

However, we also note that the effect of FKN/CX3CL1 on tumor growth and NK cell recruitment is known. We note that both referees are not convinced that your current data set provides strong evidence for a synergistic effect of FKN and PD-L1/PD-1 blockade. In your feedback to my recent e-mail you indicated that you can address the referee comments in full and that you can also provide further experiments on the synergistic effects of FKN and PD-1 blockade. You did not provide a more detailed point-by-point response, but we have now decided to invite you to revise your study for EMBO reports, but I want to emphasize once more that further data providing clear evidence that FKN acts synergistically with PD-1 blockade will be required for potential publication in EMBO Reports.

I am also happy to discuss these revisions further, also in a Zoom call if you wish. Generally, as the outcome of these experiments are not predictable, I suggest to remain in contact regarding the progress of this work.

Taken together, we thus invite you to revise your manuscript with the understanding that the referee concerns (as detailed above and in their reports) must be fully addressed and their suggestions taken on board. Please address all referee concerns in a complete point-by-point response. Acceptance of the manuscript will depend on a positive outcome of a second round of review. It is EMBO reports policy to allow a single round of revision only and acceptance or rejection of the manuscript will therefore depend on the completeness of your responses included in the next, final version of the manuscript.

We realize that it is difficult to revise to a specific deadline. In the interest of protecting the conceptual advance provided by the work, we recommend a revision within 3 months (November 10). Please discuss the revision progress ahead of this time with the editor if you require more time to complete the revisions.

*****IMPORTANT NOTE:
We perform an initial quality control of all revised manuscripts before re-review. Your manuscript will FAIL this control and the handling will be DELAYED if the following APPLIES:

1) A data availability section providing access to data deposited in public databases is missing. If you have not deposited any data, please add a sentence to the data availability section that explains that.

2) Your manuscript contains statistics and error bars based on n=2. Please use scatter blots in these cases. No statistics should be calculated if n=2.

When submitting your revised manuscript, please carefully review the instructions that follow below. Failure to include requested items will delay the evaluation of your revision.*****

When submitting your revised manuscript, we will require:

1) a .docx formatted version of the manuscript text (including legends for main figures, EV figures and tables). Please make sure that the changes are highlighted to be clearly visible.

2) individual production quality figure files as .eps, .tif, .jpg (one file per figure).
Please download our Figure Preparation Guidelines (figure preparation pdf) from our Author Guidelines pages https://www.embopress.org/page/journal/14693178/authorguide for more info on how to prepare your figures.

3) a .docx formatted letter INCLUDING the reviewers' reports and your detailed point-by-point responses to their comments. As part of the EMBO Press transparent editorial process, the point-by-point response is part of the Review Process File (RPF), which will be published alongside your paper.

4) a complete author checklist, which you can download from our author guidelines (). Please insert information in the checklist that is also reflected in the manuscript. The completed author checklist will also be part of the RPF.

5) Please note that all corresponding authors are required to supply an ORCID ID for their name upon submission of a revised manuscript (). Please find instructions on how to link your ORCID ID to your account in our manuscript tracking system in our

Author guidelines
()

6) We replaced Supplementary Information with Expanded View (EV) Figures and Tables that are collapsible/expandable online. A maximum of 5 EV Figures can be typeset. EV Figures should be cited as 'Figure EV1, Figure EV2" etc... in the text and their respective legends should be included in the main text after the legends of regular figures.

- For the figures that you do NOT wish to display as Expanded View figures, they should be bundled together with their legends in a single PDF file called *Appendix*, which should start with a short Table of Content. Appendix figures should be referred to in the main text as: "Appendix Figure S1, Appendix Figure S2" etc. See detailed instructions regarding expanded view here:

- Additional Tables/Datasets should be labeled and referred to as Table EV1, Dataset EV1, etc. Legends have to be provided in a separate tab in case of .xls files. Alternatively, the legend can be supplied as a separate text file (README) and zipped together with the Table/Dataset file.

7) Before submitting your revision, all primary datasets (and computer code, where appropriate) produced in this study need to be deposited in an appropriate public database (see <
https://www.embopress.org/page/journal/14693178/authorguide#dataavailability>).

Please remember to provide a reviewer password if the datasets are not yet public.

The accession numbers and database should be listed in a formal "Data Availability " section (placed after Materials & Method) that follows the model below (see also < https://www.embopress.org/page/journal/14693178/authorguide#dataavailability>). Please note that the Data Availability Section is restricted to new primary data that are part of this study.

Data availability

The datasets (and computer code) produced in this study are available in the following databases:

- RNA-Seq data: Gene Expression Omnibus GSE46843 (https://www.ncbi.nlm.nih.gov/geo/query/acc.cgi?acc=GSE46843)
- [data type]: [name of the resource] [accession number/identifier/doi] ([URL or identifiers.org/DATABASE:ACCESSION])

*** Note - All links should resolve to a page where the data can be accessed. ***

8) The journal requires a statement specifying whether or not authors have competing interests (defined as all potential or actual interests that could be perceived to influence the presentation or interpretation of an article). In case of competing interests, this must be specified in your disclosure statement. Further information: https://www.embopress.org/competing-interests

9) Figure legends and data quantification:
The following points must be specified in each figure legend:

- the name of the statistical test used to generate error bars and P values,
- the number (n) of independent experiments (please specify technical or biological replicates) underlying each data point,
- the nature of the bars and error bars (s.d., s.e.m.)
- If the data are obtained from n {less than or equal to} 2, use scatter blots showing the individual data points.

Discussion of statistical methodology can be reported in the materials and methods section, but figure legends should contain a basic description of n, P and the test applied.

See also the guidelines for figure legend preparation:
https://www.embopress.org/page/journal/14693178/authorguide#figureformat

- Please also include scale bars in all microscopy images.

10) We would also encourage you to include the source data for figure panels that show essential data. Numerical data should be provided as individual .xls or .csv files (including a tab describing the data). For blots or microscopy, uncropped images should be submitted (using a zip archive if multiple images need to be supplied for one panel). Additional information on source data and instruction on how to label the files are available .

11) Our journal encourages inclusion of *data citations in the reference list* to directly cite datasets that were re-used and obtained from public databases. Data citations in the article text are distinct from normal bibliographical citations and should

directly link to the database records from which the data can be accessed. In the main text, data citations are formatted as follows: "Data ref: Smith et al, 2001" or "Data ref: NCBI Sequence Read Archive PRJNA342805, 2017". In the Reference list, data citations must be labeled with "[DATASET]". A data reference must provide the database name, accession number/identifiers and a resolvable link to the landing page from which the data can be accessed at the end of the reference. Further instructions are available at .

12) As part of the EMBO publication's Transparent Editorial Process, EMBO reports publishes online a Review Process File to accompany accepted manuscripts. This File will be published in conjunction with your paper and will include the referee reports, your point-by-point response and all pertinent correspondence relating to the manuscript.

You are able to opt out of this by letting the editorial office know (emboreports@embo.org). If you do opt out, the Review Process File link will point to the following statement: "No Review Process File is available with this article, as the authors have chosen not to make the review process public in this case."

We would also welcome the submission of cover suggestions, or motifs to be used by our Graphics Illustrator in designing a cover.

I look forward to seeing a revised form of your manuscript when it is ready and please let me know if you have questions or comments regarding the revision.

Please use this link to submit your revision: https://embor.msubmit.net/cgi-bin/main.plex

Yours sincerely,

Martina Rembold, PhD
Senior Editor
EMBO reports

FIRST REVIEWER:

1. "The main findings of the article are presented in Fig. 2D,E, and show impact of circulating FKN on progression-free and overall survival of lung cancer patients under PD-L1/PD-1 blockade immunotherapy. This was determined by a ROC analysis (Fig.2C). This is inherently prone to overfitting and an independent validation cohort of PD-L1/PD-1 blockade-treated lung cancer patients is mandatory".

We appreciate the Reviewer´s comment and suggestion. ROC analysis is the standard statistical tool to evaluate the predictive value of a given variable, and as such, it depends on the input data. Therefore, we have obtained data from an independent validation cohort of 18 NSCLC patients treated with the combination of chemo-immunotherapy (pembrolizumab + platinum-based chemotherapy) as first line treatment, of whom 13 are responders and 5 were non-responders. Processing, quantitation and analysis was carried out independently by personnel from another institution, and they have been added to the manuscript as authors. We have added this information in page 4, line 143 and now in the new Figure EV3A as follows:

* In page 4, line 144:" A validation cohort independently confirmed our results in a limited sample of patients (Fig EV3A)."

2. "The TCGA database data, besides regarding mRNA and not protein levels, originate from untreated patients. Hence, besides being very weak, they are irrelevant for the purpose of the article".

We appreciate the Reviewer´s comment. It is true that part of the data from the TCGA is weak in the context of the overall paper, for example, survival. This data has been removed from the article. However, it contains data on mRNA expression between malignant and non-malignant tissue. In light of suggestions from the other Reviewer, we kept this data but grouped in a supplementary figure (Figure EV3) with data on fractalkine expression by histochemistry. This part of the data complements our results because it helps to address the question on the origin of FKN (tumor or non-tumor) as the Referee has pointed out in another concern.

To clarify these concerns we have re-written the manuscript, highlighting the following points.

- In page 5, from line 158: "FKN is known to be produced by healthy tissue. To find out whether lung cancer cells could also be a source of FKN in lung cancer patients, we quantified in vitro FKN secretion by a collection of 10 human lung adenocarcinoma (LUAD) and lung squamous carcinoma (LUSC) cell lines. FKN production was very heterogeneous, and only two cell lines out of 10 clearly overexpressed FKN (Fig 2A). This was in agreement with our clinical observations, since a minor percentage of NSCLC patients showed plasma FKN concentrations above the cut-off value (Fig 1I). Due to the unavailability of tumor tissue biopsies from our discovery cohort, we evaluated FKN expression in a limited sample of biopsies from another cohort of LUAD patients. Double immunostainings were

performed to detect the lung tumor marker Pan-Cytokeratin (PanCK) together with FKN in tumor and adjacent healthy tissue (Fig EV3B and C). FKN expression was inversely correlated with PanCK expression within both adjacent non-tumor and LUAD tissues (Pearson's correlation: r=-0.9016, p<0.001 (adjacent non-tumor tissues) and r=-0.8518; p<0.002 (LUAD tissues) (Fig EV3D and E). In vitro results together with biopsy data were indicators that FKN was poorly expressed in tumor tissue in the majority of lung cancer patients. This data was confirmed by evaluating FKN transcriptomic expression in clinical samples from lung cancer patients registered in The Cancer Genome Atlas (TCGA) database (Fig EV3F)."

- In page 7, line 241: "Then, we evaluated whether these changes had a reflection on tumor infiltration. FKN expression significantly enhanced immune infiltration of the tumor (Fig 4I), mainly with CD4 T and NK cells (Fig 4J and K). No significant differences were observed for the rest of the main cell types (Fig EV6). As we lacked biopsy data from our cohort of patients, we complemented the results in mouse models with analyses of transcriptional data from lung adenocarcinoma samples in the TCGA database. The Tumor Immune Estimation Resource tool (TIMER2.0) was used to estimate immune infiltration and perform correlations with FKN transcription (Li et al, 2020). Positive correlations of FKN expression were found with infiltration by CD8 (Rho = 0.222, p=6.28e-07), CD4 (Rho=0.185, p=3.66e-05), NK (Rho=0.244, p=4.07e-08) cells, neutrophils (Rho=0.211, p=2.39e-06) and monocytes (Rho=0.263, p=2.96e-09) (Fig EV7A). In contrast, a negative correlation with MDSC infiltration was observed (Rho=-0.269, p=1.21e-09). Correlation of FKN with PD-L1 tumor expresssion was also confirmed, as PD-L1 is a clinically-approved biomarker of response to PD-L1/PD-1 blockade (Doroshow et al, 2021) (Fig EV7B). Increased FKN transcription also correlated with PD-1 expression (Fig EV7C)."

3. "An additional point is that while FKN effects on experimental models are convincing, they are not novel in the literature."

In our manuscript we are using lung adenocarcinoma cancer models not used before for the study of FKN, including the combination with anti-PD1. We are also using novel, genetically-modified lung cancer cell lines to further our understanding on FKN biology and systemic effects. The in vivo experiments are the most complete carried out so far, which also include myeloid profiling in peripheral blood and within the tumor. These experiments follow the data from our clinical discovery cohort on patients undergoing PD-L1/PD-1 blockade therapy, which is also a novel observation of importance for the clinician and biomedical researcher (see point 4 below).

To further clarify the reader the novelty of our study, we have also changed the title of the manuscript as : "Plasma fractalkine contributes to systemic myeloid diversity and PD-L1/PD-1 blockade in lung cancer"

4. “Data on synergy between FKN and PD-L1/PD-1 blockade immunotherapy are simply not there (see the detailed comments below)”.

We apologise to the Reviewer for lack of clarity in our manuscirpt in this regard. Letting aside the issue of murine versus human contexts, the adenocarcinoma models that we are using in mice are completely refractory to PD-1 blockade. Thus, the Reviewer´s comment is relevant in the sense that no synergy can take place between PD-1 and FKN, but just because anti-PD-1 treatment does not work in our mouse models.

And this is the key point in our manuscript. In the new Figure 4A we have included data on tumor growth, finding that this is the case. Anti-PD-1 treatment does not inhibit the growth of unmodified tumors. However, these tumors are sensitized to a further line of anti-PD-1 treatment in the presence of secreted FKN. Therefore, FKN and anti-PD-1 treatments indeed do not synergise. FKN sensitizes tumors to anti-PD-1 treatments. It is true that in mouse models, it does not translate into significant survival. We accept this observation, and we have included this point also in our manuscript.

As such, we do really need to clarify these issues to the reader: In page 15:

- **In page 6, line 220:** “**FKN sensitizes lung adenocarcinoma tumors intrinsically resistant to anti-PD1 immunotherapies.** Our clinical data associated elevated plasma FKN concentrations with objective clinical responses to PD-L1/PD-1 blockade. Therefore, we investigated whether FKN contributed to ICB efficacy in the murine 3LL lung adenocarcinoma model, which is intrinsically refractory to these immunotherapies. Indeed, while unmodified 3LL tumors were fully resistant to PD-1 blockade in vivo, FKN expression sensitized tumors to PD-1 blockade. This sensitization led to a highly significant ($P<0.001$) reduction in tumor size (Fig 4A) although with limited impact on survival (Fig 4B and C). It has nevertheless to be noted that in our mouse models FKN expression is a major driving force of anti-tumor activities, causing a very highly significant delay ($p<0.0001$) in tumor growth (Fig 4B) associated to a very significant ($p=0.001$) increase in median survival from 20 to 35 days (Fig 4C).”
- **In page 9, line 342:** Importantly, we demonstrated that FKN expression in adenocarcinoma cells refractory to ICB therapy (Ajona D, 2020) could sensitize tumors to PD-1 blockade. This sensitization further retarded the growth of tumors, although in our mouse models this did not translate into a benefit in survival. Nevertheless, it has to be mentioned that FKN exerted very potent anti-tumor activities, and improvements in survival would likely require blockers even stronger than classical anti-PD-1 antibodies.”

5. “An analysis of FKN impact on control patients not treated with PD-L1/PD-1 blockade is entirely missing.”

The Reviewer is right. This group is usually included to confirm or discard whether the biomarker has prognostic value (independently of the treatment) rather than predictive value

(specific for immunotherapy). This issue is usually solved in related work (including our own, for example in Zuazo et al. EMBO Mol Med) by comparing the time from diagnosis to enrolment in immunotherapy (which follows progression from chemotherapy) for the same patient cohort that later received immunotherapy. Difference in time between FKN high and FKN low patients is related to the prognostic power of the biomarker under study. To comply with this point, we have included this new data in the manuscript as follows:

- In page 4, line 147 To find out whether FKN had prognostic value in our cohort for treatments other than immunotherapies, we analyzed the time elapsed between diagnosis and progression in our discovery cohort during which patients were undergoing chemotherapy only. No significant differences were found, thus discarding that plasma FKN had significant prognostic value in our cohort (Fig 1L).

6. " Fig. 1. may be misleading. Panels G and J show the correlation between Diversity index and %monocytes in CD11b cells or CD66b cells. The correlation coefficients are very small, consistent with the broad dot-plot distributions. Highly significant P values indicate the likelyhood that THAT THE SMALL COEFFICIENT IS TRUE, not that the correlation between the two parameters is high."

We apologise to the Reviewer if he/she thought that Figure 1 may be misleading. But this is not the case: we are providing all the data, plotting all the analyses and presenting the data in the figure to the reader.

The probability in a Spearman´s correlation test corresponds the probability that two given variables do not correlate. It does not mean that the correlation between two parameters is high, but significant or non-significant from a statistical point of view. This probability is calculated using the correlation coefficient, together with the degrees of freedom of the samples. The probabilities in our correlation studies were significant, which is an objective conclusion. The statistical power was sufficiently good to rely on this conclusion. We may have weak correlations which as the Reviewer points, are given by Rho, but nevertheless significant due to the given sample size. These correlations and data dispersion are typical of data from these kind of discovery clinical studies, and such data can we found in the literature.

Neutrophilia is a very well-known prognostic marker of progression, which would ultimately lead to a loss of diversity of myeloid subsets in peripheral blood. No surprises there. Monocytes are also good prognostic immune cells, no surprise there either. In our manuscript we have quantified the diversity of phenotypes from high-dimensional data in PD-L1/PD-1 blockade immunotherapies in lung cancer patients, which is novel.

7. "These findings are a confirmation of data the authors recognize to be published in the literature. The real issue thus is how novel the presented findings are, rather than simply incremental versus the published literature".

We appreciate the sincerity of the Reviewer´s opinion, but we have been working in cancer immunotherapy for a long time, and in all our publish work we try to be fair and cite all the relevant literature. Hence, I think that we also have a good criterion on assessing novelty of data compared to the published literature. Our goal is to publish the data from our studies fairly.

The role of FKN is ambivalent in the literature, with both pro- and anti-oncogenic roles. This may reflect that FKN is affecting different signalling pathways and mechanisms. This is a research line that we are currently pursuing. We have mentioned above some of the novel points in our study, which are significant and of high interest for biomedical researchers and physicians. The novelty is adequately summarized now by the title of our manuscript.

Our data heavily relies on clinical responses to PD-L1/PD-1 blockade immunotherapy in lung cancer, where we found a significant relationship between FKN plasma concentration, immune cell diversity and clinical responses. We also provide evidence that FKN can be mobilized as a therapeutic agent in patients. The follow-up studies in mouse models are mandatory for these type of studies.

8. "Fig. S4: some of the P values indicated are unfitting with real data distributions, see Galectin I and III as examples. Most of the comparisons presented are very weak."

We would like to clarify to the Reviewer that we are not assuming a Gaussian distribution, and we apologise if we gave that impression. No assumption was made for the distribution of the variables, and we used hence non-parametric tests. These tests lose sensitivity, but at least they gain robustness. Nevertheless, we agree that this is a limitation of our study, more to do with sample availability than anything else.

It is true that the figures may not be worth showing, especially because they deviate the attention from the main cytokine FKN in our paper. Consequently, no leads were followed for most of the cytokines. We have removed the explicit figures and mentions to other cytokines. Nevertheless, we are keeping all quantitative data in a Table. We may have lost some interesting targets that would surely arise in an enlarged cohort sample, no doubt. But we have done this to be consistent with the conclusions in our study.

9. "Although a useful control, proliferation of cancer cells expressing FKN or GM-CSF does not fit the model proposed in the article, whereby modulation of the immune system/immune responses affect cancer growh. The same applies to transcriptomic analysis of lung cancer samples, whereby endogenous FKN production is assessed. Administration of recombinant FKN to xenotransplanted mice follows the same, alternative rationale".

The data from modified cell lines is clear, straight-forward and complements the findings in the experiments. Evidently, we had to evaluate the in vitro effects of FKN over cancer cells. FKN can decrease cancer cell growth, but also contributes to activation of anti-tumor responses in vivo. Biomolecules and cytokines possess more than one function. The goal of

our study was to find as much valuable information as possible, which we in fact did. As a consequence, our manuscript is one of the most comprehensive in the subject.

The data obtained from these modified cell lines is significant and of high interest for the biomedical community. They constitute valuable experimental models. Transcriptomic analyses of lung cancer samples complements the question on the origin of FKN that has been answered in point 2.

Administration of recombinant FKN supports the relationship of plasma FKN with anti-tumor responses. This experiment is straightforward and of high relevance for the biomedical community. This was further stressed as follows:

- **In page 9, line 321:** "It is important to note that FKN possessed systemic anti-tumor activities without the need to be expressed within the tumor microenvironment. For example, FKN expressed by a tumor can exert inhibitory activities over a distal tumor that does not express FKN. This is important, because it was previously thought that FKN expressed by tumors could exert anti-tumor effects through recruitment of T cells, DCs and NK cells to the tumor microenvironment (Hyakudomi et al., 2008; Kee et al., 2013; Lavergne et al., 2003; Ohta et al., 2005; Park et al., 2012; Tang et al., 2007; Xin et al., 2005; Yamauchi et al., 2021; Yamauchi et al., 2020). Nevertheless, we found that recombinant FKN was very unstable in vivo. The systemic administration of recombinant FKN as a therapeutic approach might require experimental strategies to effectively deliver cytokines to the TME in a targeted way while improving their stability."

10. " The origin of FKN production, whether by cancer cells or by normal cells of the host/patient should be determined. Are the lung tumors of the tested case series FKN positive? Do FKN cancer cell levels correlate with ICI responses?"

We agree with the Reviewer and we recognize the relevance of that information. Unfortunately, tumor biopsies from our patient cohort are not available. Most lung cancer samples do not provide sufficient tissue biopsy, but they rely mostly on cytologies or circulating tumor cells subjected to NGS for detection of oncogenes and mutations for targeted therapy.

To clarify the issue concerning FKN source, we have incorporated immunocytochemistry analyses of FKN together with the lung tumor cell marker pan-cytokeratin in a sample cohort of 10 biopsies of lung cancer patients from a collection belonging to another institution. Hence, these collaborators have been incorporated as authors, and the data data is included in Figure EV3, as well as in the manuscript as follows:

- **In page 5, from line 158:** "FKN is known to be produced by healthy tissue. To find out whether lung cancer cells could also be a source of FKN in lung cancer patients, we quantified in vitro FKN secretion by a collection of 10 human lung adenocarcinoma (LUAD) and lung squamous carcinoma (LUSC) cell lines. FKN production was very heterogeneous, and only two cell lines out of 10 clearly overexpressed FKN (Fig 2A). This was in agreement with our clinical observations,

since a minor percentage of NSCLC patients showed plasma FKN concentrations above the cut-off value (Fig 1I). Due to the unavailability of tumor tissue biopsies from our discovery cohort, we evaluated FKN expression in a limited sample of biopsies from another cohort of LUAD patients. Double immunostainings were performed to detect the lung tumor marker Pan-Cytokeratin (PanCK) together with FKN in tumor and adjacent healthy tissue (Fig EV3B and C). FKN expression was inversely correlated with PanCK expression within both adjacent non-tumor and LUAD tissues (Pearson's correlation: r=-0.9016, p<0.001 (adjacent non-tumor tissues) and r=-0.8518; p<0.002 (LUAD tissues) (Fig EV3D and E). In vitro results together with biopsy data were indicators that FKN was poorly expressed in tumor tissue in the majority of lung cancer patients. This data was confirmed by evaluating FKN transcriptomic expression in clinical samples from lung cancer patients registered in The Cancer Genome Atlas (TCGA) database (Fig EV3F)."

- **In page 7, line 241:** "Then, we evaluated whether these changes had a reflection on tumor infiltration. FKN expression significantly enhanced immune infiltration of the tumor (Fig 4I), mainly with CD4 T and NK cells (Fig 4J and K). No significant differences were observed for the rest of the main cell types (Fig EV6). As we lacked biopsy data from our cohort of patients, we complemented the results in mouse models with analyses of transcriptional data from lung adenocarcinoma samples in the TCGA database. The Tumor Immune Estimation Resource tool (TIMER2.0) was used to estimate immune infiltration and perform correlations with FKN transcription (Li et al, 2020). Positive correlations of FKN expression were found with infiltration by CD8 (Rho = 0.222, p=6.28e-07), CD4 (Rho=0.185, p=3.66e-05), NK (Rho=0.244, p=4.07e-08) cells, neutrophils (Rho=0.211, p=2.39e-06) and monocytes (Rho=0.263, p=2.96e-09) (Fig EV7A). In contrast, a negative correlation with MDSC infiltration was observed (Rho=-0.269, p=1.21e-09). Correlation of FKN with PD-L1 tumor expresssion was also confirmed, as PD-L1 is a clinically-approved biomarker of response to PD-L1/PD-1 blockade (Doroshow et al, 2021) (Fig EV7B). Increased FKN transcription also correlated with PD-1 expression (Fig EV7C)."

11. "There is no synergy of FKN with anti-PD1. The effect seen on tumor volume is caused by FKN."

We apologise to the Reviewer for lack of clarity in our manuscirpt in this regard. Letting aside the issue of murine versus human contexts, the adenocarcinoma models that we are using in mice <u>are completely refractory to PD-1 blockade</u>. Thus, the Reviewer´s comment is relevant in the sense that no synergy can take place between PD-1 and FKN, but just because anti-PD-1 treatment does not work in our mouse models.

And this is the key point in our manuscript. In the new Figure 4A we have included data on tumor growth, finding that this is the case. Anti-PD-1 treatment does not inhibit the growth of unmodified tumors. However, these tumors are sensitized to a further line of anti-PD-1 treatment in the presence of secreted FKN. Therefore, FKN and anti-PD-1 treatments indeed do not synergise. FKN sensitizes tumors to anti-PD-1 treatments. It is true that in mouse

models, it does not translate into significant survival. We accept this observation, and we have included this point also in our manuscript.

As such, we do really need to clarify these issues to the reader: In page 15:

- **In page 6, line 220:** "**FKN sensitizes lung adenocarcinoma tumors intrinsically resistant to anti-PD1 immunotherapies.** Our clinical data associated elevated plasma FKN concentrations with objective clinical responses to PD-L1/PD-1 blockade. Therefore, we investigated whether FKN contributed to ICB efficacy in the murine 3LL lung adenocarcinoma model, which is intrinsically refractory to these immunotherapies. Indeed, while unmodified 3LL tumors were fully resistant to PD-1 blockade in vivo, FKN expression sensitized tumors to PD-1 blockade. This sensitization led to a highly significant ($P<0.001$) reduction in tumor size (Fig 4A) although with limited impact on survival (Fig 4B and C). It has nevertheless to be noted that in our mouse models FKN expression is a major driving force of anti-tumor activities, causing a very highly significant delay ($p<0.0001$) in tumor growth (Fig 4B) associated to a very significant ($p=0.001$) increase in median survival from 20 to 35 days (Fig 4C)."
- **In page 9, line 342:** Importantly, we demonstrated that FKN expression in adenocarcinoma cells refractory to ICB therapy (Ajona D, 2020) could sensitize tumors to PD-1 blockade. This sensitization further retarded the growth of tumors, although in our mouse models this did not translate into a benefit in survival. Nevertheless, it has to be mentioned that FKN exerted very potent anti-tumor activities, and improvements in survival would likely require blockers even stronger than classical anti-PD-1 antibodies."

12. "FKN inhibits 3LL tumor growth. Circulating FKN is likely to inhibit the non-expressing 3LL. This is not an abscopal effect, but direct inhibition by FKN"

The Reviewer is right, and we have amended this as follows:

- **In page 7, line 255**: "Most of the experiments in mouse models were carried out by expressing FKN from cancer cells. It could be argued that anti-tumor effects were restricted to the FKN-expressing tumors, while the clinical data (Fig 1G) and treatments with recombinant FKN (Fig 2B-C) strongly suggested systemic anti-tumor activities by circulating FKN. Therefore, to demonstrate systemic activities for secreted FKN, 3LL-FKN cells were subcutaneously implanted in mice on the left flank (inoculation side), followed by injection of unmodified 3LL cells on the right flank (target tumor side) one week later (Fig 5A). Mice were then treated with anti-PD-1 immunotherapy and tumor growth was monitored at both flanks. Interestingly, the growth of 3LL tumors was delayed and comparable to 3LL-FKN tumors in the same mice (Fig 5B). These results demonstrated that secreted FKN associated with anti-PD-1 blockade could delay the growth of an immunotherapy-resistant distal tumor that did not express FKN. Therefore, FKN secreted from modified tumor cells showed efficacious systemic therapeutic activities."

SECOND REFEREE

The second Referee is rather positive to our study, recognising the interest in it, especially the clinical data supported by ROC analyses. Referee 2 raises a number of questions to improve the impact of the main message. We truly appreciate the comments and suggestions for improvement raised by the second Reviewer, which we have addressed as follows:

1. "The authors indicate that in the human samples FKN expression is associated with increased myeloid cell diversity. However, in Figure 3F in the 3LL tumors expressing various cytokines there is almost no difference in myeloid diversity between 3LL-WT and 3LL-FKN tumors? The comparison to expression of other cytokines (MCSF, GM-CSF, G-CSF) is appreciated and interesting in its own right but overall this graph does not support the initial claim. Can the authors comment on this? Flow from splenocytes from the 3LL-WT and 3LL-FKN at minimum should be shown."

We appreciate the Reviewer from pointing this issue out to us, and also to give us a hint to overcome this problem in mouse models by assessing analyses in splenocytes.

It is true that that in Figure 3F (Fig. 3C in updated version), overall, no appartent differences were found in cell diversity between 3LL-WT and 3LL-FKN tumors. But we have to consider the intrinsic differences between the two systems. The course of lung cancer in human patients is rather different from the mouse models, and prolonged times may be needed to find more striking differences in peripheral blood. However, as the reviewer said, we indeed found better results in splenocytes, for example. Both in peripheral blood and supported by the data in splenocytes, we detected a significant expansion of monocytic cells and a significant decrease in granulocytes when FKN was expressed. This data agree with the data in human peripheral blood. Indeed, in mice harbouring tumors overproducing FKN we do detect significantly lower percentages of circulating granulocytes (as shown in Fig. 3C), as well as lower percentages of granulocytes and increased frequency of monocytes in the spleen (shown in Fig. 4). In humans, the systemic expansion of the corresponding granulocytic subset (CD66b) dominated the loss of cell diversity in the myeloid compartment. The rest of controls in Fig. 3 confirm that FKN expression does not impair cell diversity.

Nevertheless, the Reviewer is right in that we need to clarify these points to the reader. We provide flow cytometry data from splenocytes of 3LL-WT and 3LL-FKN mice in Fig. 4. We have added all this information in the manuscript as follows:

- In page 6, line 207: "Profiling of circulating immune cells was performed 15 days after tumor engraftment, once significant differences in tumor volume were observed (Fig 3B). In our murine experimental models, the composition of myeloid and lymphoid compartments in tumor-bearing mice was apparently similar to that of healthy control mice with the exception of GM-CSF and G-CSF producing tumors (Fig 3C). When looked in detail and in agreement with our clinical data, mice with 3LL-FKN tumors had elevated circulating DC, macrophages and monocytes, accompanied with a significant decrease in the percentage of Ly6G+ granulocytes

(12.25% ± 2.36) compared to control mice with 3LL tumors (29.2% ± 9.9) (Fig 3D). In contrast, mice bearing 3LL/GM-CSF tumors showed profiles comparable to that from human hyperprogressors, characterized by an exponential acceleration of tumor growth (p<0.0001) tumor growth, shortened survival (median survival of 14 days vs 17 days of wild type control, p=0.009) and overwhelming numbers of MDSCs (58.52% ± 9.17 of Ly6G+ cells)."

- **In page 6, line 232**: "Then, we studied whether the combination of FKN expression and PD-1 blockade had a further impact on the composition of systemic immune cells. These studies were carried out at a time point with significant differences in tumor volumes (15 days after tumor engraftment). We characterized the relative composition in major immune cell types within CD45+ cells in spleens, to detect systemic changes in our mouse models. No major impact of the anti-PD-1 immunotherapy was observed on top of FKN at least within myeloid cell types (Fig EV5). In agreement with our clinical data and previous experiments, FKN expression increased the relative percentage of classical and non-classical monocytes (Fig 4D and E), and a reduction in neutrophils (Fig 4F). Anti-PD-1, however, increased the percentage of PD-1+ T cells (Fig 4G), but not of NK cells (Fig 4H). Then, we evaluated whether these changes had a reflection on tumor infiltration. FKN expression significantly enhanced immune infiltration of the tumor (Fig 4I), mainly with CD4 T and NK cells (Fig 4J and K). No significant differences were observed for the rest of the main cell types (Fig EV6)."
- **In page 8, line 311:"** Hence, in our discovery cohort FKN plasma concentration showed good predictive value, which was confirmed in a validation cohort. In agreement with the clinical data, mice engrafted with FKN-producing tumors showed increased OS with elevated percentages of monocytes and reduced Ly6G+ granulocytes in peripheral blood and in spleen."

2. "Identification of FKN as the soluble factor to focus on is explained in the text well. However, the data shown in Figure 2A on FKN plasma levels in the different patient cohorts is underwhelming. This challenge is exacerbated by Fig 2F. In 2F LUAD data from the TCGA is shown with high FKN expression associated with slightly better outcome than low FKN expression. These concerns should be discussed."

We agree with the Reviewer that these concerns should be discussed, mainly due to the limited availability of plasma samples. We have also included data from a validation cohort, performed by an independent research team from another institution, whom have been added as authors of the manuscript. The validation data confirms our initial claim.

We admit that, both the differences in the plasma FKN concentrations and the improvement on overall survival according to transcriptomic expression of FKN are subtle, although still statistically significant. This data was used for confirmation purposes taking advantage of open access biopsy data. However, due to concerns from Reviewer 1 (that data was not really relevant for the purpose of the paper), this data has been removed. These concerns were discussed in the manuscript as follows:

- **In page 4, line 139:** "Therefore, a panel of 65 cytokines, chemokines and soluble immune checkpoints was evaluated. While most of the quantified analytes showed

increased levels in patients who rendered no objective clinical responses to anti PD-1/PD-L1 immunotherapy (Table EV2), only FKN concentrations were significantly (p=0.0037) elevated in a sample of responders before treatment (Fig 1G, Table EV2). A validation cohort independently confirmed our results in a limited sample of patients (Fig EV3A)..

3. "There is no histology shown of any NSCLC, human or murine. It would be very helpful to show which cells in a non-engineered tumor express FKN. This could also be performed by scRNA-seq, although IHC or ISH would be preferred".

We appreciate the Reviewer´s suggestion, which would improve our manuscript. Hence, we include in our revised manuscript IHC analyses of FKN expression together with pan-cytokeratin in a sample cohort of 10 biopsies of lung cancer patients. This data is included in Figure EV3, as well as in the manuscript as follows:

- **In page 5, line 158:** "FKN is known to be produced by healthy tissue. To find out whether lung cancer cells could also be a source of FKN in lung cancer patients, we quantified in vitro FKN secretion by a collection of 10 human lung adenocarcinoma (LUAD) and lung squamous carcinoma (LUSC) cell lines. FKN production was very heterogeneous, and only two cell lines out of 10 clearly overexpressed FKN (Fig 2A). This was in agreement with our clinical observations, since a minor percentage of NSCLC patients showed plasma FKN concentrations above the cut-off value (Fig 1I). Due to the unavailability of tumor tissue biopsies from our discovery cohort, we evaluated FKN expression in a limited sample of biopsies from another cohort of LUAD patients. Double immunostainings were performed to detect the lung tumor marker Pan-Cytokeratin (PanCK) together with FKN in tumor and adjacent healthy tissue (Fig EV3B and C). FKN expression was inversely correlated with PanCK expression within both adjacent non-tumor and LUAD tissues (Pearson's correlation: r=-0.9016, p<0.001 (adjacent non-tumor tissues) and r=-0.8518; p<0.002 (LUAD tissues) (Fig EV3D and E). In vitro results together with biopsy data were indicators that FKN was poorly expressed in tumor tissue in the majority of lung cancer patients. This data was confirmed by evaluating FKN transcriptomic expression in clinical samples from lung cancer patients registered in The Cancer Genome Atlas (TCGA) database (Fig EV3F)."

4. "Figure 3A is interesting. Inhibition of Cx3CR1, the receptor for FKN, increases 3LL growth in vivo, this is supportive of the concept that the authors are pitching. However injection of 100 µg/kg of rFKN has a modest negative effect on tumor growth. A concern with the rFKN experiment is that there is no control (e.g., a distinct non-relevant protein) and it is only tested at 1 dose, which is not shown to yield statistically significant results. I realize this is potentially an expensive experiment given that the rFKN was purchased from abcam, but showing an effect at higher doses of rFKN or doing repeated doses along with more robust controls would greatly strengthen the conclusions".

We truly appreciate the comments from Reviewer 2 and, as requested, we added a control group of mice treated with anti-trinitrophenol IgG2a antibody as an irrelevant protein control in addition to the saline buffered-treated group previously shown as vehicle control. This protein control did not affect tumor growth as shown now in new data included in Figure 2.

Concerning the administration of recombinant FKN, the effects are not that modest when assessed early during tumor progression (around day 10), as shown now in the new Figure 2C. The reduction in tumor size is highly significant. However, this reduction does not translate later on in a significant improvement in survival, regretfully. We found that this lack of efficacy in the "long-term" was due to the limited half-life of recombinant FKN. We repeated the experiment modifying the experimental schedule as suggested by the reviewer 2. However, we were also unable to demonstrate a further potentiation of tumor growth delay in the long term after the intraperitoneal administration of 3 doses of 100 μg/kg of rFKN. We have been producing recombinant FKN to reduce costs, and found that the protein was unstable. This suggests that for direct administration of FKN, the molecule needs further engineering to improve its in vivo stability. We ended up with the setup of the optimal experimental conditions as economically unfeasible. Hence, we stressed in the manuscript the effects of in vivo targeting the signalling axis CX3CR1-CX3CL1 with the inhibitor AZD8797, which rendered significantly higher tumor volumes 20 days after tumor implantation. In addition we added the new data with the protein control as suggested by the Reviewer.

Therefore, to comply with the Reviewer´s request, we have included the following:

- **In page 5, line 174:** "To find out whether FKN could trigger anti-tumor mechanisms in NSCLC patients, the FKN-CX3CR1 signaling axis was systemically targeted in a mouse model of lung adenocarcinoma. In agreement with our hypothesis, the pharmacological inhibition of CX3CR1 by the administration of the allosteric non-competitive FKN antagonist AZD8797 accelerated tumor progression ($p<0.05$) (Fig 2B). This result was supported by systemic administration of recombinant FKN, which delayed tumor growth, compared to the null effect of a non-relevant control protein (IgG) (Fig 2C). To overcome the challenging therapeutic schedule with recombinant FKN in mouse models, we overexpressed FKN directly from cancer cell lines, as observed for some human lung adenocarcinoma cell lines such as H358 and SW900 (Fig 2A). To that end, murine adenocarcinoma cell lines Lacun3 (Bleau et al, 2014) and 3LL were engineered to express FKN (Fig EV4A)."
- **In page 9, line 321:** "It is important to note that FKN possessed systemic anti-tumor activities without the need to be expressed within the tumor microenvironment. For example, FKN expressed by a tumor can exert inhibitory activities over a distal tumor that does not express FKN. This is important, because it was previously thought that FKN expressed by tumors could exert anti-tumor effects through recruitment of T cells, DCs and NK cells to the tumor microenvironment (Hyakudomi et al., 2008; Kee et al., 2013; Lavergne et al., 2003; Ohta et al., 2005; Park et al., 2012; Tang et al., 2007; Xin et al., 2005; Yamauchi et al., 2021; Yamauchi et al., 2020). Nevertheless, we found that recombinant FKN was very unstable in vivo. The systemic

administration of recombinant FKN as a therapeutic approach might require experimental strategies to effectively deliver cytokines to the TME in a targeted way while improving their stability.”

5. “Are Fig 3B and 3C separate experiments? What is the metric for survival? Does FKN in the 3LL-FKN tumors have a direct effect on tumor cells, meaning is tumor cell proliferation decreased, is apoptosis of tumor cells elevated? Further if IHC is performed are T cells or NK cells associated with tumor cell nests more in 3LL-FKN tumors compared to the others?”

These are not separate experiments. Apart from the immune-mediated antitumor mechanisms elicited by FKN *in vivo*, it has a direct effect on tumor cells *in vitro* by decreasing tumor cell proliferation, as shown in Fig. 2 and in Figure EV4. To further clarify this point, we provide additional data that demonstrate the direct inhibitory effect of the recombinant protein *in vitro* on tumor cell growth rate, as well as apoptosis induction of 3LL cells by FKN, as follows:

- **In page 5, line 190:** “In contrast, FKN was a strong inhibitor of tumor cell growth rate and an inductor of apoptosis of cancer cells (Fig 2E and EV4B-D).”
- In Fig. EV4 we provide further data on propidium iodide uptake by apoptotic unmodified 3LL cells cultured in 3LL-WT (control) or 3LL-FKN conditioned medium, as well as the real time cell growth monitoring of unmodified and FKN-producing 3LL cell lines cultured with 1 μg/mL of recombinant FKN.

6. “Are Fig 4A and 4B the same experiment or distinct studies? Figure 4 shows Flow from the tumor with FKN + anti-PD-1 or FKN alone resulting in elevated CD45, NK or CD4. However, Figure S8 shows no difference between the groups in neutrophils or other subsets. This seems to be inconsistent with the data showing changes in granulocytes in the periphery (Fig. 3G). Are neutrophils in Fig S8 and granulocytes in Fig. 3G defined differently? If so tumor associated granulocytes should be evaluated.”

Fig. 4A and 4B correspond to the same experiment. The reviewer asks whether tumor associated neutrophils in Fig. S8 and circulating granulocytes in Fig. 3G (Fig. 3D in uptaded version) are defined differently. Both populations are defined as the percentage of $CD45^{+}$ $Ly6G^{+}$ cells. We apologise to the Reviewer if these points were not explained sufficiently clear in our manuscript. To make it clearer to the reader, we have modified the caption of the figues to specify the phenotypic definition of tumor associated neutrophils, as follows:

- “Tumor infiltrating immune cells in mice transplanted with 3LL cells expressing FKN and its combination with PD-1 blockade. The graphs represent percentages of the indicated infiltrating immune cell types as quantified by flow cytometry, in tumors excised from mice inoculated with the indicated cell lines (parental cell line, WT; 3LL cells expressing FKN, FKN) with or without PD-1 blockade. Neutrophils (Ly6G), CD8, B cells (CD19), Ly6C+ monocytes, DCs (CD11c) and macrophages (F4/80) were quantified at day 14 after tumor inoculation. Data are shown as the mean of the percentage within total leukocytes (CD45+) ± SD (n=8 mice). Relevant

statistical comparisons are shown within the graphs, evaluated by ANOVA and Tukey´s pairwise comparisons. ns, non-significant differences.”

It is true that there were no significant differences in tumor-infiltrating neutrophils between groups of mice in our specific experimental systems. However, this do not have to be necessary in conflict with an increase in peripheral blood and splenic neutrophils. Nevertheless, as we did not find significant differences in neutrophil tumor infiltration, we decided to show the data in a supplementary figure.

7. “Figure 3F/G is a very interesting setup. The data suggest that priming the mouse with a tumor (in this case on the left side) increases the efficacy of anti-PD-1 on the second tumor implanted (on the right side). The right side tumor in the WT/WT mice appears to be responding robustly to anti-PD-1, please comment. Treatment of existing 3LL-WT tumors with rFKN and anti-PD-1 is a potential strategy to enhance the impact of this figure”.

We truly appreciate the comment from Reviewer 2. As we commented before, we found a major issue with the stability of recombinant FKN. Indeed, we tried the experiment several times, but FKN was found to be unstable in plasma as recombinant protein. At this instance, our group is starting to test modifications of FKN to improve its in vivo stability so we can purify and carry out the experiments suggested by the Reviewer. However, as this work may take a long time, we will rather use it for a follow-up study.

Nevertheless, and to fulfil the requests from Revierwer 1 as well, we have clarified these points by suppressing the word ‘synergy’ by highlighting instead that ‘FKN sensitizes lung tumors to anti-PD-1 immunotherapy’ mainly based on the results shown in Fig. 4A. To make it clearer to the reader, we have modified the manuscript as follows:

- **In page 7, line 263:** “These results demonstrated that secreted FKN and anti-PD-1 blockade could delay the growth of a distal tumor that did not express FKN, demonstrating systemic therapeutic activities over distal sites.

8. “Figure 6 adds minimally to the overall impact of the study”.

We initially considered that the differential analysis of FKN contribution in lung adenocarcinoma and lung squamous carcinoma models is of importance for the clinician and biomedical researcher. Nevertheless, we agree with the Reviewer 2 that this figure could even be detrimental for the focus of our manuscript. Hence, we decided to suppress Fig. 6, as well as all the references to it throughout the manuscript.

9. “Minor: Annotation for the different colors/groups is needed for Figure S4.”

This figure is not longer present in the revised manuscript.

Dear David,

Thank you for the submission of your revised manuscript to EMBO reports. I apologize again for the delay in handling your manuscript. Unfortunately, the first referee was not available anymore but we have received a very positive report from the second referee (now #1), who supports publication of your study in EMBO reports without further revision and after checking your response to referee 2, I have decided to move forward with the publication of your manuscript.

Browsing through the manuscript, I noticed a few editorial things that we need before we can proceed with the official acceptance of your study.

- You note in the Materials and Methods section for the validation cohort: "As a validation cohort, 18 patients diagnosed with locally advanced or metastatic NSCLC treated with the combination of chemo-immunotherapy ..." without specifying whether written informed consent was obtained from these patients as well. Please check and add this information.

- Please update the 'Conflict of interest' paragraph to our new 'Disclosure and competing interests statement'. For more information see
https://www.embopress.org/page/journal/14693178/authorguide#conflictsofinterest

- Regarding the Author Contributions, we now use CRediT to specify the contributions of each author in the journal submission system. CRediT replaces the author contribution section, which therefore needs to be removed from the manuscript text. You can use the free text box in our system if you wish to provide more detailed descriptions. See also guide to authors
https://www.embopress.org/page/journal/14693178/authorguide#authorshipguidelines.

- Please update the references to match the style of EMBO reports. The abbreviation 'et al' should be used if more than 10 authors, i.e., only the first 10 authors are listed. You can download the respective EndNote file from our Guide to Authors
https://endnote.com/style_download/embo-reports/

- Please add callouts to the panels of Fig EV1.

- Please note that we can only typeset up to 5 EV figures. You can either move some figures to the main figures, merge two EV figures or move them to an Appendix (single pdf file, including figures and their legends plus a table of content with page numbers. Nomenclature: Appendix Fig. S#).

- We noticed that most figures have something pasted in the top left corner over the A panel label. Our editorial assistants uploaded new pdf files for these figures. Please look out for this aberration when you revise the manuscript.

- It seems that Fig 4K is labelled as L in the figure.

- During our routine analysis of all figures we noticed that the graphs shown in Figure 5 H and J seem almost identical. Can you please double-check these panels and provide the underlying source data?

- I attach to this email a related manuscript file with comments by our data editors. Please address all comments and upload a revised file with tracked changes with your final manuscript submission.

- Finally, EMBO reports papers are accompanied online by A) a short (1-2 sentences) summary of the findings and their significance, B) 2-3 bullet points highlighting key results and C) a synopsis image that is 550x300-600 pixels large (width x height) in PNG for JPG format. You can either show a model or key data in the synopsis image. Please note that the size is rather small and that text needs to be readable at the final size. Please send us this information along with the revised manuscript.

We look forward to seeing a final version of your manuscript as soon as possible.
Please use this link to submit your revision: https://embor.msubmit.net/cgi-bin/main.plex

Kind regards,

Martina

Martina Rembold, PhD
Senior Editor
EMBO reports

****

Referee #1:

The authors have done a commendable job in addressing the concerns of the prior review. It is an interesting story that is communicated effectively by the manuscript. In my opinion the main point has novelty and potential impact.
I have no other concerns.

Dear Dr. Rembold,

First of all, I would like to thank you for your reply. We are very happy to hear that you have finally considered our manuscript for publication in EMBO Reports. It is a honour for us to publish our results in such a well renowned journal.

On behalf of Dr. David Escors, I am sending you the final version of the manuscript EMBOR-2022-55884V2 to proceed with its official acceptance.

The following changes have been introduced:

- We have specified that written informed consent was obtained from the patients of the validation cohort.

- We have updated the 'Conflict of interest' paragraph to the new 'Disclosure and competing interests statement'.

- We have removed the author contribution section from the manuscript text.

- We have updated the references to match the style of EMBO reports.

- We have added callouts to the panels of Fig EV1.

- We have merged Figure EV3 - EV4 and Figure EV5 - EV6 to separate supplementary figures (now Figure EV3 and Figure EV4), so that there are only 5 EV figures in this final version.

- We have changed Fig 4L label to Fig 4K in the figure.

- We have checked Figure 5 H and J and realized that by mistake we had pasted the same figure in both panels. We have replaced Figure 5J panel with the appropriate figure.

- We have addressed all the comments from data editors with tracked changes and provide a final revised version of the manuscript.

- We provide a synopsis document and graphical abstract for online publication.

- As requested by himself, we have removed one of the co-authors, Dr. Hugo Arasanz, and we have acknowledged his contribution instead.

As Dr. Escors is temporarily unavailable, it will be me, as first author, who will be in charge of all the necessary changes. Please contact me for any further concerns.

Best regards,

Ana Isabel Bocanegra

Dr. David Escors
Navarrabiomed
Pamplona, Navarra 31008
Spain

Dear Dr. Escors,

I am very pleased to accept your manuscript for publication in the next available issue of EMBO reports. Thank you for your contribution to our journal.

At the end of this email I include important information about how to proceed. Please ensure that you take the time to read the information and complete and return the necessary forms to allow us to publish your manuscript as quickly as possible.

As part of the EMBO publication's Transparent Editorial Process, EMBO reports publishes online a Review Process File to accompany accepted manuscripts. As you are aware, this File will be published in conjunction with your paper and will include the referee reports, your point-by-point response and all pertinent correspondence relating to the manuscript.

If you do NOT want this File to be published, please inform the editorial office within 2 days, if you have not done so already, otherwise the File will be published by default [contact: emboreports@embo.org]. If you do opt out, the Review Process File link will point to the following statement: "No Review Process File is available with this article, as the authors have chosen not to make the review process public in this case."

Thank you again for your contribution to EMBO reports and congratulations on a successful publication. Please consider us again in the future for your most exciting work.

Yours sincerely,

Martina Rembold, PhD
Senior Editor
EMBO reports

****

THINGS TO DO NOW:

Please note that you will be contacted by Wiley Author Services to complete licensing and payment information. The required 'Page Charges Authorization Form' is available here: https://www.embopress.org/pb-assets/embo-site/er_apc.pdf - please download and complete the form and return to embopressproduction@wiley.com

EMBO Press participates in many Publish and Read agreements that allow authors to publish Open Access with reduced/no publication charges. Check your eligibility: https://authorservices.wiley.com/author-resources/Journal-Authors/open-access/affiliation-policies-payments/index.html

You will receive proofs by e-mail approximately 2-3 weeks after all relevant files have been sent to our Production Office; you should return your corrections within 2 days of receiving the proofs.

Please inform us if there is likely to be any difficulty in reaching you at the above address at that time. Failure to meet our deadlines may result in a delay of publication, or publication without your corrections.

All further communications concerning your paper should quote reference number EMBOR-2022-55884V3 and be addressed to emboreports@wiley.com.

Should you be planning a Press Release on your article, please get in contact with emboreports@wiley.com as early as possible, in order to coordinate publication and release dates.

EMBO Press Author Checklist

Corresponding Author Name: David Escors	
Journal Submitted to: EMBO Reports	
Manuscript Number: EMBOR-2022-55884V2	

USEFUL LINKS FOR COMPLETING THIS FORM
[The EMBO Journal - Author Guidelines]
[EMBO Reports - Author Guidelines]
[Molecular Systems Biology - Author Guidelines]
[EMBO Molecular Medicine - Author Guidelines]

Reporting Checklist for Life Science Articles (updated January 2022)
This checklist is adapted from Materials Design Analysis Reporting (MDAR) Checklist for Authors. MDAR establishes a minimum set of requirements in transparent reporting in the life sciences (see Statement of Task: 10.31222/osf.io/9sm4x). Please follow the journal's guidelines in preparing your manuscript.
Please note that a copy of this checklist will be published alongside your article.

Abridged guidelines for figures
1. Data
The data shown in figures should satisfy the following conditions:
- the data were obtained and processed according to the field’s best practice and are presented to reflect the results of the experiments in an accurate and unbiased manner.
- ideally, figure panels should include only measurements that are directly comparable to each other and obtained with the same assay.
- plots include clearly labeled error bars for independent experiments and sample sizes. Unless justified, error bars should not be shown for technical replicates.
- if n<5, the individual data points from each experiment should be plotted. Any statistical test employed should be justified.
- Source Data should be included to report the data underlying figures according to the guidelines set out in the authorship guidelines on Data Presentation.

2. Captions
Each figure caption should contain the following information, for each panel where they are relevant:
- a specification of the experimental system investigated (eg cell line, species name).
- the assay(s) and method(s) used to carry out the reported observations and measurements.
- an explicit mention of the biological and chemical entity(ies) that are being measured.
- an explicit mention of the biological and chemical entity(ies) that are altered/varied/perturbed in a controlled manner.
- the exact sample size (n) for each experimental group/condition, given as a number, not a range;
- a description of the sample collection allowing the reader to understand whether the samples represent technical or biological replicates (including how many animals, litters, cultures, etc.).
- a statement of how many times the experiment shown was independently replicated in the laboratory.
- definitions of statistical methods and measures:
  - common tests, such as t-test (please specify whether paired vs. unpaired), simple χ2 tests, Wilcoxon and Mann-Whitney tests, can be unambiguously identified by name only, but more complex techniques should be described in the methods section;
  - are tests one-sided or two-sided?
  - are there adjustments for multiple comparisons?
  - exact statistical test results, e.g., P values = x but not P values < x;
  - definition of ‘center values’ as median or average;
  - definition of error bars as s.d. or s.e.m.

Please complete ALL of the questions below.
Select "Not Applicable" only when the requested information is not relevant for your study.

Materials

Newly Created Materials	Information included in the manuscript?	In which section is the information available? (Reagents and Tools Table, Materials and Methods, Figures, Data Availability Section)
New materials and reagents need to be available; do any restrictions apply?	Yes	Materials and Methods, generation of lung cancer cell lines expressing cytokines

Antibodies	Information included in the manuscript?	In which section is the information available? (Reagents and Tools Table, Materials and Methods, Figures, Data Availability Section)
For **antibodies** provide the following information:<br>- Commercial antibodies: RRID (if possible) or supplier name, catalogue number and or/clone number<br>- Non-commercial: RRID or citation	Yes	In Materials and Methods

DNA and RNA sequences	Information included in the manuscript?	In which section is the information available? (Reagents and Tools Table, Materials and Methods, Figures, Data Availability Section)
**Short novel DNA or RNA including primers, probes**: provide the sequences.	Not Applicable	

Cell materials	Information included in the manuscript?	In which section is the information available? (Reagents and Tools Table, Materials and Methods, Figures, Data Availability Section)
**Cell lines:** Provide species information, strain. Provide accession number in repository **OR** supplier name, catalog number, clone number, and/**OR** RRID.	Yes	In materials and methods
**Primary cultures:** Provide species, strain, sex of origin, genetic modification status.	Not Applicable	
Report if the cell lines were recently **authenticated** (e.g., by STR profiling) and tested for mycoplasma contamination.	Yes	All cell lines are regularly tested by mycoplasma. All cell lines were recently acquired from the ATCC with an authentication document.

Experimental animals	Information included in the manuscript?	In which section is the information available? (Reagents and Tools Table, Materials and Methods, Figures, Data Availability Section)
**Laboratory animals or Model organisms:** Provide species, strain, sex, age, genetic modification status. Provide accession number in repository **OR** supplier name, catalog number, clone number, **OR** RRID.	Yes	In materials and methods
**Animal observed in or captured from the field:** Provide species, sex, and age where possible.	Not Applicable	
Please detail **housing and husbandry conditions**.	Yes	In materials and methods

Plants and microbes	Information included in the manuscript?	In which section is the information available? (Reagents and Tools Table, Materials and Methods, Figures, Data Availability Section)
**Plants:** provide species and strain, ecotype and cultivar where relevant, unique accession number if available, and source (including location for collected wild specimens).	Not Applicable	
**Microbes:** provide species and strain, unique accession number if available, and source.	Not Applicable	

Human research participants	Information included in the manuscript?	In which section is the information available? (Reagents and Tools Table, Materials and Methods, Figures, Data Availability Section)
If collected and within the bounds of privacy constraints report on age, sex and gender or ethnicity for all study participants.	Yes	Materials and methods

Core facilities	Information included in the manuscript?	In which section is the information available? (Reagents and Tools Table, Materials and Methods, Figures, Data Availability Section)
If your work benefited from core facilities, was their service mentioned in the acknowledgments section?	Not Applicable	

Design

Study protocol	Information included in the manuscript?	In which section is the information available? (Reagents and Tools Table, Materials and Methods, Figures, Data Availability Section)
If study protocol has been **pre-registered, provide DOI in the manuscript**. For clinical trials, provide the trial registration number **OR** cite DOI.	Not Applicable	
Report the **clinical trial registration number** (at ClinicalTrials.gov or equivalent), where applicable.	Not Applicable	

Laboratory protocol	Information included in the manuscript?	In which section is the information available? (Reagents and Tools Table, Materials and Methods, Figures, Data Availability Section)
Provide DOI OR other citation details if **external detailed step-by-step protocols** are available.	Not Applicable	

Experimental study design and statistics	Information included in the manuscript?	In which section is the information available? (Reagents and Tools Table, Materials and Methods, Figures, Data Availability Section)
Include a statement about **sample size** estimate even if no statistical methods were used.	Yes	Materials and methods, extensive statistical detail
Were any steps taken to minimize the effects of subjective bias when allocating animals/samples to treatment (e.g. **randomization procedure**)? If yes, have they been described?	Yes	In materials and methods
Include a statement about **blinding** even if no blinding was done.	Yes	In materials and methods
Describe **inclusion/exclusion criteria** if samples or animals were excluded from the analysis. Were the criteria pre-established?<br><br>If sample or data points were omitted from analysis, report if this was due to attrition or intentional exclusion and provide justification.	Yes	Described in materials and methods.
For every figure, are **statistical tests** justified as appropriate? Do the data meet the assumptions of the tests (e.g., normal distribution)? Describe any methods used to assess it. Is there an estimate of variation within each group of data? Is the variance similar between the groups that are being statistically compared?	Yes	All statistical requirements have been extensively described in materials and methods. Tests are reported for each figure.

Sample definition and in-laboratory replication	Information included in the manuscript?	In which section is the information available? (Reagents and Tools Table, Materials and Methods, Figures, Data Availability Section)
In the figure legends: state number of times the experiment was **replicated** in laboratory.	Yes	In the figure legend for the specific experiment, and in materials and methods for the number of replicates in the laboratory.
In the figure legends: define whether data describe **technical or biological replicates**.	Yes	In the figure legend and in materials and methods

Ethics

Ethics	Information included in the manuscript?	In which section is the information available? (Reagents and Tools Table, Materials and Methods, Figures, Data Availability Section)
Studies involving **human participants**: State details of **authority granting ethics approval** (IRB or equivalent committee(s), provide reference number for approval.	Yes	In materials and methods
Studies involving **human participants**: Include a statement confirming that **informed consent** was obtained from all subjects and that the experiments conformed to the principles set out in the WMA Declaration of Helsinki and the Department of Health and Human Services Belmont Report.	Yes	In materials and methods
Studies involving **human participants:** For publication of **patient photos**, include a statement confirming that consent to publish was obtained.	Not Applicable	
Studies involving experimental **animals**: State details of **authority granting ethics approva**l (IRB or equivalent committee(s), provide reference number for approval. Include a statement of compliance with ethical regulations.	Yes	In materials and methods
Studies involving **specimen and field samples:** State if relevant **permits** obtained, provide details of authority approving study; if none were required, explain why.	Not Applicable	

Dual Use Research of Concern (DURC)	Information included in the manuscript?	In which section is the information available? (Reagents and Tools Table, Materials and Methods, Figures, Data Availability Section)
Could your study fall under dual use research restrictions? Please check biosecurity documents and list of **select agents and toxins** (CDC): https://www.selectagents.gov/sat/list.htm	Not Applicable	
If you used a select agent, is the security level of the lab appropriate and reported in the manuscript?	Yes	In materials and methods
If a study is subject to dual use research of concern regulations, is the name of the **authority granting approval and reference number** for the regulatory approval provided in the manuscript?	Not Applicable	

Reporting

The MDAR framework recommends adoption of discipline-specific guidelines, established and endorsed through community initiatives. Journals have their own policy about requiring specific guidelines and recommendations to complement MDAR.

Adherence to community standards	Information included in the manuscript?	In which section is the information available? (Reagents and Tools Table, Materials and Methods, Figures, Data Availability Section)
State if relevant guidelines or checklists (e.g., **ICMJE, MIBBI, ARRIVE, PRISMA**) have been followed or provided.	Yes	ARRIVE guidelines, specified in materials and methods
For **tumor marker prognostic studies,** we recommend that you follow the **REMARK** reporting guidelines (see link list at top right). See author guidelines, under 'Reporting Guidelines'. Please confirm you have followed these guidelines.	Yes	REMARK guidelines, specified in materials and methods
For **phase II and III randomized controlled trials**, please refer to the **CONSORT** flow diagram (see link list at top right) and submit the CONSORT checklist (see link list at top right) with your submission. See author guidelines, under 'Reporting Guidelines'. Please confirm you have submitted this list.	Not Applicable	

Data Availability

Data availability	Information included in the manuscript?	In which section is the information available? (Reagents and Tools Table, Materials and Methods, Figures, Data Availability Section)
Have **primary datasets** been deposited according to the journal's guidelines (see 'Data Deposition' section) and the respective accession numbers provided in the Data Availability Section?	Not Applicable	
Were **human clinical and genomic datasets** deposited in a public access-controlled repository in accordance to ethical obligations to the patients and to the applicable consent agreement?	Not Applicable	
Are **computational models** that are central and integral to a study available without restrictions in a machine-readable form? Were the relevant accession numbers or links provided?	Not Applicable	
If publicly available data were reused, provide the respective **data citations in the reference list**.	Not Applicable	
